# Genomic Transition to Pathogenicity in Chytrid Fungi

**DOI:** 10.1371/journal.ppat.1002338

**Published:** 2011-11-03

**Authors:** Suzanne Joneson, Jason E. Stajich, Shin-Han Shiu, Erica Bree Rosenblum

**Affiliations:** 1 Department of Biological Sciences, University of Idaho, Moscow, Idaho, United States of America; 2 Department of Plant Pathology and Microbiology, University of California, Riverside, California, United States of America; 3 Department of Plant Biology, Michigan State University, East Lansing, Michigan, United States of America; University of Toronto, Canada

## Abstract

Understanding the molecular mechanisms of pathogen emergence is central to mitigating the impacts of novel infectious disease agents. The chytrid fungus *Batrachochytrium dendrobatidis* (*Bd*) is an emerging pathogen of amphibians that has been implicated in amphibian declines worldwide. *Bd* is the only member of its clade known to attack vertebrates. However, little is known about the molecular determinants of - or evolutionary transition to - pathogenicity in *Bd*. Here we sequence the genome of *Bd*'s closest known relative - a non-pathogenic chytrid *Homolaphlyctis polyrhiza* (*Hp*). We first describe the genome of *Hp*, which is comparable to other chytrid genomes in size and number of predicted proteins. We then compare the genomes of *Hp*, *Bd*, and 19 additional fungal genomes to identify unique or recent evolutionary elements in the *Bd* genome. We identified 1,974 *Bd*-specific genes, a gene set that is enriched for protease, lipase, and microbial effector Gene Ontology terms. We describe significant lineage-specific expansions in three *Bd* protease families (metallo-, serine-type, and aspartyl proteases). We show that these protease gene family expansions occurred after the divergence of *Bd* and *Hp* from their common ancestor and thus are localized to the *Bd* branch. Finally, we demonstrate that the timing of the protease gene family expansions predates the emergence of *Bd* as a globally important amphibian pathogen.

## Author Summary

The chytrid fungus *Batrachochytrium dendrobatidis* (*Bd*) is an emerging pathogen that has been implicated in decimating amphibian populations around the world. *Bd* is the only member of an ancient group of fungi (called the Chytridiomycota) that is known to attack vertebrates. The question of how an amphibian-killing fungus evolved from non-pathogenic ancestors is vital to protecting the world's remaining amphibians from *Bd*. We sequenced the genome of *Bd*'s closest known relative - a non-pathogenic chytrid named *Homolaphlyctis polyrhiza* (*Hp*). We compared the genomes of *Bd*, *Hp* and 18 additional fungi to identify what makes *Bd* unique. We identified a large number of *Bd*-specific genes, a gene set that contains a number of possible pathogenicity factors. In particular, we describe a large number of protease genes in the *Bd* genome and show that these genes were duplicated after the divergence of *Bd* and *Hp* from their common ancestor. Studying *Bd*'s pathogenesis in an evolutionary context provides new evidence for the role of protease genes in *Bd*'s ability to kill amphibians.

## Introduction

Understanding the emergence of novel pathogens is a central challenge in epidemiology, disease ecology, and evolutionary biology. Emerging pathogens of humans, wildlife, and agriculturally important crops generally have a dynamic recent evolutionary past. For example, many emerging pathogens have become adapted to new environmental conditions, shifted their host range, and/or evolved more virulent forms [1]–[3]. Identifying the genetic basis of these evolutionary shifts can lend insight into the mechanisms of pathogen emergence.

Studies of the amphibian-killing fungus *Batrachochytrium dendrobatidis* (*Bd*) provide an opportunity to better understand evolutionary transitions to pathogenicity. *Bd* is considered the leading cause of amphibian declines worldwide and is found on every continent where amphibians occur [4]–[5]. *Bd* infects amphibian skin and the resulting disease, chytridiomycosis, is responsible for population declines and extirpations in hundreds of amphibian species [6]–[7]. *Bd* is the only documented vertebrate pathogen in a diverse, early-branching lineage of fungi called the Chytridiomycota. Some chytrids are pathogens of plants, but most chytrids are primarily known to survive on decaying organic material as saprobes [8]. The question of how an amphibian-killing fungus evolved from an ancestor that was not a vertebrate pathogen is vital to understanding and mitigating the chytridiomycosis epidemic and will also shed light on the evolution of novel pathogens more broadly.

Investigating the transition to pathogenicity in chytrid fungi requires an explicitly evolutionary perspective. Specifically, identifying elements of the genome that have undergone recent evolution in the branch leading to *Bd* may help us determine how *Bd* attacks its amphibian hosts. Previously we identified several families of proteases that may be involved in *Bd*'s ability to infect amphibian skin. Specifically, we found expanded gene families of metallo- and serine proteases in the *Bd* genome that exhibit life-stage specific gene expression patterns [9]. These proteases have been hypothesized to play a role in the ability of other fungal pathogens to invade and degrade host tissue [10]–[13]. However, previous studies could not resolve if these gene family expansions occurred along the branch leading to *Bd* because the fungal genomes available for comparison were only distantly related to *Bd*.

To determine what unique features of the *Bd* genome might relate to its ability to colonize amphibian skin, we compared genomes of *Bd* and its closest known relative, *Homolaphlyctis polyrhiza* (Hp) (this isolate has been described by Joyce Longcore (pers. comm.) and has been referred to as “JEL142” in previous publications [8]). *Bd* and *Hp* are in the same Rhizophydiales order [14], and *Bd* is the only member of this clade known to be a vertebrate pathogen [8]. We first confirmed that *Hp* cannot survive on amphibian skin alone. We then sequenced and characterized the genome of *Hp* using Roche-454 pyrosequencing. Finally, we used a comparative genomics approach to identify differences between *Bd* and *Hp* using additional fungal species as outgroups. Based on identified unique elements of the *Bd* genome, we develop hypotheses for the mechanisms and evolution of *Bd* pathogenicity.

## Materials and Methods

### Taxon Sampling

Our focal isolates were the JAM81 strain of *Bd* and the JEL142 strain of *Hp*. JAM81 was isolated from *Rana muscosa* in the Sierra Nevada Mountains in California, where *Bd* has caused catastrophic declines in *R. muscosa* populations [15]. *Hp* was collected from leaf litter in Maine and is a presumed saprobe. We also used the information from publically available genomes of an additional *Bd* isolate - JEL423 (http://www.broadinstitute.org/annotation/genome/batrachochytrium_dendrobatidis/MultiHome.html), andan additional chytrid, *Spizellomyces punctatus*, a terrestrial saprobe (Origins of Multicellularity Sequencing Project, Broad Institute of Harvard and MIT (http://www.broadinstitute.org/)). Finally we used the genome information from 17 additional publicly available fungal genomes (Table S1). We chose these outgroups to represent a broad phylogenetic survey of fungi that span four additional fungal phyla: Blastocladiomycota (*Allomyces macrogynus*), Zygomycota (*Phycomyces blakesleeanus)*, Basidiomycota (*Coprinopsis cinerea*, *Cryptococcus neoformans*, *Puccinia graminis* f. sp. *tritici*, *Ustilago maydis*), and Ascomycota (*Arthroderma benhamiae*, *Aspergillus nidulans*, *Blastomyces dermatitidis*, *Botrytis cinerea*, *Coccidioides immitis*, *Fusarium graminearum*, *Microsporum canis*, *Neurospora crassa*, *Pyrenophora tritici-repentis*, *Trichophyton rubrum*, and *Uncinocarpus reesii*). *Arthroderma benhamiae*, *M. canis*, and *T. rubrum* were chosen in particular because they are dermatophytes (i.e. fungal pathogens that infect skin).

We reconstructed the phylogenetic relationships among the 19 taxa used in this study using Bayesian phylogenetic analyses of 51 single-copy genes. The alignment was comprised of 21182 total trimmed amino acid residues. The orthologous sequences were aligned with T-Coffee [16], concatenated, and trimmed with trimAl [17]. The Basidiomycota phylum was constrained by members *Ustilago maydis* and *Puccinia graminis*, and the tree rooted with the Chytridiomycota clade based on [8]. Bayesian posterior probabilities are shown below internal nodes and ML bootstrap values from 100 replicates above the nodes.

### Growth of *Bd* and *Hp* on Amphibian Skin

We grew *Bd* (JAM81) and *Hp* on the standard growth medium PmTG (made from peptonized milk, tryptone and glucose) [18]. After one week of growth, we transferred 3.8× 10^6^ zoospores from each isolate to 3 mL of two liquid growth conditions: standard growth media and amphibian skin. For standard growth media we used 1% liquid PmTG, and for amphibian skin we used 10% w/v pulverized and autoclaved cane-toad skin in water. We established six technical replicates of each isolate in each condition. Liquid cultures were gently shaken in 6-well tissue culture plates. To test how long *Bd* and *Hp* survived in each growth condition, we tested an aliquot from each culture every day for 14 days. Each day we removed 15 µL from each of the technical replicates, pooled aliquots for each isolate in each treatment group, and inoculated PmTG-agar growth plates. We inspected growth plates every day using 200× magnification to visualize whether active zoospores were produced.

### *Hp* Genome Sequence, Assembly, and Annotation

We grew *Hp* at room temperature (23–25C) in liquid PmTG medium with gentle agitation for approximately 2 weeks. We extracted *Hp* DNA using a Zolan and Pukkila [19] protocol modified by the use of 2% sodium dodecyl sulphate as extraction buffer in place of CTAB. We sequenced the *Hp* genome using a Roche 454 Genome Sequencer FLX with Titanium chemistry and standard Roche protocol. We screened and trimmed 1,100,797 reads of vector sequences and assembled them with Roche's GS De Novo Assembler. We improved the assembly by synteny-based alignment to the JAM81 genome sequence with Mercator [20].

We annotated the *Hp* genome with predicted proteins using the MAKER annotation pipeline [21]. MAKER predicts proteins based on homology with protein-coding sequences of other species, and with the consensus of the *ab initio* gene prediction algorithms GeneMark, AUGUSTUS, and SNAP. GeneMark is self training so we simply applied it to determine *ab initio* parameters. We trained AUGUSTUS using parameters provided in the MAKER package and previously determined *Bd* training parameters. We trained SNAP by iteratively running MAKER with SNAP *Bd* models and then retraining on the most confident gene model parameters from the initial run. All parameters files are available in http://fungalgenomes.org/public/Hp_JEL142/. Because MAKER's final set of predicted proteins (referred to hereafter as “Hp_Maker”) is a conservative estimate that relies upon the consensus of different prediction algorithms, we also used the set of *ab initio* predicted proteins in MAKER by GeneMark-ES [22] as an upper limit (referred to hereafter as “Hp_GeneMark”). Hp_Maker is not a perfect subset of Hp_GeneMark, so we considered both datasets when characterizing the proteome of *Hp*. We annotated *Hp* protein models by comparison to the Pfam database of protein domains [23] using HMMER 3.0 (http://hmmer.org/).

We used two methods that rely on different algorithms to confirm that we successfully identified the majority of *Hp* proteins. First, we used the eukaryotic genome annotation pipeline CEGMA to predict the number of core eukaryotic genes in the *Hp* alignment [24]. Second we determined the number of “chytrid-specific” orthologous groups that were present in the *Hp* genome. We defined chytrid-specific orthologous groups as those groups shared between all available Chytridiomycota genomes: two *Bd* isolates (JAM81 and JEL423) and one *Spizellomyces punctatus* isolate (DAOM BR117) (Table S1). We identified chytrid-specific orthologous groups using BLASTP [25] and OrthoMCL [26], and determined how many of these were also found within either set of *Hp* predicted proteins (i.e., Hp_Maker and Hp_GeneMark).

### *Bd* Unique Genomic Features

We also used BLASTP and OrthoMCL to determine orthologous groups for all sampled taxa. These orthologous groups were used to determine “*Bd*-specific” genes which we defined as those groups or genes that were present in both sequenced *Bd* genomes (JAM81 and JEL423) but absent from all other sampled fungi. [Note that the *Bd*-specific gene set is distinct from the more broadly defined chytrid-specific gene set discussed above]. We used GO::TermFinder [27] to determine if the Pfam annotations for the set of *Bd-*specific genes showed enrichment for particular GO terms.

### *Bd* Gene Family Expansions

We identified several gene family expansions in *Bd* through inspection of the top ten largest *Bd*-specific orthologous groups and inspection of enriched GO categories. We found gene family expansions in families with genes containing M36, S41, and Asp (both Asp and Asp_protease) protease Pfam signature domains (see Table S2 for sequences and their Pfam domain delimitation). We conducted an exhaustive search in the focal genomes for M36, S41, and Asp domains using HMMER3 (http://hmmer.org/). For *Hp* we conducted the HMMER3 search in both the MAKER and GENEMARK datasets. For S41 and Asp, the predicted proteins from Maker were subsets of those from GeneMark, so we only report GeneMark names. For M36 there were several Maker predicted proteins that were not included in the GeneMark set, so we report both Maker and GeneMark names. We then aligned the sequences of the protein domains for all members in each expanded family for the three Chytridiomycota genomes (*Bd*, *Hp*, and *Spizellomyces punctatus*) and one Blastocladiomycota outgroup (*Allomyces macrogynus*). We generated these alignments using the iterative alignment program MUSCLE [28]. After inspecting the alignments, we found that 8 M36 and 13 Asp protein sequences were missing >50% of their domain sequences. These partial sequences were likely mis-annotation or pseudogenes so we excluded them from further analysis (see Table S2B for identities of excluded partial sequences). After aligning the protein domain sequences of the remaining proteins (see Figure S1 for alignments), we reconstructed gene trees for each family using the Maximum Likelihood method implemented in RAxML [29]. We used the rapid bootstrap algorithm (400 replicates) with the Jones-Taylor-Thornton substitution matrix assuming a gamma model of rate heterogeneity. We report the Maximum Likelihood trees with the highest log likelihood score and bootstrap support values.

We calculated synonymous and non-synonymous substitution rates (*Ks* and *Ka*, respectively) with the yn00 program implemented in the PAML package [30] using full length annotated coding sequences. For each expanded protease gene family (containing M36, S41, and Asp domains) we calculated *Ks* and *Ka* of putative orthologs between all focal taxa pairs [i.e., chytrids (*Bd*, *Hp*, and *Spizellomyces punctatus*) and between all focal taxa and the outgroup (*Allomyces macrogynus*)]. We identified putative orthologs based on a cross-species reciprocal best match between any species pairs [31]. In addition, we used a second, more stringent approach that required sequence distances between reciprocal best matches to follow the relationships between the four focal species. Because the rate distributions from these two approaches were similar, we only report results from the first approach. Because yn00 does not robustly correct for multiple substitutions [32], and because *Ks* values are large between our focal taxa, we use *Ks* values to make a general comparison (within versus between species) for rates of molecular evolution.

We made rough divergence time estimates for the duplication events in the three expanded protease gene families using “node-*Ks*” as a proxy of time. The node *Ks* is defined as follows: for each node N in the mid-point rooted phylogeny, its *Ks* is the averaged *Ks* values between all operational taxonomic unit pairs across the two lineages that originated from N. There are no empirical estimates of chytrid substitution rates, so we do not propose specific dates for the duplication events. However, we do use a rough approximation for a reasonable substitution rate (following previous molecular evolution studies in fungi [33]) to test whether the timing of gene duplications was likely coincident with the emergence of *Bd* as a deadly amphibian pathogen.

## Results

### Taxon Sampling

**Figure 1:**
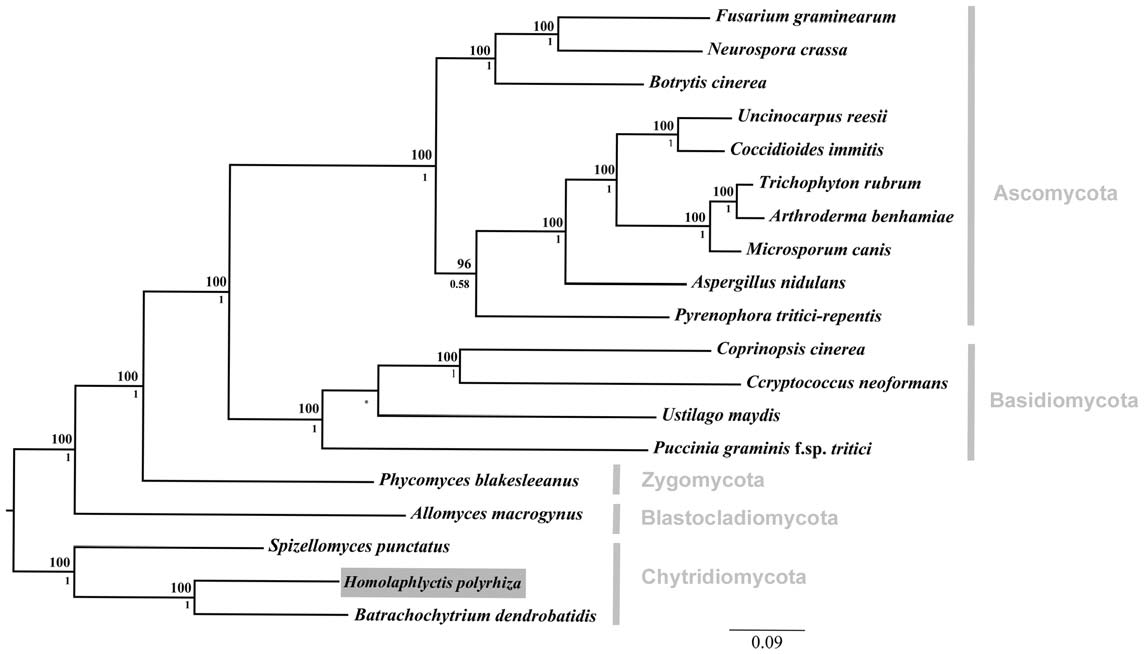
Phylogenetic relationships among the 19 taxa used in comparative genomics analyses. Focal taxon, *Hp*, boxed in grey. We compared the *Hp* genome to the genome of the amphibian pathogen *Bd* and to a diverse group of other fungal genomes including representatives from all major fungal lineages. Phylogenetic relationship constrained at node between *U. maydis* and *P. graminis* and marked with an asterisk. Bayesian posterior probabilities are shown below internal nodes and ML bootstrap values from 100 replicates are shown above the nodes.

The phylogenetic relationship among all 19 taxa in this study can be seen in Figure 1. As described above, we sampled genomes from across the diversity of five fungal phyla (i.e., Chytridiomycota, Blastocladiomycota, Zygomycota, Basidiomycota, Ascomycota). Our sampling scheme allowed us to determine, in a phylogenetic context, which elements of *Bd*'s genome are shared with *Hp* and other fungal taxa.

### Growth of *Bd* and *Hp* on Amphibian Skin

**Figure 2:**
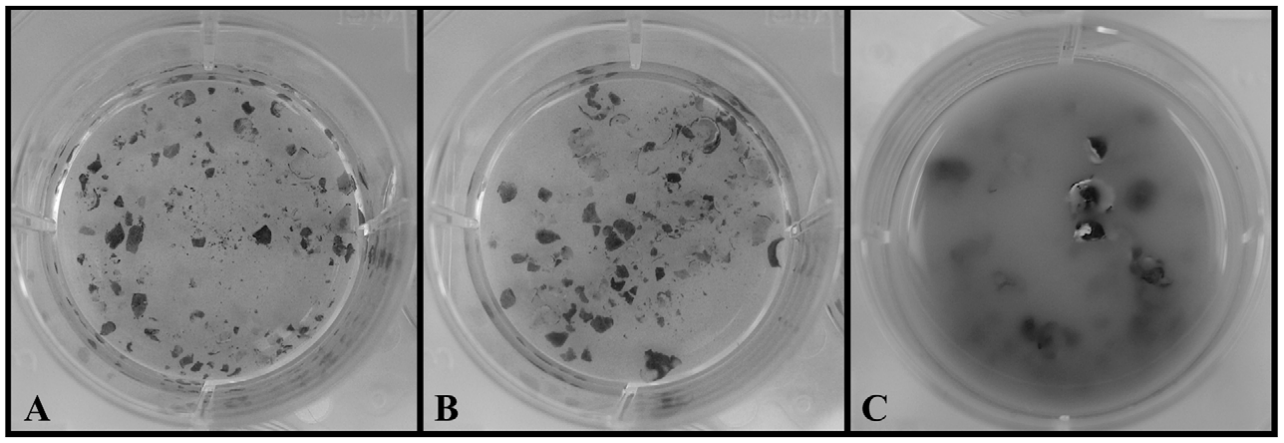
Chytrid growth on cane-toad-skin. A. Negative control (no chytrid): intact skin after 14 days. B. *Hp* treatment: intact skin and no *Hp* growth after 14 days. C. *Bd* treatment: degraded skin and *Bd* growth after 14 days.

Both *Bd* and *Hp* grew well in standard PmTG growth media and produced viable zoospores throughout the entire 14 day observation period. However, only *Bd* survived on frog skin alone. *Bd* produced viable zoospores in the cane-toad skin treatment throughout the entire observation period, and after 14 days of incubation the *Bd* - frog skin solution was cloudy with chytrid growth and degraded skin (Figure 2). Conversely, *Hp* did not survive and reproduce on cane-toad skin alone. We observed viable zoospores for *Hp* in the cane-toad skin treatment only for the first three days (these zoospores most likely persisted from the initial inoculation), and after 14 days of incubation the *Hp* - cane-toad skin solution remained clear of chytrid growth and the cane-toad skin remained intact and not further degraded (Figure 2). We did not observe the growth of any bacterial or fungal contaminants in any of the treatments.

### *Hp* Genome Sequence, Assembly, and Annotation

We achieved a roughly 11.2× coverage of the *Hp* genome (total number of aligned bases divided by final genome length, assuming that most of the genome is represented in the aligned reads). We assembled 922,085 screened and trimmed sequencing reads into 16,311 contigs (N50 = 36,162). We inferred a haploid genome size for *Hp* of 26.7 Mb, comparable to other Chytridiomycota genomes [*Bd* (JAM81) = 24.3 Mb, and *Spizellomyces punctatus* = 24.1 Mb]. We have deposited the *Hp* 454 reads in GenBank through the NCBI Sequence Read Archives under the accession SRA037431.1), and we have deposited the Whole Genome Shotgun project at DDBJ/EMBL/GenBank under the accession AFSM00000000 (the version described here is the first version, AFSM01000000).

We generated 5,355 high confidence MAKER predictions and 11,857 GeneMark *ab initio* predictions for *Hp*'s protein coding genes. The number of predicted *Hp* proteins falls within the range of other annotated chytrid genomes (8,732 predicted proteins in *Bd* (JAM81) and 8,804 in *Spizellomyces punctatus*). The difference in number of *Hp* predicted protein numbers between MAKER and GeneMark is due to MAKER's conservative approach, which relies upon homology with protein-coding sequences of other species, and with the consensus of multiple *ab initio* gene prediction algorithms. We did not directly validate the number of expressed genes in our predicted protein sets with EST or RNA sequencing. However, we did compare the *Hp* predicted protein set to gene content in other species, which provides confidence in the *Hp* annotation and assembly. We recovered 92% (228/249) of the core eukaryotic genes using CEGMA in the Hp_Maker dataset. Similarly, we identified 3,216 orthologous groups of “chytrid-specific” proteins shared among both *Bd* isolates and *S. punctatus* (Table S3). Of the predicted chytrid-protein set we recovered 90% (2,885/3,216) in one or both *Hp* predicted protein sets (2,271 in Hp_Maker and 2,817 in Hp_GeneMark). Together, these results indicate that our sequencing efforts recovered a large proportion of genes that are predicted to occur in the *Hp* genome.

### *Bd* Unique Genomic Features

We identified *Bd*-specific genes using the genomes of *Hp* and 17 additional fungi. We considered genes to be *Bd*-specific if they were present in orthologous groups in both sequenced *Bd* genomes (JAM81 and JEL423) and absent from all other fungi including *Hp*. Using OrthoMCL clustered proteins we defined 6,556 orthologous groups in *Bd* (Table S4). Of the 6,556 orthologous groups in *Bd*, 1700 were *Bd*-specific by the above definition. The *Bd*-specific orthologous groups were comprised of 1,974 protein encoding genes, 417 (21%) of which could be functionally categorized by a Pfam domain (with an e-value<0.01) (Table S4). We did not find any orthologous groups uniquely shared between *Bd* and the dermatophytes to the exclusion of all other fungal outgroups (Table S4). Although we defined orthologous groups using the sequenced genomes of both *Bd* isolates (JAM81 and JEL423), below we report gene IDs from JAM81 for simplicity.

We conducted enrichment analyses using Gene Ontology (GO) terms from the set of 417 *Bd*-specific genes associated with a Pfam domain and found enrichment in all 3 GO structured vocabularies: Cellular Component, Biological Process, and Molecular Function. We present all significantly enriched GO terms (with a corrected P-value of < = 0.05) for the *Bd*-specific gene set in Table 1. Briefly, in the Biological Process ontology we found enrichment for genes involved in metabolic processes and regulation of carbohydrates, proteins, and transcription. In the Cellular Component ontology we found enrichment of genes located extracellularly, in the nucleus, and in membranes. In the Molecular Function ontology we found enrichment for genes involved in zinc-ion binding, protein dimerization, DNA-binding, hydrolase activity, and protease and triglyceride lipase activity.

**Table 1:** The enrichment of Cellular Component, Biological Process and Molecular Function GO terms of 417 *Bd* specific genes associated with a Pfam domain.

GOID	Term	corrected p-value	# in *Bd* specific gene set	# in *Bd* genome
**Biological Process**				
GO:0006508	proteolysis	1.3E-57	99	292
GO:0019538	protein metabolic process	1.6E-31	126	845
GO:0044238	primary metabolic process	5.5E-29	179	1644
GO:0043170	macromolecule metabolic process	1.0E-26	147	1229
GO:0008152	metabolic process	4.5E-24	196	2075
GO:0019219	regulation of nucleobase, nucleoside, nucleotide and nucleic acid metabolic process	6.6E-17	46	205
GO:0051171	regulation of nitrogen compound metabolic process	6.6E-17	46	205
GO:0009889	regulation of biosynthetic process	8.2E-17	43	180
GO:0010556	regulation of macromolecule biosynthetic process	8.2E-17	43	180
GO:0031326	regulation of cellular biosynthetic process	8.2E-17	43	180
GO:2000112	regulation of cellular macromolecule biosynthetic process	8.2E-17	43	180
GO:0031323	regulation of cellular metabolic process	1.8E-16	46	210
GO:0080090	regulation of primary metabolic process	1.8E-16	46	210
GO:0045449	regulation of transcription	2.3E-16	42	176
GO:0060255	regulation of macromolecule metabolic process	6.0E-16	43	189
GO:0010468	regulation of gene expression	2.1E-15	42	186
GO:0019222	regulation of metabolic process	4.9E-15	46	227
GO:0065007	biological regulation	6.8E-15	67	454
GO:0050789	regulation of biological process	1.5E-14	66	449
GO:0050794	regulation of cellular process	3.3E-14	62	409
GO:0006355	regulation of transcription, DNA-dependent	3.4E-14	35	139
GO:0051252	regulation of RNA metabolic process	3.4E-14	35	139
GO:0051704	multi-organism process	3.6E-2	5	12
GO:0005975	carbohydrate metabolic process	4.5E-2	26	253
**Cellular Component**				
GO:0005623	cell	1.6E-17	150	1571
GO:0044464	cell part	1.6E-17	150	1571
GO:0005622	intracellular	5.5E-10	105	1149
GO:0043231	intracellular membrane-bounded organelle	4.4E-08	51	425
GO:0043227	membrane-bounded organelle	5.2E-08	51	427
GO:0005634	nucleus	1.2E-07	38	272
GO:0043229	intracellular organelle	1.9E-05	61	659
GO:0043226	organelle	2.1E-05	61	661
GO:0016021	integral to membrane	3.9E-05	33	271
GO:0016020	membrane	1.0E-04	58	645
GO:0044425	membrane part	1.2E-04	40	381
GO:0031224	intrinsic to membrane	1.6E-04	33	288
GO:0005576	extracellular region	2.3E-04	14	69
GO:0044421	extracellular region part	1.4E-03	12	60
GO:0044424	intracellular part	7.6E-03	66	880
**Molecular Function**				
GO:0004190	aspartic-type endopeptidase activity	9.0E-93	82	98
GO:0070001	aspartic-type peptidase activity	9.0E-93	82	98
GO:0070011	peptidase activity, acting on L-amino acid peptides	5.0E-63	102	282
GO:0004175	endopeptidase activity	1.1E-61	87	198
GO:0008233	peptidase activity	1.3E-60	102	296
GO:0016787	hydrolase activity	4.1E-32	143	1056
GO:0003824	catalytic activity	2.2E-21	200	2254
GO:0001071	nucleic acid binding transcription factor activity	3.6E-20	32	79
GO:0003700	sequence-specific DNA binding transcription factor activity	3.6E-20	32	79
GO:0046914	transition metal ion binding	1.8E-14	56	342
GO:0008270	zinc ion binding	1.5E-13	47	261
GO:0005488	binding	2.2E-13	164	1964
GO:0043565	sequence-specific DNA binding	2.5E-12	20	49
GO:0046872	metal ion binding	3.3E-11	57	417
GO:0043167	ion binding	4.1E-11	57	419
GO:0043169	cation binding	4.1E-11	57	419
GO:0003676	nucleic acid binding	1.8E-09	70	633
GO:0003677	DNA binding	6.3E-09	43	298
GO:0005515	protein binding	1.9E-06	44	370
GO:0008236	serine-type peptidase activity	2.7E-04	16	85
GO:0017171	serine hydrolase activity	2.7E-04	16	85
GO:0016810	hydrolase activity, acting on carbon-nitrogen (but not peptide) bonds	9.8E-04	11	46
GO:0046983	protein dimerization activity	1.6E-03	9	32
GO:0004871	signal transducer activity	3.4E-03	11	52
GO:0060089	molecular transducer activity	3.4E-03	11	52
GO:0004806	triglyceride lipase activity	3.3E-02	6	20

**Figure 3:**
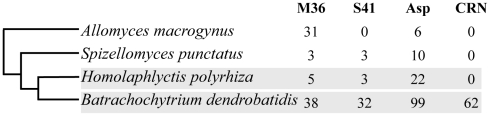
Gene family copy numbers for metalloproteases (M36), serine-type proteases (S41), aspartyl proteases (ASP) and CRN-like proteins (CRN) in the Chytridiomycota (*Bd*, *Hp* and *S. punctatus*), and a Blastocladiomycota outgroup (*A. macrogynus*). Phylogenetic relationship of taxa adapted from [8]. Focal taxa highlighted in grey.

Within the set of *Bd*-specific and GO-enriched genes were several functional groups of particular interest for their possible role in *Bd* pathogenesis. First, many *Bd*-specific genes were proteases and were found in expanded gene families (see below). Second, the *Bd*-specific gene set was enriched for genes containing the Lipase_3 Pfam domain found in triacylglyceride lipases (6 of 417 in the *Bd*-specific gene list, vs 20 of 8732 in the genome, p<0.03) (BATDEDRAFT 93190, BATDEDRAFT 26490, BATDEDRAFT_ 86691, BATDEDRAFT 93191, BATDEDRAFT_89307, BATDEDRAFT_26489). Third, we identified 62 genes from the *Bd*-specific gene set that encode Crinkler or CRN-like microbial effectors (CRN), a class of genes previously reported only in oomycetes and not found in any of the other fungi considered here (Figure 3 and Table S5).

### *Bd* Gene Family Expansions

**Figure 4:**
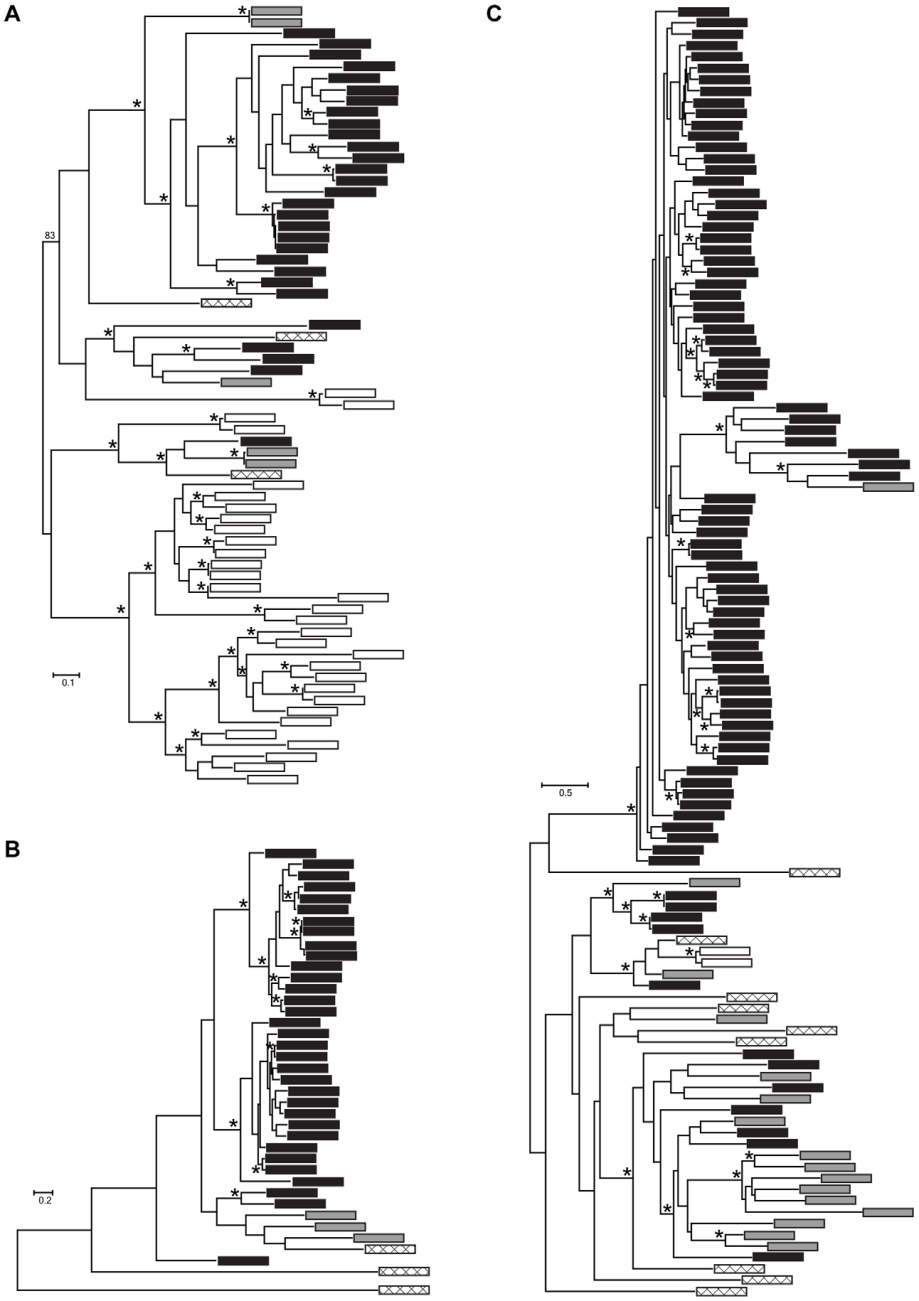
Maximum likelihood phylogenies of gene families containing (A) M36, (B) S41, and (C) Asp Pfam domains. Each tip represents a single gene copy and each source species is denoted by shaded or hatched boxes (*Bd*: black, *Hp*: grey, *S. punctatus*: hatched, *A. macrogynus*: white). Bootstrap values over 80% indicated with asterisks at internal nodes.

We conducted more detailed analyses for three protease gene families that were identified in the *Bd*-specific gene set and showed GO term enrichment: metallo-, serine-type, and aspartyl proteases (M36, S41, and Asp Pfam domains, respectively). The *Bd* genome contained 38 metalloproteases, 32 serine-type proteases, and 99 aspartyl proteases, in all cases at least 4 times as many family members as *Hp* (Figure 3). We found that expansions of metalloproteases, serine-type proteases and aspartyl proteases were largely *Bd* specific, having occurred after the split between the *Bd* and *Hp* lineages from their most recent common ancestor (Summary in Figure 4, gene-names available in tree, Figure S2). In all three families, *Bd* had a greater number of gene copies than any of the other focal taxa, and the *Bd* gene copies were generally clustered together to the exclusion of homologues from other taxa. This clustering is consistent with lineage-specific gene family expansions in *Bd* (Figure 4). We observed a large number of metalloprotease genes not only in *Bd* but also in *Allomyces macrogynus* (38 and 31 gene family members, respectively) (Figure 3). However, the gene tree indicates that the expansion of metalloprotease genes in *Bd* and *A. macrogynus* were independent with most duplication events occurring after the divergence of *Bd* and *A. macrogynus* from their common ancestor (Figure 4A).

**Figure 5:**
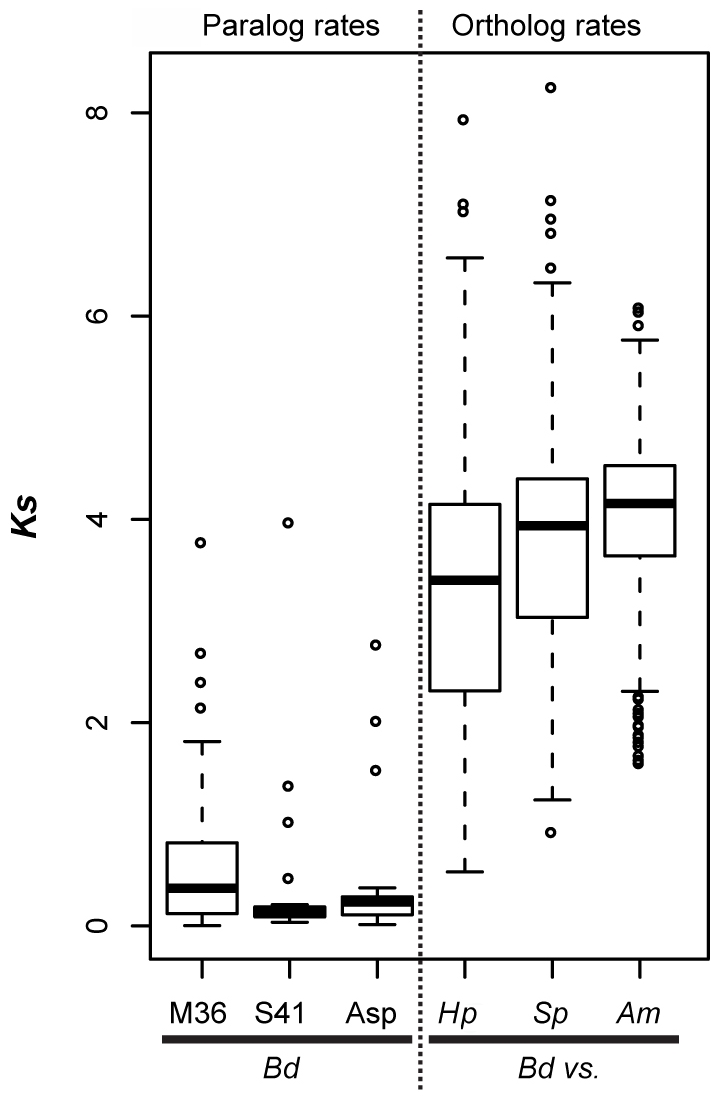
Left panel (paralog rates) shows box plots of synonymous substitution rates (*Ks*) for *Bd* lineage-specific duplicates in three protease families. Right panel (ortholog rates) shows box plots of *Ks* values for putative orthologs between *Bd* and *Hp*, *Bd* and *S. punctatus*, and *Bd* and *A. macrogynus*. Box and whisker plots show median (line), inter-quartile range (box), 1.5 inter-quartile range (whiskers), and outliers (open circles).

In addition to identifying many lineage-specific duplicates of proteases in *Bd*, we demonstrate that these *Bd* duplication events likely occurred significantly more recently than the divergence time between the species analyzed (Figure 5). To assess the timing of expansion in each protease gene family, we calculated synonymous substitution rate, *Ks*, between homologs and based on the phylogeny, we calculated a node *Ks* value for each lineage-specific duplication node (Figure 5, left panel). The median *Ks* values for the metallo-, serine-type, and aspartyl – proteases derived from *Bd-*specific duplications were 0.37, 0.14 and 0.24, respectively (Figure 5, left panel). We note that in the metalloprotease family there were similar numbers of lineage-specific duplications in *Bd* (24) and *A. macrogynus* (28). However the *Ks* values of *Bd-*specific duplicates were significantly lower *A. macrogynus* duplicates (median *Ks*: 0.37 and 1.56, respectively; Kolmogorov-Smirnov tests, *p<*4.6e-5), indicating that *Bd-*specific M36 duplications took place much more recently than the *A. macrogynus* duplications.

We also examined *Ks* values of putative orthologs between *Bd* and *Hp*, *Bd* and *S. punctatus*, and *Bd* and *A. macrogynus* (Figure 5B, right panel). As expected from the phylogenetic relationships of these four species [8], the median *Ks* for all species was high, but the median *Ks* for *Bd-Hp* (3.40) was significantly lower than that of *Bd-S. punctatus* (3.94) and *Bd-A. macrogynus* (4.03) (Kolmogorov-Smirnov tests, *p<*2.2e-16). Importantly, the median *Bd-Hp* orthologous *Ks* values were ∼9–24 fold higher than the median *Ks* of *Bd* lineage-specific duplicates. Therefore, the *Bd*-specific duplications occurred substantially more recently than the divergence of *Bd* and *Hp*. Previous molecular evolution studies in fungi have used a synonymous nucleotide substitution rate of 8.1e-9 substitutions per site per year to estimate the timing of molecular events [33]. If this substitution rate is reasonable for chytrid fungi, the duplication events leading to the metallo-, serine-type, and aspartyl protease gene family expansions in *Bd* would be millions of years old (Table S6). Even if the true substitution rate differs by several orders of magnitude, it is important to recognize that these protease duplication events occurred long before *Bd* emerged as a global pathogen of amphibians.

## Discussion

To investigate the genomic changes that accompanied the evolution of pathogenicity, we compared *Bd*, the deadly chytrid pathogen of amphibians, with *Hp*, a closely related chytrid that is not a known pathogen of vertebrates. We confirm that *Bd* and *Hp* have different nutritional modes (Figure 2); unlike *Hp*, *Bd* is capable of growing on amphibian skin alone. Given the most chytrids are saprobes like *Hp*, *Bd*'s ability to infect vertebrate skin likely arose after the divergence of *Bd* and *Hp* from their common ancestor. Fungal growth on vertebrate skin requires the expression of enzymes that break-down host epidermal tissue [10]–[13]. Because *Bd* causes chytridiomycosis by infecting frog skin [34]–[35] we were particularly interested in elements of the *Bd* genome whose evolution might have allowed *Bd* to colonize and degrade amphibian skin.

We compared the genomes of *Bd* and *Hp* in a broad taxonomic context of 18 diverse fungal genomes to identify genomic factors that make *Bd* unique. The *Bd* and *Hp* genomes are similar in size and number of predicted genes but show important differences in gene content. Therefore we could identify *Bd*-specific genes (i.e., genes that were found in *Bd* but not in *Hp* or other fungal outgroups). *Bd*-specific genes are enriched for GO terms related to extracellular and enzymatic activity. Many *Bd*-specific genes are members of recently expanded gene families (i.e., gene families with significantly more members than other fungal species). Below we discuss *Bd*-specific genes with particular emphasis on understanding how *Bd* may interact with its amphibian hosts.

Proteases are the most dramatically enriched class of *Bd-*specific genes. The *Bd* genome contains expanded gene families of metalloproteases, serine-type proteases, and aspartyl proteases. Each of these *Bd* gene families contains more than 30 family members and contains 4–10 times as many family members as found in *Hp* (Figure 3). Extracellular fungal proteases have been implicated in the adherence to, invasion of, and degradation of host cells by other fungal pathogens [10]–[13]. In particular, protease gene family expansions have been suggested as a link to pathogenesis in other fungal pathogens. Several fungal pathogens of vertebrates (e.g., *Arthroderma benhamiae*, *Coccidioides* spp, and *Trichophyton* spp.) exhibit gene family expansions specifically for metalloproteases and serine-type proteases [10], [36]–[37].

Here we strengthen the evidence implicating proteases in *Bd* pathogenesis in several ways. First and most importantly we demonstrate that protease gene families are not expanded in *Hp* and thus polarize the expansion events to a much shorter phylogenetic branch leading to *Bd*. Second, we present an additional protease gene family expansion. We previously reported the *Bd* gene family expansions for metallo- and serine proteases [9], and we now describe a dramatic expansion of aspartyl proteases in the *Bd* genome (Figure 3). Aspartyl proteases are of particular interest because they have been implicated in the adherence to and invasion of human host tissue by fungal pathogens (*Candida* spp.) [38]–[39]. Many genes in the expanded metallo -, serine - and aspartyl - protease gene families are highly expressed, and in some cases differentially expressed between *Bd* life stages [9]. Finally, we more rigorously document the dynamics of protease gene family expansions. Calculations using a range of reasonable substitution rates show that the three protease gene family expansions occurred substantially more recently than the divergence of *Bd* and *Hp* from their common ancestor.

It is important to caution that our comparative genomics results do not conclusively demonstrate a role for proteases as pathogenicity factors. First, we lack a specific mechanism by which proteases mediate *Bd* host invasion. Understanding the functional consequences of protease gene family expansions will require molecular assays to determine how specific enzymes contribute to host substrate metabolism. Second, protease gene family expansions are not always obviously correlated with fungal pathogenicity. For example we observed a large number of metalloprotease genes in *Allomyces macrogynus* and a variable number of aspartyl proteases in several of our outgroup taxa (Figure 4). These are independent expansion events relative to the *Bd* gene duplications and are not associated with a specific shift in substrate metabolism. Third, the estimated timing of the *Bd* protease gene duplications does not unambiguously link particular genes to the recent emergence of *Bd* as a global frog pathogen. Although the gene duplication events are relatively recent, most still likely occurred millions of years ago. More ancient duplication of protease genes may have set the stage for *Bd*'s ability to infect frogs, but finer scale intraspecific data will be required to determine whether particular paralogs exhibit molecular signatures of recent selection.

While proteases may play the most obvious role in pathogen invasion and metabolism of host tissue, we also observed an enrichment of genes with triglyceride lipase activity in the *Bd*-specific gene set (Table 1). These enzymes are known to play a role in fungal-plant interactions [40], and have been hypothesized to play a role in at least one fungal-vertebrate interaction - between *Malassezia furfur* and the skin of its human host [41]. *M. furfur* incorporates host lipids into its own cell wall; this is thought to assist *M. furfur* in adhering to the host and evading the host's immune system. The extent to which *Bd* can utilize the products of triglyceride lipase activity for nutrition or adhesion remains to be tested. However, the enrichment of triglyceride lipase genes in the *Bd*-specific gene set suggests considering whether lipases could play a role in *Bd*'s invasion of host tissue.

In addition to the genes that may be involved in host tissue metabolism we observed a large number of *Bd*-specific genes with similarity to microbial proteins known as Crinklers and Crinkler-like effectors (CRN). Microbial effectors in general act within the host cytoplasm to suppress host defenses and alter normal host cell metabolism [42]–[43]. It is unusual that a fungus contains CRN effectors as these proteins have so far only been reported from oomycetes, a group of important plant and fish pathogens within the kingdom Chromista [44]. CRN effectors are modular proteins consisting of a signal-peptide, a downstream translocator domain that allows CRN proteins to gain entry into host cells, and a C-terminus domain that interacts with host proteins [42]. While 62 unique *Bd* proteins show similarity to CRN effectors at the protein level, only one is predicted to be secreted (BATDEDRAFT_ 23205). Therefore while the function of putative CRN effectors in *Bd* remains to be determined, the possibility that they function as microbial effectors and interact with host elements merits further investigation.

We have sequenced the genome of *Bd*'s closest known relative to develop hypotheses for genomic determinants of *Bd*'s ability to infect and kill amphibians. However, the divergence between *Bd* and *Hp* is still substantial [8]. Recent research indicates that chytrids may be more ubiquitous than previously appreciated in both aquatic and terrestrial environments [45], and much chytrid diversity remains to be characterized. The discovery of additional taxa more closely related to *Bd* than *Hp* would help further localize genomic changes to the *Bd* lineage. Interspecific comparisons such as the one presented here can be complemented by intraspecific comparisons among *Bd* isolates to understand the evolutionary dynamics of genes hypothesized to play a role in *Bd* pathogenicity. However, robust hypothesis testing will require functional characterization of genes that may be important to *Bd*'s ability to infect frogs. *Bd* currently lacks a transformation system in which to study gene function, but heterologous expression systems could potentially be used to determine specific gene functions. Additionally, understanding expression patterns of candidate genes under different nutrient conditions and during different stages of host invasion are likely to yield important insights. Ultimately, identifying the molecular mechanisms of host-pathogen interactions will provide new avenues for mitigating the devastating effects of chytridiomycosis.

## Supporting Information

Figure S1 Alignments of M36, S41, and Asp domain protein sequences for *A. macrogynus* (AMAG), *B. dendrobatidis* (BATDEDRAFT), *H. polyrhiza* and *S. punctatus* (SPPG).
(PDF)

Figure S2 Maximum likelihood phylogenies of gene families containing (A) M36, (B) S41, and (C) Asp Pfam domains.
(PDF)

Table S1 Proteomes used in this study.
(DOC)

Table S2 M36. S41, and Asp protease gene family members for *A. macrogynus* (AMAG), *B. dendrobatidis* (BATDEDRAFT), *H. polyrhiza* (*Hp*), and *S. punctatus* (SPPG). 2A) Gene sequences. 2B) Delimitation of Pfam domain.
(XLSX)

Table S3 Restricted OrthoMCL analysis of 3 chytrid taxa, including 5 predicted protein sets.
(XLSX)

Table S4 Orthologous groups among 21 taxa as determined by OrthoMCL.
(XLSX)

Table S5 Gene family members of A) M36, B) S41, C) Asp protease and D) CRN-like gene family members for: *A. macrogynus*, *B. dendrobatidis*, *H. polyrhiza*, and *S. punctatus*.
(DOC)

Table S6 Table of duplication events for M36, S41 and Asp Pfam domain gene families in *A. macrogynus* (Am), *B. dendrobatidis* (Bd), *H. polyrhiza* (Hp) and *S. punctatus* (Sp).
(XLSX)
