# Supplementary material for: Genomic Transition to Pathogenicity in Chytrid Fungi
Source: PLoS Pathog. 2011 Nov 3;7(11):e1002338. doi: 10.1371/journal.ppat.1002338 (PMC3207900; doi:10.1371/journal.ppat.1002338)
Supplement: Figure S1 — Alignments of M36, S41, and Asp domain protein sequences for A. macrogynus (AMAG), B. dendrobatidis (BATDEDRAFT), H. polyrhiza and S. punctatus (SPPG). (PDF) [file ppat.1002338.s001.pdf]

Figure S1. Alignments of M36, S41, and ASP domain protein sequences for Allomyces macrogynus (AMAG), Batrachochytrium dendrobatidis (BATDEDRAFT), Homolaphlyctis polyrhiza (Hp) and Spizellomyces punctatus (SPPG).

Following are the aligned domain sequences for the three families we targeted.
There are two columns separated by tabs. The first column has gene names and
the second column contains the sequences.

```
###############
# M36
###############
>AMAG_00149T0	--------FASDPTEGDMALVGDPEDTAV-ASPQGWVG-TDG---------LTVGNNVVARQSPKAAALA--------
GHAPLVAKGASTTAFSATPALAFDFPADLRDGRPTDHLATSLTNAFYVVNLMHDLLYRFGFTETAGNFQTSNLGRGGLENDAVIVHVHDPSQRNNANFATPPDGVPPTLTAFLFDMSGTTTDPVVDDSILMHEVTHGL
VSRLTGGPANPNCLSTPESRAMNEGWADAFAAAFKARATY--ADA-T---IGSFNSGKPN-GLRRFPYSTSTTTNPLTYKDATP--NG-
EPHAVGEVWAQILWETLWAGAATHGFQANWRLTPGFR--------------------DGVNLTATALGGNVQVVQDWVDGMKLQAC-------NPTMRQARDAILLADQINFDGQNRCDLWRAFAKRGLGVNA-
>AMAG_07740T0	AKYSVLPVGTENPNKGPIAVVDSSTAVKTNASPQGWH--STGTTS----YQDTRGNNIQAQPNTATAS----------KNVRP--SGGSTLDFS-------AIKPDF-
TKAATSYTPAATVQLFYMLNSAHDLSYQYGFDEVSGNFQVNNFGKGGKGNDAVIANAQDRSGTNNANFATPPDGQQPTTRQYIFTQTTPTRDGVYDLTIPFHEFMHGITNRLTGGPSNVDCLYDGESGGMGEGWGDVF
GLVANVLTANS-VRT-GAFPMGAYVLGKAA-GIRTYPYSSDTTVCPNLYSFLGKTGYD-EVHRSGEIWATALLEVYFNLVEKLGYGDIFT--PSTT------------------------------
KGNTLFLKLVFDGNKLQPC-------NPSFVEARDAIIQADINLTGGANKCAIWKGFAKRGLGTGAK
>AMAG_07748T0	ASYEVYPIGVNDPDDGDRVRVKRPELVR--ASPDGWH--SD---------RTTIGNNVIAQENLSGKADALLKEGELSAAYRP--RSGHKLHFH--------YDIDF-
AKNPKSYLDASITQLFYMNNMLHDLFYAYGFTENAGNFQADNFGRGGVAGDPVVAFAQDGSGYNNANFATPPDGKNGKMRMYVWNTVVPNRDGDLENGIVIHEFGHGVSNRLTGGPHNSGCLAWGESGGMGEGWGDVW
ATIFRHRSADRARRDYGPWHMGKYANGGTT-GIRKYPYSADMDVNPSTYSFLNHQGYW-GVHAKGEVWASILLEVYWNLINDLGWTGDWKS-AAVD------------------------------
KGNTLFNQLVVDGLTMQPC-------RPTFLDARSAILQAEAVLTGGRHACAVWRGFAKRGLGVDAQ
>AMAG_09753T0	ASYEVYPLGVNDPDDGDRVRVKRPELAR--ASPDGWH--SD---------RTTIGNNVIAQENLSGKADVLMKEGELSSAYRP--RSGHKLHFH--------YDIDF-
AKNPKSYLDASITQLFYTNNALHDLFYAYGFTENAGNFQADNFGRGGVAGDPVIAFAQDGSGYNNANFATPPDGKNGKMRMYVWNTVVPNRDGDLENGIVIHEFGHGVSNRLTGGPHNSGCLAWGESGGMGEGWGDVW
ATIFRHRTADRAHRDYGPWHMGKYANGGST-GIRKYPYSPDVDVNPSTYSFLNHQGYW-GVHAKGEVWAAILLEVYWNLIDELGWTSDWKS-ASVD------------------------------
KGNTLFNQLIVDGLTMQPC-------RPTFLDARSAILQAEAVLTGGKHACAIWRGFAKRGLGVDAQ
>AMAG_09761T0	AKYSVLPVGIENPNKGSIAVVDSSTAVKTNASPQGWH--STGTTS----YTDTRGNNIVAQPNTATAS----------KNVRP--SGGSALDFS-------GIKPNF-
AVNAEQYTPAATTQLFYMLNMAHDLSYQYG--------------------------------
TNNANFATPPDGQQPTTRQYIFTQTSPTRDGVYDLTIPFHEFMHGITNRLTGGPSNTDCLYDGESGGMGEGWGDVFGLVANVLKANS-VRT-AAFPMGAYVLGKTA-GIRTYPYSSDMTVCPNKYSFLGKTGYD-
EVHRAGEIWASALLEVYFNLVEKLGYGDIFT--PSTT------------------------------KGNSLFLKLVFDGNKLQPC-------NPSFVEARDAIIQADVNLTGGANKCAIWKGFAKRGLGTGAK
>AMAG_10695T0	---------ASDPTEGEMALVGDPEDTAV-ASPQGARVRFRLPIASLPASYIAHGGYVSPHTEAAALA----------
GHAPLVAKGASTTAFSATPALAFDFPADLRDGRPTDHLATSLTNAFYVVNLMHDLLYRFGFTEGAGNFQASNLGRGGLENDAVIVHVHDPSQRNNANFATPPDGVAPTLTAFLFDMSGTVTDPVVDDSILMHEVTHGL
VSRLTGGPANPNCLSTPESRAMNEGWADAFAAAFKARATY------ADATIGAFNSGKPN-GLRRFAYSTSTTTNPLTYKDATPNG---
EPHAVGEVWAQILWETLWAGVASHGFQANWRLTPGFR--------------------DGTNLTANALGGNVQVVQDWVDGMKLQAC-------NPTMRQARDAILLADQINFDGQNGCDLWRAFAKRGLGVNA-
>AMAG_11091T0	AKYSVLPVGTENPDKGPITVVDSSTAVKSNASPQGWH--STGSQS----YTDTRGNNINAIANTKTAS----------KNIRP--DGGSALDFT-------KYKPDF-
TKAAETYTNAATAQLFYMLNTAHDLSYQYGFDEVSGNFQVSNFNKGGKGNDAVNAYSQDKSATDNADFGTPPDGQAPVTHQYIFTQTNPTRDGVYDLTIPFHEFMHGITNRLTGGPSNTDCLGDGESGGMGEGWGDIF
GLWANVLKADT-SRT-TKLPMGAYVMGQAN-GIRTYPYSSDLKVCPNLYSYLAKSQYN-EVHMAGEIWASLLLEVYWNLVDKYGYGDIWT--ADAS------------------------------
KGNTLFLRLVFDGLKLQPC-------NPTFINARDAILQADKNLTGGKNQCEIWKGFAKRGLGTKA-
>AMAG_11092T0	-RYTVLPIGTVSPKTGPLIDIDGGSVVKSNASPKGWH--ATNAES----WTYTRGENTFVLAP---------------NNVVPVGSGSDGLDFT------AWGKPDF-
SKNADQYTMQAAIQLFYEMNYAHDLSYQYGFDEVSGNFQVDNFGKGGAGNDAVLAYAQSADGHDNANFATPPDGEQPVTRQYIFLGTNPSTDGVYEPLIPLHEYMHGITNRLTGGPANVDCLSSVLSSGMSEGWSDVF
GLVVNVFKADS-DPN-ASYGVGVYVANNPN-GIRTYPYSNNMSVNPLKYSSLKT--MT-EPHAVGVVWAVILVEIYFNLVKKYGYGDLMA--ADFS------------------------------
KGNSKFLKIVFDALKLQPC-------NPHILHARNALLQADVNMTGGTSYCEIFLPFAKRGFGWSA-
>AMAG_11093T0	--------------------------------------------------TATSSKNVRL-------------------------SGGSALDFS-------AYKPDF-
TKAAETYTKAATVQLFYMLNTAHDLSYQYGFDEVSGNFQN-------------------------------------------------------------------------------------
EVHMIGEIWASMLLEVYFNLVCTTQYHD-----LTRFDAPAAR------ALNNAIPNAPSSLSTPTQ-------------------------VDKYGYGDIWA--
ADLS------------------------------KGNGLFFRLVLDGNKIQPC-------SPSLVDARDAILQADSNLTGGKNQCLIWKGFAKRGLGNSAQ
>AMAG_11126T0	-RYAVIPVGEQSPDMGPLVIVDSASVILANASPNGWH--DDSVVK----SKTTEGNNVTAQPNTPDGT----------KNGFP--DGGANLDFT-------
KYAPIFASSNPNDYAAGSAVQMFFLMNVAHDLCYQYGFDEASGNFQMDNYGKGGLGSDPIAAVVQDQNDVDTEYFMTPPDGQRPHVSAYLFDYTTPNRDSVFDLAIPHHEFFHGVSSRLTGGPANSDCLTPFEAASLA
EGWSDMFALAVNVLDKQTVTRN-TAAPFAPYVAGTPS-GLRTYPYTSDTQVNPSVYSYLGRADYQ-TVHKAGEVWASMLWEVYWNLVDKYGCGPIEQ--ADLA------------------------------
HGNTLWLQLIMDGLKIQNC-------NPSAVTARDAILTADLLLTGAANNCLIWSGFAKRGLGTAA-
>AMAG_11128T0	-RYAVIPVGEQSPDKGPLVIVDSAAVTLDNASPLGWH--ATSVST----YTTSKGNNVISKPNTNDDA----------KNVLP--DGSADLDFT-------
KYAPVFATADPNNYAAGSAVQLFDLMNVAHDLCYQYGFDELSGNFQMDNFGKGGLGTDAIDAMAQFAEASDVDTFVTPPDGQHPTA-
AYLFDYTTPNRDSVFDLAIPHHEFFHGVSSRLTGGPANSDCLTPFEAASLAEGWSDMFALAVNVLDKQTVTRN-TAAPFAPYVAGTPS-GLRTYPYTSDTQVNPSVYSYLGRADYQ-
TVHKAGEVWASMLWEVYWNLVDKYGCGPIEQ--ADLA------------------------------HGNTLWLQLIMDGLKIQNC-------NPSAVTARDAILTADLLLTGAANNCLIWSGFAKRGLGTAA-
>AMAG_11133T0	ASYSVLPVGIENPNKGSIQVVDSSTAVKSNASPQGWH--STGTTS----YQDTRGNNIQAQPNTATSS----------KNVRP--SGGSALDFT-------AYKPDF-
SKGADQYTPAATVQLFYMLNEAHDLSYQYGFDEASGNFQVNNFGKGGAGNDAVIANAQDRSGTNNANFATPPDGQQPTTNQYIFTETTPTRDGVYDLTIPFHEFMHGITNRLTGGPSNTDCLADGESGGMGEGWGDVF
GLWANVLKADT-KRG-TSLPMGAYVLGNSA-GIRTYPYSTDLSVCPNLYSYLGKSGYD-EVHMIGELWASMLLEVYFNLVDKSGFGDIWA--ADLT------------------------------
KGNGLFFRLVLDGNKLQPC-------SPTFINARDAILQADVNLTGGANKCEIWKGFAKRGLGSKA-
>AMAG_11353T0	AKYSVLPVGTENPNKGPITVVDSSTAVKTNASPQGWH--STGTTS----YTDTRGNNIQAQPNTATVL----------KNVRP--SGGSTLDFS-------AIKPDF-
TKAATSYTPAATVQLFYMLNSAHDCP------------
TVNNFGKGGKGNDAVIANSQDKSGTDNANFATPPDGQQPTTRQYIFTQTTPTRDGVYDLTIPFHEFMHGITNRLTGGPSNVDCLDDLESGGMGEGWGDVFGLVANVLKSSS-SRS-GSYPMGAYVLGSSA-
GIRTYPYSSDLSICPNTYAYLAKSDYQ-EVHQIGEIWASMLLEVYWNLVDAYGYGDIFA--ASTS------------------------------KGNSLFLRLVFDALKLQPC-------
NPSLVDARNAILQADVNLTGGANKCAIWKGFAKRGLGYSAK
>AMAG_14366T0	-RYPVILVGEQSPVKGPVVIVESAAVVLTNASPKGWH--DDT-AH----HTTTEGNNVHARPNTRDIN----------KNVAP--DGGANLDFT-------
QYSPVFATHDPIDYTAGSAVELFYLLNVAHDLTYQYGFDEQSGNFQVDNHGKGGQGNDAVLGLAQDGGRIDDSHFSAPPDGVEPFAAISIFTFTTPNRDSDFDLVIPHHEYFHGVSSRLTGGPANANCLRASEAKGMA
EGWSDMFGLMVNVLDKESVTRD-MAAPFAPYVAGVPS-GLRKFPYSTDMAMNPSTYSFLANNEYR-EPHRMGEVWASMLFEVYWNFVDRYGCGPMEQ--ANLT------------------------------
YGNALWLQLIIDGLKLQIC-------NPTFIQARDAVLLADLLLTRGANNCLIWRGFAKRGLGFAA-
>AMAG_14954T0	-RYPVIPVGQQSPDQGPVVIVDSAAVILASASPNGRH--DDTAKK----YTTTQGNNVKSRLNMDDAA----------TDVPT--DGGANLDFT-------
KFAPVIATANPNNYTAGSAVELFYLMNIAHK---------------------------------------------SPPSATRPQA-TFRWT-
VMAKEGCDFGLVIPHRELFLRRLEPAHGRARQPQLPGNAGGRGHGRGLVDMFALAVNVLGKQNVTRT-TATPFAPYVAGTPS-GLRTDPHTSDMQMNPSVYSLLGTEQYQ-
QVHKIGEVWASMLWDVYWNLVDMHGCGPIEM--ANLA------------------------------DGNALRLQLIMDGLKYQEC-------NPNFVMVRDAILTSAFVLTGGVDYCSIWRGFAKRGLGTTA-
>AMAG_15202T0	AKYSVLPVGTENPNKGPITIVDSSMAVKMNASPQGWH--STGSAS----YTDTHGNKIQAQPNTATAS----------KNVRP--SGSSTLDFS-------AIKPDF-
TKAVTSYTPTATVQLFYMLNSAHDLSYQYGFDEVSGNFQVNNFGKGGKGNDAVIANLQDKSGTDNANFVTPPDGQQPTTRQYIFTKTTPTRDGVYNLTIPYHEFMHGITNRLTGGLSNTDCLNDLESG----------
----------------------------------------DLSVCPNTYSSLSKSDYQ-
EVHQIGEIWASMLLKVYWYL--------------------------------------------------------------LQLC-------NPSLVNAHNAIIQADVNLKGGANKCAIWKGFAKRGLGYGAK
>AMAG_16399T0	------------------ANTKTAS-----------------------------------------------------KNIRP--DGGSALDFT-------KYKPDF-
TKAAETYTNAATAQLFYMLNTAHDLSYQYGFDEVSGNFQVSNFNKGGKGNDAVNAYSQDKSATDNADFGTPPDGQAPVTHQYIFTQTNPTRDGVYDLTIPFHEFMHGITNRLTGGPSNTDCLGDGESGGMGEGWGDIF
GLWANVLKADT-KRE-TKLPMGAYVMGQAN-GIRTYPYSSDLKVCPNLYSYLAKSQYN-EVHMAGEIWASLLLEVYWNLVDKYGYGDIWT--ADAS------------------------------
KGNTLFLRLVFDALKLQPC-------NPTFINARDAVLQADKNLTGGKNQCEIWKGFAKRGLGTKA-
>AMAG_16400T0	-RYTVLPMGTPNPQAGSFVDVDAGSVIKSNASPKGWH--ATNSES----WTYTRGENVFVLAP---------------NNVVPVGSGSDGLDFT------AWGRPDL-
SKNADQYTMQSAVQLFYDLNYAHDLAYQYGFDEVSGNFQVDNFGKGGAGNDALLAYNQDPSGSNNAYFVCPPDGQQPETHQFIFKSTNPTRDAVYEPVIPLHEFMHGISVRLTGGPANSDCLNQLLGGGMGEGWSDVF
GLILSVFNAGS-DPN-GSYAVGDYVTGNRK-GIRKYLYSSNMSINPLKYSSMKT--MT-NVHDAGEVWAVVLIEIYFNFVKKYGYGDLMA--ADLS------------------------------
KGNSKFLKTMFDALKLQPC-------NPNPLDGRNALLQADISMTGGTSYCEIFGPFAKRGFGWNAS
>AMAG_16401T0	AKYSVLPVGTENPNKGPISLVDSSTAVKSNASPKGWH--STGTTS----YQDTRGNNIQAQPNTATAS----------KNVRP--SGGSALDFS-------AYKPDF-
TKAAETYTKAATVQLFYMLNTAHDLSYQYGFDEVSGNFQVNNFGKGGRGNDAVIANAQDGSDTNNADFATPPDGQQPTTRQFIFTQTNPTRDGVYDLTIPFHEFMHGITNRLTGGPSNTDCLADGESGGMGEGWGDVF
GLWANVLKSST-KRG-TSLPMGSYVMGQAN-GIRTYPYSTDTNVCPNMYSFLGKSEYN-EVHMIGEIWASMLLEVYFNLVDKYGYGDIWA--ADLS------------------------------
KGNGLFFRLVLDGNKIQPC-------SPTLVDARDAILQADTNLTGGKNQCLIWKGFAKRGLGNSAQ
>AMAG_16405T0	--------------------------------------------------------------------------------------------------------------------------
--------------------------------------------------------------------------
RVAGGVFDLVIPHHEYFHGVSSRLTGGPANPNCLSTREAAGLSEGWSDMFALAVDVLDNPTVTRN-TATPFAPYVAGNPA-GLRTYPYTSDMAVNPSTYSFLALDAYQ-
EVHKAGEVWASMLWEVYWNLVDMYG------------------------------------------------------------C------------------------------------------------
>AMAG_16408T0	-RYPVIPVGEQSPDKGPIVIVDSAAVVMTNASPNGWH--DNTVRQ----FTTTQGNNADVRPNTNDDA----------QNVPP--DGGADLDFT-------
KFAPVFATADPNDFAAGSA---------------------
AAGNFQDDNYGKGGFGSDHVGAQVQVADGVNTEFFITPPDGQRPLVGAFLYTYTTPKRDSVFDLVIPHHEYFHGVSSRLTGGPANPNCLSTREAAGLSEGWSDMFALAVDVLDNPTVTRN-TATPFAPYVAGNPA-
GLRTYPYTSDMAVNPSTYSFLALDAYQ-EVHKAGEVWASMLWEVYWNLVDMYGCGPIEK--ADLT------------------------------NGNALWLQLIMDGLKIQNC-------
NPDFVTARNSILIADVLLTGGANSCLIWRGFAKRGLGSTA-
>AMAG_16410T0	--YPVIPVREQSPDRGPIVNVNSSKEVLINASPQGWH--KAGPTTS---FTDTRGNNVDARPNTADLA----------KNVRP--DGGPTRDFR-------PFAPDF-
```

TKNADQYTPGATVQLFYALNKAHDVSYKYGFDEASGNFQIDNYGKGGLSGDPIEALAQDLSGVNNANFATPPDGQSPTTRQYIFTYTNPTRDGVYDLVIPFHEFFHGVSNRLTGGPANTDCLPDGEPGGMGEGWSDIF
SMIAVVLDKPTVTRA-TPVPVGTYALGDPA-GVRKYPYSTDLAVNPSVYSFLSQAEYQ-EVHRIGEVWASMLFEIYWNLVDKYGCAPLEK--HNVR------------------------------
SGNALMLQLIMDGLKLQPC-------IPSFIDARTAILQADENLTGGANQCLIWKGFAKRGLGATA-
>AMAG_16411T0   --YPVIPVGVASPDAGPIVNVNENDVALTYASPQGWH--KAGMLA----FTDTRGNNVDKIPLTSDPA----------KNGRP--------DFR-------PFAPDF-
TKIAGQYTAGATVQLNYVLNSAHDPAYQHGFDEVSGNFQIENFGNGGFGDDAMKELVQHREGVINAFFSTPPDG--
----

>AMAG_16416T0   --YPVIPVGQQSPDNGPIVVVDSAAVILANASPNGWH--DD---------TTTRGNNVNTQPNTPDRN----------KNVPP--DGGADLDFT-------
RYAPVFATANPNDYAAGAAVQLFYLMNIAHDLTYQYGFNEISGNFQTNNYGKGGLDGDAITARAQDSMGVNAPYFMTPPDGSKPTATMQLFTLTTPNRDSDFDLVIPHHEFFHGVSSRLTGGPANPNCLSEFEAAGMG
EGWSDMFALAVNVLDKQSITRD-TAMPFAPYVAGIQV-GLRTYPYTSDMAVNPSTYSFLALDAYQ-EVHKAGEVWASMLWEVYWNLVDKYGCGPIEQ--ADLA------------------------------
HGNALWLQLIMDGLKIQEC-------NPNAVTARNAILTADLVLTGGANYCLIWNGFAKRGLGAAA-
>AMAG_16418T0   --
--------------------SGNFQFNSFGKGRLGGDQFAGSMQDYQESNDAQFSTPVEGQQPTTQFCVFMLTNPMRDSPFDMVIPFHEFW----------------------------
WSDIFALIAIHLDDPAITRN-TPVLVAMHMPGNAG-GICKYPYSTDLAINPSTYAFLSQAEYQ-EVHRIGEVCASMLFEIYWNLVDKYGCAPREK--HNVR------------------------------
SGNALMLQLIMDGLKLQVC-------RPTFIDARTAILQADQNLAGGQNQCLI---FAKHGLGFTA-
>AMAG_16424T0   --YPVIPVGVASPDAGPIVNVNEHDVALPNASPQGWH--RAGDLT----FTDTRGNNVDAIPNTADSS----------KNVRP--EGGEERDFR-------PFAPDF-
TKNADQYTPGATVQLFYALNACHDLSYQYKFD---
EADGMGEGWSDIFSLIAILLDDPNVTRN-TPMPVATYVAGSPA-GIRKYPYSTDKAINPSVYSFLAQDEYK-EPHNMGEVWASMLFEVYWNLVDKYGCGPIEQ--
RNLG------------------------------VGNALMLQLIMDGLKLQPC-------RPTFVDARNAILLADNNLTGGANQCLIWNGFAGRGLGIAA-
>AMAG_16427T0   -RYSVLPIGTENPDKGPIVVVDGSTAVQTNASPKGWH--ADESQT----FSDTRGNNILAQPNTKAG-----------NTKRP--DGGSALDFT-------AFKPDF-
SKPAEQYTDAATVQLFYMLNTAHDLSYQYGFDEVSGNFQTSNFNKGGKSNDPVQAYAQDRSGTDNANFATPPDGQKPTTRQFIFTQTKPTRDGVYDLTIPFHEYMHGITNRLTGGPSNTDCLWGGESGGMGEGWGDVF
GMVANTFKADS-DRN-AAWGLGAYVLGNTA-GIRTYPYSSNTQTCPNLYSFLAKPDY----------------------VDELGYGDLFS--ASTA------------------------------
KGNSLFLRILFDALKIQPC-------NPTFVEARDALISAEQQLTKGAYKCLIWKGFAKRGLGSKA-
>AMAG_16443T0   ASYSVLPVGIENPNKGSIQVVDSSTAVKSNASPQGWH--STGTTS----YQDTRGNNIQAQPNTATSS----------KNVRP--SGGSALDFT-------AYKPDF-
SKGADQYTPAATVQLFYMLNEAHDLSYQYGFDEASGNFQVNNFGKGGAGNDAVIANAQDRSGTNNANFATPPDGQQPTTNQYIFTETTPTRDGVYDLTIPFHEFMHGITNRLTGGPSNTDCLADGESGGMGEGWGDVF
GLWANVLKADT-KRG-TSLPMGAYVLGNSA-GIRTYPYSTDLSVCPNLYSYLGKSGYD-EVHMIGELWASMLLEVYFNLVDKSGFGDIWA--ADLT------------------------------
KGNGLFFRLVLDGNKLQPC-------SPTFINARDAILQADANLTGGANKCEIWKGFAKRGLGSKA-
>AMAG_16963T0   --YPVIPVGVASTDAGLIMNVNSADVVLAKASPQGWH--KAGVS-----FTDTRGNNVYAIPNTADPA----------KNVRP--DGGATLDFT-------PFAPDF-
TKSADRYTAGGTVQLFYALNHGARPVIP-----
ASGNFQMDNFGKGG--

>AMAG_17346T0   --
-----------CYQYGFDEAAGNFQMDNYGKGGLDNDAILAHSQDATGINNAVFMTPIDSQQPRAQMLLFDFTTPNRDSDSDLVIPHHEFFHGVSNRLTGGPANSDWLIQH------------
FAFAVNVLDNKTIARN-TATPFVPYVAGNPT-
GLRTFPFRSDLTVNPSTYS--
C--
>AMAG_17415T0   -RYPVIPVGEQSPEKGPLVIVDSAAVVLTNASPNGWH--AYTGQT----FTTTRGNNVIAHPNTPEEN----------DNGFP--DGGADLDFT-------
PFAPVFATANPKDYAAGSAVELFYLMNVAHDLCYQYGFDEASGNFQVDNYGKGGIGGDAILALSQDSTGKNNAVFMTPIDGEQPKVQMFLFDLTTPNRDSDFDLVIPHHEFFHGVSNRLTGGPANSDCLSTQEAAGMG
EGWSDMFALAINVLDNKSITRN-TATPLAAYVAGSPS-GLRTYPYTSDMTVNPSTYSLLGTNAYQ-EVHKIGEVWASMLWEVYWNLVDTYGCGPIEQ--ANLT------------------------------
DGNAMWLQLVMDGLKSQPC-------NPNMVTARNAILLADLLLTGGANNCLIWRGFAKRGLGTAA-
>BATDEDRAFT_11205   -TYKAIKVPDLSPNDG-FSDIKDPE-YKP-SSPNGW---TNG--------TVTEGNNAKTIDS---------------TKKPPVPATGVDGVFD------SKFNPAA-
NVTDPDNMQAAAVNLFYLANLMHDISYQYGFTEVAGNFQNDNFGKGGKGNDAVNINVMETSGSENALFLTPPDGQPGRMAMFRYTSTEPNRNPGLDSQIVVHEYGHGISSRLTGGPAATGCLLEGEARGMSEGWSDLF
AMIVTAKESH--KAD-TPIILGAYISDSSS-GVRTHPFTTDMKANPFTYSDIGN--NQ-ESYIVGEVWASILWEVYWNLVTKNGFSTNLHD-AKGK------------------------------
FGNIVTMQNMIGGFMLQPC-------KPTFLSARDAFIASDVAHYNGANKCEIWKGFAKRGLGVKA-
>BATDEDRAFT_12637   ATYKVIKLPGLTPDDG-FSNVKDPE-YKP-SSPNGW---TDG--------TTTQGNNVNALDL---------------RKDPPVPGTGVDGVFD------TKFNSTA-
DVADPDNMQAAAVNLFYVANLMHDISYQYGFTEAAGNFQTDNFGKGGEGNDAVNINVLDDSSTDNALFHTPPDGQPGVMLMFRYTTTEPNRSSGLDNQVPLHEYGHGISTRLTGGPATIECSFGREVVGMGEGWSDLF
AMIVTAKQSH--KAD-TPIGIAAYVKNKPN-GIRSHPYTTDMKVNPLTYADVKT--LK-SPHAIGVVWASMLWEIYWNLVTKNGFSTNLYD-AKGK------------------------------
FGNIITMQNMIGGMMLQPC-------HPTFLDARDAFIASDVARYNGANKCDIWKGFAKRGLGVKA-
>BATDEDRAFT_16271   --YKAIKLPGLTPDDG-FSDVKDPE-YKA-SSPNGW---TDG--------TTTEGNNVSALDL---------------RKEPAVPGTGVNGVFD------SKFNPTA-
DITDPDNMQATAVNLFYITNIMHDITYQYGFNEAAGNFQKDNFGKGGEGNDPVIISVLDTSDEENADFFSPADGQPGQMRMFRFAHFKPNRNPGLDNQVAMHEYGHGVSGRLTGGPAVVGCLRGAEAGGMGEGWSDLF
AMIVSAKESQ--KSD-TPIAIGTYIQGGPE-GIRSHPYTTDMKVNPLTYSTLKK--RL-EVHDAGEVWASMLWEVYWNLVTKNGFSTDIYD-AKSN------------------------------
SGNTIITMQNMIGGFMLQPC-------HPTLISARDAFIESDANRYKGANKCEIWKGFAKRGLGVKA-
>BATDEDRAFT_16613   -VYTVVPLGGKNPLQTPPTLVYNPE-NII-SSPFGWHDPSNGEGS----VPSTRGNNVVSRFQISE------------DNDTLTTVHSQSFYFT--------DQPDL-
TQDPSTYIKSVAVNAFYLANMFHDILYLYGFNEEAGNYQVSNRDLKGNEADPVIISVMDGDEENNAYFNSPSDGKPGILRLFVFTGITPNRHSGYDNSVVLHELTHGVSERLTGGPENSNCLQQLEPNGMGEGWSDAI
AIALEMKETD--TSA-DDKILGAYVKPKTRYGFRKYPYSTNTKLNPLVYSSING--VN-QTHYVGTVWGTILFEVYWSLVNQYGFEPDWTK-VTST------------------------------
KGNVVFLQLMVDGMKIQGC-------NPTFLSARSAILTAEKFRYNGVYRCSLLRGFARRGLGLDA-
>BATDEDRAFT_33981   -SYKVVQLPNSNPEQG-FSVVKDPE-FAG-SSPNGW---TDG--------SRTLGNNVEVTD----------------PSGRP--GAGQGGIFD------TNFNAQV-
DPRTPDNSQASTVNLFYVCNLVHDITYQYGFTEQNGNFQKDNFNKGGQGGDAVKINALNNRDTDNANFLTPPDGRSGIMNMFQFTDTNPRRDSGMDALVTIHEYMHGVSNRLTGGSATGQCLQNTESSGMGEGWSDWL
SLVMTAKQGD--KDV-DPVAVGTYVTNSQR-GIRSRPYSTDLGVNPLKNSDLAR--RN-EVHDIGEVWAAMLWEVYWNLVNKNGFSANLFD-AKQK------------------------------
AGNIIAIQVIIGGLMNQPC-------NPNFINAKNAILEADQQFYQGANRCEIIKGFSKRGLGPNA-
>BATDEDRAFT_36120   -SYKVIPVPKINADDG-FSLVKNPE-FKA-SSPNGW---SDG--------TSTSGNNINAVSF---------------ISGRAA-GTGNNGVFD------SNFNANA-
DPTDAENVQASAVNLFYIGNLMHDITYQYGFTEAAGNFQNDNFGKGGRDGDAVILTVQSQEKFNNANFFTPSDGQNGEMNMFIFTKTNPRRDGGLDNTIPLHEYGHGLSSRLTGGSATSQCLRTLESGGMGEGWSDIV
AMIVTAKQAN--TPT-TPIVLGDYVTNRPE-GIRSHPYSTDTNVNPLTYADLQT--RD-EVHDIGEVWATALWEVYWNLVTKSGFSANLHD-AKNA------------------------------
AGNIIALQNVIGGMMIQPC-------NPTFLDARDAIIASDAARYNGANKCEIWRGFSKRGMGPNA-
>BATDEDRAFT_36196   ASYDVYPLGVNDPDSGKRAIVFDTE-HPD-ASPYGWH--SKGTTRDA--YNTTIGNNVFAQDNPDGGNSWR-------NNYRP--SANDKMAFD--------FSIDE-
KKNPTSYLDAAITNLFYYNNIIHDLFYLYGFDEKAGNFQNDNYGNGGKGNDAVIANAQDGSGYNNANFATPPDGSQPRMRMYVWTVTKPYHDGDLESGIIIHEYSHGISIRLTGGPSNSDCLGWGESGGMGEGWGDFF
ATIVRMTPEN--TRN-DDFGMGSYASGRA--GIRKYAYSTSFKTNPSTYGYLKKPLYW-EVHAKGEVWAEILYEFYWALVDKHGFDGNWFD-NKLS--------------------
TEPGRHLTGLSGNKLALQLVVDGLKLQPC-------YPTFVSARDAILLADQTGTMGENQCVIWNAFAKRGLGLSA-
>BATDEDRAFT_34483   -SYRVIPLGGRDPLASKATLVQNPF-DKE-ASPLGWHDKNDSQGS----TSITSGNNVIAHDNSN-------------NSNNPSSGLTTANSFD------
FDFQFDDKNQDPSEYKDASVTNVFFLTNAYHDILFKYGFDEQSGNFQQSNIGGQGRGNDAVLAHVQDGSGLNNANFNTPPDGRTGVMRMFLFDTTNPKRDAAFDNGVVLHELTHGLSNRLTGGPSNSNCLQRTESGGM
GEGWSDLVALVLEMVESD--TAA-TPKPVGVYVTNDLQTGIRQFPYSTDLTINTHTYSKLST--TR-EVHDVGEVWATMLFEVYWNMVGKLGFTPNLKDDAKSG------------------------------
KGNAVFLQILVDGMKLQPC-------NPTFIQARDAIIQADKAANNGANLCEIQKGFAKRGLGINAQ
>BATDEDRAFT_35259   ----------------GTQLVQNPE-DLV-ASPLGWHNTDNGIGN----IASTFGNNVIAQENRKNQP----------NPILGLRPSVNTFSFQ--------
FSVNDIKQSPEEYADASIVNTFAMTNLYHDILYQYGFTEAAGNFQVNNFGRGGKGGDPVIANVQDGSGVNNANFATPPDGQAGIMRMFIFTSATPNRDGALENSVALHELTHGLSNRLTGGPANANCLSSTIALGLGE
GWSDVVALSLEVLPTD--TRN-DDRVMGAYVTNNPKRGIRLFPYSTNMNNNPHTYADVLR--NS-NRHYIGEIWASMLWELYWNLVEKLGFSTNIKDGAKSG------------------------------
KGNTVFLQLIVDGMKLQPC-------NPTFIQARNAIVRADRVANNGTNFCLIATAFAKRGLGQG--
>BATDEDRAFT_27765   -TYKAIQVPGFNPNDGGFSDIKDPE-YKP-SSPNGW---TNG--------TATQGNNVNAVDF---------------TKSPPAPGTGIDGVFD------TKFNPEA-
DPNDPDNMQAAAVNLFYIGNLMHDITYQYGFTEAAGNFQNDNFEKGGKGGDAISISILDSSSANDAVFLTPSDGQPGKMKMFRFTRVKPNRSGGLDNQVPLHEYGHGVSTRLTGGSLAPLCMSGHE------------
----------------TRVILGAYVINKPE-GIRSHPYTTDMKINPLTYGDLKT--RT-ELHEAGEVWATMLWEVYWNLVTKSGFSTNLYD-AKGK------------------------------
AGNIVAMQNVMGGLMHQPC-------SPSLVNARDAIIASDAAYYNGANKCEIWKGFAKRGLGVNA-
>BATDEDRAFT_28410   -TYKAIKVPDLSPNDG-FSDIKDPE-YKP-SSPNGW---TDG--------TVTQGNNIDALDY---------------TKKPPVPATGVDGVFD------TKFNPAA-
NVTDPDNIQAAAVNLFYITNLIHDITYQYGFNEAAGNFQKDNFGKGGKGGDAVNINVLDTSSAENALFHTPADGQPGIMLMFRFISTEPNRNGGLDNQVPLHEYGHGISTRLTGGSAAVECLFDGEAEGMGEGWSDIF
ATIITAKQSH--KAD-TPIGIAAYVDNTTN-GVRSHPYTTDMKVNPLTYADLQT--RE-KSHVMGEVWASILWEVYWNLVTKNGFSTNLYD-AKGK------------------------------
FGNIITMQNMIGGMMLQPC-------HPTFLDARDAFIASDVARYNGANKCEIWKGFAKRGLGVKA-
>BATDEDRAFT_85535   -SYNVIQVPNTSPDQG-FSIAKDPE-FAG-SSPNGW---TDG--------SRTSGNNANVADE---------------NNGQQ--GVGQGGIFN------TNFDAQS-
DPRTPENSQTSTVNLFYICNLMHDITYQYGFTEKNGNFQDDNFNKGGQGGDAVKVNALSTSGTNNANFLTPPDGSNGIMNMFRFTSTDPGRDGGLENPIPLHEYMHGVSSRLTGGSATAQCLQTTESGGMGEGWSDWI
ALAIIAKQGD--KDV-DPVAVGTFVTNSQK-GIRSHPYSTDTGVNPLKNSDLAR--RN-EVHDIGEVWTTMLWEVYWNLVNKNGFSANLFD-AKQK------------------------------
AGNIMAIQLVMGGLMLQPC-------NPTFIDARDAIIQADQQFYQGANRCEIIKGFSKRGLGPNAQ
>BATDEDRAFT_5304   -SYNVIGLPRANPTDG-FVVVNNPE-YKA-SSPNGW---TAG--------SITKGNNADVAR----------------RSNVLTPGVGRNGVFD------TNFDPTG-
QPTSATNIQASAVNLFYQANMMHDIMYQYGFTEAAGNFQVSNFGKSGRGNDAVQINVMSSNKINNAEFYTPADGQPGVMYMYRFTSSNPNRDSGLDNTISLHEYGHGVSTRLTGGASTVQCLRTTEARGMGEGWSDLF
AIVLTAKSTD--TST-SNIISGAYASNRRA-GIRMYPYTTNMNVNPLRYSNLRT--QS-EVHDIGEIWVTAMWEVYWNLVQKYGFSSNLHN-AKQQ------------------------------
AGNVIFLQNLIGGLMHQPC-------NPTFINARDAVLASDQAYYGGANVCEIWRGFAKRGFGVGA-
>BATDEDRAFT_5302   ASYKVIPIPKVDVNDG-FEMVKNPE-FNA-SSPQGW---TDG--------QETTGNNVLAADQ---------------TSGTP--SSGVNGVFD------SSFNPQE-
DPRTAANVKASAINLFYIGNIVHDISYQYGFTEAAGNFQKDNFGKGGEGNDVVVINVQSTAGTDDAKFYAPADGQPGEMDMFRFTTTTPNRDGGLDNVIPIHEYVHGISNRLTGGAATTQCLEALESGGMGEGWSDTI
AMYLTSKKTD--VAT-TPIVMGAYATGDAS-GIRSHPYTTDIKVNPLTYADLQT--RD-EVHDIGEVWAVMLWEVYWNLVAKNGFSTDLYN-AKGK------------------------------
AGNIIALQNVIGGMMIQPC-------NPTFLDARDAIIASDAARYNGANKCEIWRGFSKRGMGPNA-

```
>BATDEDRAFT_1614        -TYKAIQVPGFNPNDGGFSDIKDPE-YKP-SSPNGW---TNG--------TATQGNNVNAVDF---------------TKSPPAPGTGIDGVFD------TKFNPEA-
DPNDPDNMQAAAVNLFYIGNLMHDITYQYGFTEAAGNFQNDNFEKGGKGGDAISISILDSSSVNDAVFLTPSDGQPGKMKMFRFTRVKPNRSGGLDNQVPLHEYGHGVSTRLTGGSLAPLCMSGHETRGMSEGWSDIF
AMIVTAKESD--KAD-TPVILGAYVINKPE-GIRSHPYTTDMKINPLTYGDLKT--RT-ELHEAGEVWAAMLWEVYWNLVTKSGFSTNLYD-AKGK------------------------------
AGNIVAMQNVMGGLMHQPC-------SPSLVNARDAIIASDAAYYNGANKCEIWKGFAKRGLGVNA-
>BATDEDRAFT_1616        -TYKAIQVPGFNPNDGGFSDIKDPE-YKP-SSPNGW---TNG--------TATQGNNVNAVDF---------------TKSPPAPGTGIDGVFD------TKFNPEA-
DPNDPDNMQAAAVNLFYIGNLMHDITYQYGFTEAAGNFQNDNFEKGGKGGDAISISILDSSSVNDAVFLTPSDGQPGKMKMFRFTRVKPNRSGGLDNQVPLHEYGHGVSTRLTGGSLAPLCMSGHETRGMSEGWSDIF
AMIVTAKESD--KAD-TPVILGAYVINKPE-GIRSHPYTTDMKINPLTYGDLKT--RT-ELHEAGEVWAAMLWEVYWNLVTKSGFSTNLYD-AKGK------------------------------
AGNIVAMQNVMGGLMHQPC-------SPSLVNARDAIIASDAAYYNGANKCEIWKGFAKRGLGVNA-
>BATDEDRAFT_5299        -TYKAIPIPGLSPDDG-FSNIKDPE-YKP-SSPNGW---ING--------TLTRGNNAEAVNT---------------ANTPSTPGTGINGVFD------SKFNPAT-
DINDADNIQATAVNLFYLANLMHDITYQYGFNEAAGNFQTDNFGKGGKGSDAVVISVLDTARRENADFHAPPDGEPGRMRMFRFTSAKPNRNPGLDNQVVLHEYGHGISTRLTGGPTASLCMFSPETRGMGEGWSDIF
AMIVTAKQSH--KAD-TPTYFGRYSKNNNN-GMRSYPYTTDMQVNPLTYGYLKK--RG-EVHAVGEVWAAALWEIYWNLIAKNGFSTNLYD-AKSK------------------------------
AGNIITMQIMIGGMMLQPC-------NTNFIDARDAIVAADVAHYDGANKCEIWKGFAKRGLGPNA-
>BATDEDRAFT_5305        ATYKVIPLPGLSPDDG-FSSVKNPE-YKP-SSPNGW---IDK--------ASTEGNNANTINS---------------FKIPRGPETSVGGVFD------TKFNSTA-
DPNNPANMNAAAVNMFYFANLMHDITYQYGFNEAAGNFQNDNFGKGGRGEDAVTIEVLDVASANNAIFFAPVDGIPGKMRMFRYTSSKPHRNPGLDNQVVLHEYGHGISIRLTGGSSTDNCLSRAESNGMGEGWSDIF
AMIITAKQSH--KAD-TPIAFGSYAKNSPS-GLRSHPYTTDMKVNPLTYADLQT--RK-LAHDMGEVWAAMLWDIYWNLVTKSGFSTNLYN-AKGK------------------------------
FGNVITMQNMIGGMMLQPC-------NPTFIDARDAFIASDAVHYKGANKCEIWKGFAKRGLGVKA-
>BATDEDRAFT_5317        -TYKAIPIPGLSPDDG-FSNIKDPE-YKP-SSPNGW---TNG--------TLTRGNNAEAVNT---------------ANTPSTPGTGINGVFD------SKFNPAT-
DINDADNIQATAVNLFYLANLMHDITYQYGFNEAAGNFQTDNFGKGGKGSDAVVISVLDTARRENADFHAPPDGEPGRMRXXXXXXXXXXXXXXXXXXXXXXXXXXXXXXXXXXXXXASLCMFSPETRGMGEGWSDIF
AMIVTAKQSH--KAD-TPTYFGRYSKNNNN-GKRSFPYTTDMQVNPLTYGYLKK--RG-EVHAVGEVWAAALWEIYWNLIAKNGFSTNLYD-AKSK------------------------------
AGNIITMQIMIGGMMLQPC-------NTNFIDARDAIVAADVAHYDGANKCEIWKGFAKRGLGPNA-
>BATDEDRAFT_1501        -TYEAIPILNLSPGMNRFANITNPE-YSA-SSPNGW---TNG--------NFTQGNNVNAADF---------------SQSPPAPGTGINGMFA------TKFNDNV-
EPDDPDNIQAAAVNLFYLANLMHDITYQYGFTESAGNFQNDNFGKGGQGNDAVSISVLDTSSNNNAGFHTPADGQPGQMKMYRYTTSRPNHNPGLDNQVVVHEYGHGVSSRLTGGSATAACLTNSEALGMSEGWSDMF
AMITTARKTH--KAD-TSIPFARYASNQFD-GMRTHPYTTDMKINPLTYADLKN--RK-EVHAAGEVWAAMLWEVYWNLVTKSGFSTNLHD-ARGT------------------------------
SGNIVTMQNMIGGFMHQPC-------NPSLVDARDAFIASDVSHYKGANKCEIWKGFAKRGLGVKA-
>BATDEDRAFT_1480        -TYKAIPLPRLSPNDG-FSNVKDPE-YKA-SSPNGW---TDG--------TVTEGNNVNAEYY---------------GGELPVTGTGVKGVFD------TKFNPTA-
EPKDPDNIQAAAVNLFYLANLMHDITYQYGFNEAAGNFQKDNFGKGGEGNDPVNIDILNTSGTNNAIFFSPADGQPGTMRMFRFTYTKPNRNGAFDNQVPLHEYGHGVSGRLTGGAAASNCLVGGEAGGMNEGWSDMF
YMIVTATQSQ--NSK-TPIHFGSYAINNAN-GARSHPYTTDMKVNPLTYSDLKT--RQ-EVHAAGEVWVSMLWEVYWNLVTKNGFSTNLYD-AKGK------------------------------
SGNIIAMQNMIGGFMLQPC-------NPSLLDARDAYIASDVARYKGANKCEIWKGFAKRGLGVKA-
>BATDEDRAFT_1483        --YKAIKVPGLTPDDG-FTSIRNPE-YSA-SSPKGW---TDG--------TTTEGNNVSALDL---------------RSRTPIPGTGVDGVFD------SKFNPKT-
EFTEPENMQAIAVNLFYVTNLMHDITYQYGFNEAAGNFQNDNFGKGGKGNDGIIISLLDTSRKENADFHSPTDGQPGRMRMFRFAHFTPNRNPGLDNQVVMHEYGHGVSGRLTGGPVAVGCLFEGEPGGMGEGWSDIF
AMIVTAKQSH--RAN-TPIFLGTYISDSPR-GIRSHPYTTDMEANPLTYGDLRQ--RQ-EVHDVGEVWAAMLWEVYWNLVTKNGFSTDIYN-AKSN------------------------------
SGNTITMQNMIGGMMLQPC-------YPTLINARDAFIESDVNRYKGANKCEIWKGFAKRGLGVKA-
>BATDEDRAFT_1489        -TYKAIKVPDLSPDDG-FSDIKDPE-YKP-SSPNGW---TDG--------TVTQGNNIDALDY---------------TKKPPVPANGVDGVFD------TKFNPTA-
NVTDPDNIQAAAVNLFYITNLIHDITYQYGFTEAAGNFQKDNFGKGGKGGDAVNINVLDTSDKDNAIFLAPPDGQPGIMLMFRFISAKPNRNGGLDNQVPLHEYGHGISNRLTGGPEAVECLFDGEADGMGEGWSDIF
AIIITAKESH--KAD-TPIGIAAYVKNSPT-GIRSHPYTTDMKVNPLTYADIKT--RQ-KSHDMGVVWASMLWEVYWNLVTKNGFSTNLYD-AKGK------------------------------
FGNIITMQNMIGGMMLQPC-------DPTFLSARDAFIASDVARYNGANKCEIWKGFAKRGLGVKA-
>BATDEDRAFT_1469        -TYKAIKLPDLNPNDG-FSDIKDPE-YKP-SSPNGW---TNG--------TVTEGNNAKTVDY---------------TKKPPVPANGVDGVFN------TKFNSTA-
DVTDPDNMQAAAVNLFYVANLMHDITYQYGFNEAAGNFQTDNFGKGGEGNDAVTINVLDASSTDNALFHVPADGQPGIMRMFRFTPAKPNRNGGLDNQVPLHEYGHGISTRLTGGPAAVECLFKGEAGGMGEGWSDIF
ATIITAKESH--KAD-TPIVIAAYAKNTPI-GIRTHPYTTDMKVNPLTYADVKT--LK-SPHAIGVVWASMLWEIYWNLVTKNGFSTNLYD-AKGK------------------------------
AGNIITMQNMIGGMMLQPC-------HPTFLDARDAFIASDVARYNGANKCEIWKGFAKRGLGVKA-
>BATDEDRAFT_1593        --YKAISPDNINPNEGGFTNIKDPE-YKP-SSPNGW---TDG--------TTTQGNNVNALDL---------------RKDPPVPGTGVDGVFN------TKFNSTA-
DVTDPDNMQAASVNLFYVANLMHDITYQYGFNEAAGNFQNDNFGKGGEGNDAVTINVLDDSSTGNAFFQTPADGQPGLMRMFRFISAEPNRNSGLDTQVVLHEYGHGISNRLTGGPATIGCLFKGEPGGMNEGWSDIF
AIIITAKESH--KAD-TPIVMGTYIANSPK-GIRSHPYTTDMKVNPLTYADVKT--LQ-SSHDMGVVWASMLWEVYWNLVTKNGFSTNLYD-AKGK------------------------------
AGNIITMQNMIGGMMLQPC-------HPTFLDARDAFIASDVARYNGANKCEIWKGFAKRGLGVKA-
>BATDEDRAFT_14272       ------------------------------------------------------------------------------------------------------------------
----------------------------------------------------------------------------------------------
LHEYGHGVSTRLTGGSLAPLCMSGHEIRGMSKGWSDIFAMIVTAKESD--KAD-TPVILGAYVINKLK-GIRSHPYTTDMKINPLTYGDLKT--RT-ELHEAGKVWATMLWEVYWNLVTKSGFSTNLYD-
AKGK------------------------------AGNIVAMQNVMGGLMHQPCKYPSGYSNTSLVNARDAIIASDAAYYNGANKCEIWKGFAKRGLGVNA-
>BATDEDRAFT_11777       -SYRVIPLGGRDPKTSSTALIRNPV-DST-ASPFGWHDLNNGDGS----VSITRGNNVVAHDNSN-------------RRDNPNTGLVTAQNFD------
FDFKFDDNQQEPGQYKDASITNVFFLSNSYHDILFKYGFDEQAGNFQNINAGGSGRGNDGIIAHVQDGERFNNANFFTPPDGRPGIMRMFLFNKSRPRRDSGLDNGIVLHELTHGLSTRLTGGPSNSNCLQFTEAGGM
GEGWSDIVALVLEMLPTD--TPD-TTKVVGGYSTNDPRAGVRFNPYSTSTSVNPSTYARIRR--NT-EVHAIGEIWASMLYEVYWNLVEKLGFSTNIKDDAKSG------------------------------
KGNTLFLQLIVDGMKLQPC-------NPTLIQARDAIIQADKVANGGSNFCEITRGFAKRGMGIRA-
>BATDEDRAFT_1639        -TYKAIKVPGLSPKDGGFTNIKDPE-YKP-SSPNGW---TDG--------TTTEGNNADVLDL---------------RPRTPVPGAGVDGVFD------TSFDPKT-
DIDNKENLQATAVNLFYLANLMHDITYQYGFNEAAGNFQKDNFGKGGEGNDAVSISVLDDSDDGNADFYTPADGQPGEMRMFRFTSAKPNRSPGLDNQVPLHEYGHGISTRLTGGPAAVGCLLKGQADGMGEGWSDIF
AMIVTAKKTH--KAT-TPISFAAYSENSPS-GFRSHPYTTDMKVNPLTYADIKE--RE-EVHDMGELWAAMLWEVYWNLVTKNGFSTNLYD-AKGK------------------------------
AGNIITMQNMIGGMMLQPC-------KPTFLSARDAFIASDVARYKGANKCEIWKGFAKRGLGVKA-
>BATDEDRAFT_5372        ------------------------------------------------------------------------------------------------------------------
----------------------------------------------------------------------------------------------
LHEYGHGVSTRLTGGSLAPLCMSGHETRGMSEGWSDIFAMIVTAKESD--KAD-TPVILGAYVINKPE-GIRSHPYTTDMKINPLTYGDLKT--RT-ELHEAGEVWAAMLWEVYWNLVTKSGFSTNLYD-
AKGK------------------------------AGNIVAMQNVMGGLMHQPC-------SPSLVNARDAIIASDAAYYNGANKCEIWKGFAKRGLGVNA-
>SPPG_01665T0   -SYTVADFRGADPTKQ-IITVKDPA-NAV-ASPKGWH--NDGTTA----FTETRGNNAFAYNSS-------------GGSKVTAKGGAQNNYN------TKYDVNA-
NANTASNKQAAIANVFYVANSMHDLFYQYGFNEAAGNFQVSNNGKGGRGNDAVSISAQDPNGTNNANFATPPDGQTPQMNMYVFTSTKPTRDGDLENDIPIHEYTHGISNRLTGGPANSNCLRTTEAGGMGEGWSDTA
ALFVQRRATH--TRD-TDLVMGDWVTGNPR-GIRTQPYSTSLQRNTLKYSTVGR--LN-EVHAIGEVWATIWNEVYWNLVDQSGFSDDIFN-ANQE------------------------------
KGNIVAFQLFVDSLALQPC-------NPTMIAARDAILQADENRYDGKYKCAIWKGFAKRGMGANA-
>SPPG_02641T0   ATYHVFPLGINDPESGDRVLVRDPA-HPL-ASPMGWH--NQGNGGQ---HTVTIGNNVYAHENLEGTSSWV-------DNHRP--DGTVSLTFD--------FPIDF-
KKQPSTYLDAAIVNLFYWNNAIHDLLYVYGFDEKAGNFQHDNIGRGGKGGDAVIANAQDGSGYNNANFATPPDGRQPRMRMYVWDVTRPFRDGDLEGGIVMHEYCHGLSIRLTGGPMNVGCLGWGESGGMGEGWGDFF
ATVTRTTPNS--TRH-DNFGMGEYANGG-Y-GIRKYKYSTDKSINPSTYGFITRPGYW-GVHAKGEVWAEILYEVFWNLVDKHGFDPDWFN-TGYKVSPSETHYRDFRTGQLQPRRKDPKQNTKG--
GNILALQLVVDGMKLQPC-------YPTFVDARDAILQADELNNDGENLCTLWEAFAKRGLGVDAK
>SPPG_04344T0   -KYRALPFGSKNVQDTPPVLIANPF-NPA-SSPLGWHDVGDGAGS----LPTTVGNNVVAQENRKN------------AKNPLNNKRPIALDFN------FDFPVDL-
SKEPTTYTDASVTNMFFLSNFFHDTMYKYGFTEAAGNFQATNLGKGGKERDPVVANAQDGSGVNNANFATPPDGQVGVMRMFIFDTSRPSRDGTLDNDVPIHELTHGVSMRLTGGPANSNCLDTLMAGGMGEGWSDFL
GVSFGMTAKD--NRN-TDKPVGQYVTGSPK-GVRGIPYSTNQKTNSLTYETLNDPKFA-AVHQVGTVWNTMLFEAYWNMVDAKGFTENFQDGPQSG------------------------------
KGNALMIQYVVDGLKLQPC-------NPDMIQARDAIIAAEKALTKGDNMCLLMTAFAKRGLGATA-
>Hp_gm_ctg00587-0.7     -SYRAVAVGNRDPRTSPPSLIQNPF-DQG-ASPLGWHDTGNGKGS----QPVTVGNNVVARDNSKNDN----------NPNSGFITAKTAFDFD--------
FAMDDTTQQPPQYKPASITQAFFIANAYHDILSKYGFTEAAGNFQVNNLGRGGVGNDPVIANVQDGSGTNNANFASPPDGQAGFMRMFIFTSTNPDRDGAFENAVILHELTHGLSNRLTGGPANANCLQTTESGGLGE
GWSDSVALTLEMTAAD--TPN-TDKAVGQYVSGNQQRGVRQFPYSTSLQRNPHTLADAVK--NP-EVHAIGEVWASMLFEVYWNLVGKLGFEPNLKDNARSG------------------------------
KGNTVFLQAVVDGMKLQPC-------NPTFVQARDAILKADVVRNKGANQCEIIKGFAKRGLGIN--
>Hp_gm_asm134-0.164     ASYTVYPLGTNDPADGPRVKLVNPA-HHD-ASPYGWN--SKGKKGHISYTNVTVGNNVYAQENLDGGSQWR-------ANYRP--TASDDLTFD--------FAVDL-
KKQPSTYLDASI-------------------------
FQEENYGRGGKGGDAVIANAQDGSGYNNANFATPPDGEQPRMRMYVWSASQPYRDGDLEGGIITHEYLHGVSTRLTGGADNVDCLGWGEAGGMGEGWGDYFATILRFTPET--TRN-DNFNMGGWSANRTA-
GIRKHIYSTSLKTNPSTYGFTKKPDYW-EVHAKGEVWAAILYEVVWNLIDKHGFEPDYFN-VNYG--------------------KD--NNLVGLKGNILAFQLVMDGLKLQPC-------
YPTFVDARDAILLADQIGTRGANQCEIWTAFAKRGLGIGA-
>Hp_gm_asm210-2.29      ASYRVVPITTNDVSKG-TTLLHNPE-YQG-SSPLGWL--SDGATS----YTITKGNNADVNRPAL-------------ARSPTIYGTSQNNVFD------SSFNAGQ-
QPDTAVNIQASAVNLFYVANIMHDISYQYGFTEATGNFQTNNFGKGGNGNDAVIVRVQDPSDTDNAMFLTPADGQPGIMQMFIFVDTQPNRDGGLEDPIPAHEYTHGISNRLTGGSATSQCLQTDEATGMGEGWSDTV
ALIVTARPGM--NRN-TPIYIGSYATNSPT-GIRTKPYTTDKSLNPLMFSTLQSSTYNEEPHNMGEIWVAMLWEVYWDLVDAYGFSTDLYN-AKQN------------------------------
AGNVKMLQLIVGGFMHQPC-------NPTFTTARDAIILADALYYGGQNKCLIWKAFARRGLGVAA-
>Hp_snap_msk_ctg00587-0.5       ASYRVVPITTNDVSKG-TTLLHNPE-YQG-SSPLGWL--SDGATS----YTITKGNNADVNRPAL-------------ARSPTIYGTSQNNVFD------
SSFNAGQ-
QPDTAVNIQASAVNLFYVANIMHDISYQYGFTEATGNFQTNNFGKGGNGNDAVIVRVQDPSDTDNAMFLTPADGQPGIMQMFIFVDTQPNRDGGLEDPIPAHEYTHGISNRLTGGSATSQCLQTDEATGMGEGWSDTV
ALIVTARPGM--NRN-TPIYIGSYATNSPT-GIRTKPYTTDKSLNPLMFSTLQSSTYNEEPHNMGEIWVAMLWEVYWDLVDAYGFSTDLYN-AKQN------------------------------
AGNVKMLQLIVGGFMHQPC-------NPTFTTARDAIILADALYYGGQNKCLIWKAFARRGLGVAA-
>Hp_mkr_asm210-snap-2.3 ASYTVYPLGTNDPADGPRVKLVNPA-HHD-ASPYGWN--SKGKKGHISYTNVTVGNNVYAQENLDGGSQWR-------ANYRP--TASDDLTFD--------FAVDL-
KKQPSTYLDASI-------------------------
FQEENYGRGGKGGDAVIANAQDGSGYNNANFATPPDGEQPRMRMYVWSASQPYRDGDLEGGIITHEYLHGVSTRLTGGADNVDCLGWGEAGGMGEGWGDYFATILRFTPET--TRN-DNFNMGGWSANRTA-
GIRKHIYSTSLKTNPSTYGFTKKPDYW-EVHAKGEVWAAILYEVVWNLIDKHGFEPDYFN-VNYG--------------------KD--NNLVGLKGNILAFQLVMDGLKLQPC-------
YPTFVDARDAILLADQV------------------------

###############
```

```
# S41
###############
>BATDEDRAFT_37569       NMGVIKLDDFEPTDPITDAESIDPAIRIIRSLLSKELKDT-KSVVFEIRSNPGGYITFAD-MLPQLF---KPD----------FEPFGARYLMNNVT-----HNIF---
VEGKDPS--DIWAQVWSETP----AG-SRYTKIGDFDTAEATNTYGQAYVRP----
MGVFTDGNCFSACELFSANVQNYDAGTIFGEDGNTGGGGANILDVDPALMLMDPFDFKQFPFTNELTWKPTSGKYMNRLSVGIRQSVRNGKYNGQLIEDLGIKTDIVV
>BATDEDRAFT_35365       NLGIIKLEDFSPTNLKTGALADVEAVREIHKLLSTVLKDT-NAVVFDIRANGGGSGEFAN-AILQLF---KPD----------FQPMPMRYLMNSVA-----YNTL---
VNGTYFF--DTSNPAWYKTP----PG-SKYSIDHDRTSYNQSNLYGQAYLKP----
VGAFTNAMCYSACEVFSAALQSFEIGYVFGEDGTTGGGGADMFGLDSELLVFNHV-----------------------------------------------------
>BATDEDRAFT_22176       NLGIIKLDDFMPKNLKTDAPADVEAVREIHKLLSTVLKNT-NAVVFDIRANGGGSGEFAN-AIPQLF---KPD----------FQPTQVRYLMNSVT-----YNTL---
VNGTRFF--DTSGPAWYKTP----PG-SKYSIDHDRTSYNQSNMYGQAYLKP----
VGAFTNAMCYSACEEFSAALQSSETGYVFGEDGTTGGGGADMFLLDSRLLVFNPVD----------------------------------------------------
>BATDEDRAFT_26098       NLGIIKLEDFSPRNLKTGARANAEAVREIHRLLSTVLKDT-NAILFDIRGNPGGGGQFSS-AIPQLF---KPD----------FQPVLVRYLMSSVA-----YNTL---
VNGSQSF--DTSGPAWYNTP----PG-SKYSVDHVKNSFNQANMYGQAYLKP----
VGAFTNAMCYSACEVFSAALQSSETGHIFGEDGTTGGGGADMFGLDP-------------------------------------------------------------
>BATDEDRAFT_27937       NLGIIRLDDFSPTNLKTDAPADTEAVREIHKLLSTVLKNT-NAIIFDLRANSGGSGEFAN-AIPQLF---KPD----------FQPMPMRYLMSSVA-----YNTL---
VNGTYFF--DTSNPAWYKTP----PG-SKYSIDHDRTSYNQSNLYGQAYLKP----
VGIFTDAVCYSACDLFSASIQSSETGHVFGEDGTTGGGGADMFSLDPHFLLF--------------------------------------------------------
>BATDEDRAFT_28623       NMGIIKLTSFECTSRTTNLMSVLEAIREVRGLLKTVLKDT-NSILIDVREGGGGSMRFAN-AIPQLF---KSN----------IEPFKVKYLRNKVT-----YNMF---
VKSPIDL--RSSRNAWSKTS----PDDSKYSLFHDFTDPENIITCSKVYTKP----MGVFTSGDCVSACEIVAGTVQSYDIGTVFGEDITTTGAGANVWNLDPDLILYDPTDFKPMPFKKELTSH-
NKEAFYNQMTV---------------------------
>BATDEDRAFT_28625       NMGIIKLTSFECTSRTTNTQKVSEGVLEVRRLLKTVLKDT-NSILIDVREGDGGSMDFAN-AIPQLF---KSN----------IEPFKVKYLRNKVT-----YNMF---
VKSPIDL--RSSRNAWSKTS----PDDSKYSLFHDFTDPENIITCSKVYTKP----MGVFTSGNCASACEIVAGTVQSYDIGTVFGEDITTAGAGAAVWNLDLQLTFYDPTDFKPMPFKKELTSH-
NKEAFYNQ------------------------------
>BATDEDRAFT_23310       NLGIIKLDSFMPTNLKTGAPADTEAVREIHKLLSTVLKDT-NAVIFDLRANSGGSGDLAN-AIPQLF---KPD----------FQPMPMRYLMSSVA-----YNTL---
VNGTYFV--DTSGPAWYKTP----PG-SKYSVNCNRTSYNQSNLYGQAYLKP----
VGIFTDAVCYSACDLFSASIQSSETGHVFGEDGTTGGGGADMSSLDPE------------------------------------------------------------
>BATDEDRAFT_23534       NMGIIKLTSFRCTSRTTNTRDIPEAIREVKRLLTTVLKKT-NSILIDVREGGGGSMSFAN-AIPQLF---KSK----------LEPFQVKYLMNDVT-----YNIF---
VHPPIDL--KSSRDAWFKTP----AK-SKYSLFHDFLDPRASNIYDQVYTKP----
VGVFTSGNCMSACEIMTGTMQSFKIGTVFGEDVTTAGSGANVWSLDPELIDCD-------------------------------------------------------
>BATDEDRAFT_23544       NMGIIKLTSFKCTSRTTNTRDIPEGIREVKRLLTTVLKKT-NSILIDVREGGGGSMSFAN-AIPQLF---KSK----------LEPFQVKYLMNDVT-----YNMF---
VHPPIDL--KSSRDAWFKTP----AK-SQYSVFHDFLDPRASNIYDQVYTKP----VGVFTSGDCMSACEIMTGTMQSFKIGTVFGEDVTTAGSGANVWSLDPELITHDPIDFKPMPFKKELTSD-
TKEIFYNK------------------------------
>BATDEDRAFT_23754       NLGIIRLDDFMPTNLKTGAPADMEAVREIHKLLSTVLKDT-NAVIFDLRASGGGSGEFAN-AIPQLF---KPD----------FQPTPMRYLMSSIA-----YNTL---
VNGTYFF--DTSNPAWYKTP----PG-SKYSIDHDRTSYNQSNLYGQAYLKP----
VGIFTDAICYSACDLFSASFQSSETGHVFGEDGTTGGGGADMFSLDPELLLFNPID----------------------------------------------------
>BATDEDRAFT_28775       NLGIIRLDDFVPKNLKTDAPADTEAVREIHKLLSTVLKDT-NAVIFDLRANSGGSGDFAN-SIPQLF---KPD----------FQPMLIRYLMNSVT-----YNTL---
VNGTYFF--DNSGPAWYNTP----PG-SKYSVDHVDTPFDRANMYGQAYLKP----VGIFTDAICYSACDLFSASIQSSETGHVFGEDGTTGGGGSDTFSLDSYLLLFNHIDFQPMPYAKELTDN-
TTGQSYTN------------------------------
>BATDEDRAFT_24156       NLGIIRLEDFAPTNLKTGAPANMEAVCEIHRLLSTVLKDT-DAVVFDIRANEGGSGEFAN-AIPQLF---KPD----------FQPMPMRYLMSSVA-----YNTL---
VNGTFFV--DTSGPAWYKTP----PG-SKYSVNFDKTSFNQSNLYGQAYLKP----
VGIFTDAVCYSACDLFSASIQSSETGYVFGEDGTTGGGGADTFSLDSQLLLFNPVDF---------------------------------------------------
>BATDEDRAFT_24207       NLGVIKLSHFLPFHFEFGRLGVEEAIFEIHNLLSTVLKDT-NAIVFDVRGNTGGMSEFAN-GILQFF---KPD----------FQPMQSRFLMNKLT-----YSTL---
VNGTRSF--DTAIDAWNKTP----PG-SKYSIPYNISSFDKANLYGQAYLKP----VAALTDATCRSECDLFVASLQSSETGYVFGEDGTTGGAGADVFKLDPLLMFFDPTDFKKMPYFDYLSTG-
N-------------------------------------
>BATDEDRAFT_24985       NMGIIKLESFRSTLRATNVPGIPEGVLEVRKLLTTVLKDT-KSVLIDVREGGGGAMGFSS-GIPQLF---KAD----------FQPFKATYLMNNVT-----YNMF---
VNPPIDL--ETSRDAWSKTP----PG-SKYSVLHDLIDPKSANDYGQVYTKP----
MGVFTTGDCFSACEVMTGSVQSSGVGTVFGEDSTTGGGGANVWILDPELINF--------------------------------------------------------
>BATDEDRAFT_25222       NLAVVRLDTFMPSVNHSTLTPVADTLEVLRYLLTHQLKYT-RALVLDIRSNSGGLYDFAS-QIPQFF---KPD----------HHPLQMQYLVNNAT-----YSLF---
VNHTSVA--SPFYNAWLSTP----PG-SNYTTMIDIVPMQSVNLIGQMYLRP----MGVFNNGGCYSACEMFTASIQDHNIGTVFGEDGQTSGSGSLSFDTQ-
DLVSHNPVDFTPAPYTSNLTSPDGQTKA---------------------------------
>BATDEDRAFT_25462       NMGIIRLASFRCTSRTTNTQNTLEAILEVERLLKTVLKDT-NSILIDVREGAGGCPIFAN-VILQFF---KSD----------IEPLKVKYLINKVT-----SNMF---
SNSHEGL--ETSCDAWSKTP----AG-SKYSVLHYIVDPKELGTYNPIYDKP----MGVFTNGNCVSACEVMTESVQSFGIGTVFGEDVTTAGAGANVWDLDPELIFYDPTDFKPMPFKKELTSD-
TKEIFY--------------------------------
>BATDEDRAFT_25463       NMGIIRLASFSCTSRTTNTQNTLEAILEVTRLLKTVLKNT-NSILIDVREGGGGCPIFAN-AIPQLF---KSK----------LEPFQVKYLINKVT-----SNMF---
ANSPKGL--ETSCDAWSKTP----AG-SKYSVLHYIVDPKELGTYNPIYDKP----MGVFTNGNCVSACEVMTESVQSFGIGTVFGEDVTTAGAGANVWDLDPELIFYDPTDFKPMPFKKELTSD-
TKEIFY--------------------------------
>BATDEDRAFT_25559a      NMGIIRLASFSCTSRTTNMQNTLEAILEVTRLLKTVLKNT-NSILIDVREGGGGCPIFAN-AIPQLF---KSK----------LEPFQVKYLINKVT-----SNMF---
ANSPEEL--KSSRNAWSKTP----AG-SKYSVLHYIMDPKELGTYNPIYDKP----
MGVFTSGNCVSACEVMTESVQSFGIGTVFGEDVTTAGAGANGWDLDPELIFYDPTDFKPMPFKKELTSD---------------------------------------
>BATDEDRAFT_25559       NMGIIRLASFSCTSRTTNMQNTLEAILEVTRLLKTVLKNT-NSILIDVREGGGGCPIFAN-AIPQLF---KSK----------LEPFQVKYLINKVT-----SNMF---
ANSPEEL--KSSRNAWSKTP----AG-SKYSVLHYIMDPKELGTYNPIYDKP----
MGVFTSGNCVSACEVMTESVQSFGIGTVFGEDVTTAGAGANGWDLDPELIFYDPTDFKPM------------------------------------------------
>BATDEDRAFT_27246       NLGIIRLDDFMPTNLKTGAPADMEAVREIHKLLSTVLKNT-NAVIFDLRANGGGSGEFAN-AIPQLF---KPD----------FQPTPMRYLMSSIA-----YNTL---
VNGTYFF--DTSNPAWYKTP----PG-SKYSIDHDRTSYNQSNLYGQAYLKP----
VGIFTDAICYSACDLFSASFQSSETGYVFGEDGTTGGGGADMFSLDPELLLFNPI-----------------------------------------------------
>BATDEDRAFT_26287       NMGIIKLTSFGCTSRTTNLVSVFEGIRVVKRLLKTVLKNT-NSILIDVREGGGGSVDFAN-AIPQFF---KSN----------IKPFEVKYLISDVT-----YNIF---
VNMPIDL--KSSRNAWFKTP----PK-SKYSILHDFTDPKMISTYDQVYDKP----MGVFTSGNCASACEIMTGTVQSHDIGTVFGEDVTTAGAGANIWSLDPELITHDPTDFKPVPFKKELTSS-
TKETFYNKMTVGNRAFVRNDKHKEKWIEDTGIVSDIVI
>BATDEDRAFT_85649       NLGIIRLDDFIPKDLKTGARVDIEAVREIHKLLSTVLKDT-NAVIFDLRANPGGSGDFAN-AIPQLF---KPD----------FQPMPMRYLMSSVA-----YNTL---
VNGTYFF--DTSGPAWYKTP----PG-SKYSIDHDRNSFNQANMYGQAYLKP----VGAFTDAVCYSACDLFSTSIQSSETGYVLGEDGTTGGGGADVFPLNPTLLLFNRVDFKPMPYAKELTDK-
TTGQSYT-------------------------------
>BATDEDRAFT_86001       NLGIIRLDDFMPTNLKTGAPANMEAVREIHRLLSTVLKNT-NSVLIDLRANGGGNGNFAN-SIPQLF---KPD----------FQPMTMRYLMSSVA-----YNTL---
VNGTQSF--DTSGPAWYKTP----PG-SKYSIDYVDTPFDRANMYGQAYLKP----
VGIFTDAVCYSACDLFSASFQSSETGHVFGEDGTTGGGGADMLSLDSQLLFL--------------------------------------------------------
>BATDEDRAFT_86231       NMGVIKLDNFSPKLASTGTDASSVATSFVRRLLTHELKDT-SSIVFELRGNPGGSILFAN-SLPQLF---KPD----------FDSFGARYLKNNVT-----YNIF---
VKGKSAR--DTWVKVWNESP----AD-ARYTNIGPFNSFEAANSYGQVYTKP----
MGVFTDGNCFSACDLFSANIQGHEAGTVFGEDGLTGAGGANILDVDPALVIYSPTDFQRFPFSLELTNPATGSTYANMLSVGIRQSVRNGKYDGQLIEDTGIKSDIVV
>BATDEDRAFT_86314       NLGIIRLDDFVPKNLKTGAQADMEGVREIHKLLSTVLKDT-NAVLIDLRKNLGGNGNFAN-AIPQLF---KPD----------FQPMLIRYLMSSVA-----YNTL---
VNGTSFF--DTSGPAWYKTP----PG-SKYSIDHDRNSFDQSNMYGQAYLKP----
VGAFTDAICYSACDLFSASFQSSETGHVFGEDGTTGGGGSDIFSLDSTLLALNPVDF---------------------------------------------------
>BATDEDRAFT_87928       NMGIIKLASFECTSRTTNTQKVSEGVLEVERLLKTVLKDT-NSILIDVREGGGGSLDFAH-EILQLF---KSD----------IEPFKVKYLRNKVT-----FNIF---
VESPIDL--RSSRNAWLKTP----PDDSEYSLFHDFTDPEDSGTYNQIYDKP----
MGVFTSGNCMSACEAMTGTVQSYNIGTVFGEDATTAGSGAIVWDLDPDLIFYDPT-----------------------------------------------------
>BATDEDRAFT_89445       NMGIIELTSFGCTSRKTNTQKIPEGILEVERLLKTVLKNT-NSILIDVREGGGGSMDFAN-GILQLF---KSD----------IEPFKVKCLINEVT-----FNMF---
VESPIDL--KSSRNAWLKTP----AG-SKYSLFHDFTDPKEFNTYDPVYDKP----MGVFTSGNCASACEIMAGTVQSYNIGTVFGEDATTAGSGAVVWSLDPELIFYDSTDFKPMPFKKELTSH-
TNE-----------------------------------
>BATDEDRAFT_90146       NLGIIRLDDFIPKNLKTGARANAEAVREIHKLLSTVLKDT-NAVVFDLRANGGGSGDFAN-AIPQLF---KPD----------FQPMTMRYLMNSVA-----YNTL---
VNGTYFF--DTSNPAWYKTP----PG-SKYSIDHDLNSFNQANMYGQAYLKP----
VGAFTDAVCYSACDLFSASIQSSETGYVLGEDGTTGGGGADVFPLNPTLLLFNHVDFKPMPYYQELTDE---------------------------------------
>BATDEDRAFT_92476       NMGIIKLTSFRCTSRTTNTRDVLKAIREVERLLTTVLKDT-NSVLIDVREGDGGSMRFAN-DIPQLF---KSK----------LEPFKVKYLMSKVT-----YNMF---
VHPPIDS--RSSRNAWSKTP----PK-SKYSVLHDLTDLKTSITYDQVYTKP----MGVFTSGDCASACEIMTGTVQSFKLGTVFGEDVTTAGAGAAVWSLDPELIFYDPTDFKPMPFTRELTSH-
TKDTFYNKMTVGHRALVRSGDHKGKPVEDVGIASDI--
>BATDEDRAFT_92744       NLGIIRLEDFMPKNLKTDAPADVEAVREIHKLLSTVLKNT-NAVIFDIRANGGGSGEFAN-AIPQLF---KPD----------FQPTQVRYLMNSVT-----YNTL---
VNGTYFF--DTSGPAWYNTP----PG-SKYSIDHDRTSYNQSNMYGQAYLKP----
VGAFTNAMCYSACEVFSAALQSSETGYIFGEDGTTGGGGADMFLLDSRLLVFNPVD----------------------------------------------------
>BATDEDRAFT_92841       NMGIIKLTSFECTSRTTNLMSVLEAIREVRGLLKTVLKDT-NSILIDVREGGGGSMRFAN-AIPQLF---KSN----------IEPFKVKYLRNKVT-----YNMF---
VKSPIDL--RSSRNAWSKTS----PDDSKYSLFHDFTDPENIITCSKVYTKP----MGVFTSGNCASACEIVAGTVQSYDIGTVFGEDITTAGAGAAVWNLDLQLTFYDPTDFKPMPFKKELTSH-
```

```
NKEAFYNQ------------------------------
>BATDEDRAFT_93324       NMGIIKLTSFMPTSRTTNTRDVLKAIREVERLLKTVLKDT-NSVLIDVREGGGGCANFAN-GIPQLF---KSK----------LEPFKVKYLRNDVT-----YNMF---
VHPPIDS--KASHDAWLKIS----PK-NKYSVLHDLTDPENTMTYNQVYTKP----MGVFTSGDCASACEIMTGTVQSFKIGTVFGEDVTTAGSGANIWILDPKLINHDPTDFKPMPFTRELTSH-
TKDTFYNKMTVGHRALVRSGDHKGKPVEDVGIASDI--
>SPPG_00409T0   ------------------IMEVYDGIQQLKA------QGA-KRLILDWTDNGGGWGDLGYFAVATLFPNVKRD----------VATFRMTPLLKKLM-----TAAFEKDLNETTPL--
TMFGPSNFVDP----------ATGLSLTSADLNRLILKTHKET----FGKYTSEYSDFVSIVDGGGIADPKRPHNAWNATGIPPPVTPVFPVENVVVLTNGLCGSTCAHSARLIHDEVGVKVFV------------
>SPPG_05166T0   NLGFIRLASFTEVDVEPKV-----AVNAIRDLLVNELKDT-AGLVIDVRGNGGGYIVTAD-TIPQFF---VAD----------YEPTTARALISDNN-----EQIF---VEKGAEG--
QDWKATYLTAN----PG-DKYTGTVSFTSKETANKIGQLYLKP----
VAVFNNAKCYSACDMFSAGMQDHEAALIIGEDATTGAGGANVIEHRSY------------------------------------------------------------
>SPPG_06219T0   GLHISTFDG--------SVDDILKWGDDWLNLLNLAKNNSLKNLILDFCGNGGGKILSGS-ALTFFL---
NPSEYAPPSGGLPLTPTTLKLIGEATNSSSYFYKNFFSDLNGKNMTLADMWMPTWDRPPGTFVPR-
SRKMYMREVLLPRNNEEIRSVAPKEKYFNLAIVTDGYCISTCAIVTNQLRAQNTPI----------------------------------------------------------------------------------
>Hp_gm_asm106-0.164     NLGVIRLVDFLPKTPDGNVQVDL-TISLIRDILTSDLKDT-NAVVFDVRDNPGGAIDIAN-LLPQLF---KPD----------FVPGTARYLRNSIN-----YNLF---
VQKSPPT--DSWHQAWTSSK----DG-DVFSGEGVFDTVPQANQYGQAYLRP----MGVFTNGRCYSACDFFSAAIQDSNTGLILGEDNFTGAGGANVLQH-TDLQAADPADFGNIPLVKELTDS-
YGQSWGSVLTVAIRQAIRSGTHKGQLIEDHGITSDVVV
>Hp_gm_asm236-0.161     NLGILRLENFEPLNENVNA-----AILLIRSILVGPLAET-KAMIVDVRNNGGGLITLAD-YLPQLF---GTH----------FTNMGARDLINSVN-----TNIF---
NSTYYMP--DDFSMAYFSTP----PG-DTYTAPVVFDTDQDSFMIGQAYFKP----VGIFTNGNCYSACDIFTANMQDNELATVFGEDTATGAGGANVVDLNTFLLPLDPTDFSLLPYTSSVSLS-----
IAQNLRVAWRQLVRTGINAGD-------------
>Hp_gm_asm80-0.52       NLGIIRLASFEPINSVSGQYELDAAMTAIRSLLVGPLKDT-NSVVIDLRDNGGGDLTLAN-DIPQLF---KPD----------YNPYGARYLRNSVS-----YDLL---
VKNSPPT--DSWHVAWDQTK----TG-DEWSAIALFDPVSDANKIGQVYVKP----AGILTDGGCYSACDTLSANMQTSGTAIVFGEDGTTGAGGANVWTHSTDLVP-
DATDFHELPFTAQLKPNYNNDMRT--------------------------------

###############
# Asp
###############
>AMAG_11032T0   QYFAEIQIGTPGQSFSVVM--DTGSSNLWVPSTRCMSMACWLHRRFDA--------------------------KKSSTFRKNGTHF----GIQYGSGS--LEGILSTDTVTIG-
DLEILDQT---FGESTKEPGIAFAVGKF-DGILGLGYDN-IA---VE--KVVP--------PFY-SM-IN--QGLLRHPVFTFWLGDVNK-------DPNAGGEIVFGDIDPAHH----------------
TGKIHYAPVVR--------KGYWEVKIEALTV--GGEDFPL-----------------LGNATAA-----
IDTGTSLIAGPMKAADAINKLIGAKKNFLGQ--------------------------------------YMIDC--RKLDS--MPEIAFRFG----------GKDFTLQPEDY----------
VLKVAASPIGGGGGQEQ-----CISGFMGIEMPPQLGQL-----------WIVGDVFLRKYFTVYDLG--NNRVGFAQ-
>AMAG_11644T0   QYFAEIQIGTPGQSFSVVM--DTGSSNLWVPSTRCMSMACWLHRRFDA--------------------------KKSSTFRKNGTHF----AIQYGSGS--LEGILSTDTVTVG-
DLEILDQT---FGESTKEPGIAFAVGKF-DGILGLGYDT-IAVEKVV--PPFY------------QM-IN--QGLLRHQLFTFWLGDVNK-------DPNVGGEIVFGDIDPAHH----------------
VGKIHYAPVIR--------KGYWEVKMEALTV--GGEDFPG-----------------LGNATAA-----
IDTGTSLIAGPMKAADAINKLIGAKKNFLGQ--------------------------------------YMIDC--KKLDS--MPEIAFQFG----------GKDFALQPEDY----------
VLKVAASPIGGGGGQEQ-----CISGFMGIEMPPQLGQL-----------WIVGDVFLRKYFTVYDLG--KNRVGFAE-
>BATDEDRAFT_16209       QYFGEIQIGTPPQPFTVIF--DTGSSNLWVPSTRCSSIACWMHRRYDA--------------------------SESSTYVNNGTEF----AIQYGTGA--
LEGVISQDTVTIG-GLTIENQG---FGESVKEPGITFAVGRF-DGILGLGFDT-IS---VQ--KVVP--------PMY-NL-IN--NHQLDTPLFGVWLGSSSG---------
EEGGEIVFGAVNHDHF----------------KGAVTWVPVVR--------KAYWEVELEGVTI--GGKKLAI-------------------KSSRA----AIDTGSSLFALP-VAEADAINGILGG---
KKNWNGQ---------------------------------FIVDC--ATIDS--LPELTLQFGG----------QKFVITGSDY----------ILQVSAGPVGGGDQ--------
CISGFMGLDIPAPAGPL-----------WIVGDVFLRKFYTIYDVG--NARVGFAEA
>BATDEDRAFT_20107       QYFGVVQLGTPPQSFKVLF--DTGSANLWVPSTRCTDAPCMNHARYDF--------------------------TRSSTFTPNNSPF----AIQYGLGS--
VTGVISSDTLQFA-GINIPNQQ---FAEAVTEPGNTFLTSSF-DGVLGLGFTA-IS--INN--IPTP---------MD-NM-IA--RGLIPAGIFGFFLTRGGV----------
SGSSLTLGGIDTTHV----------------NGAITWIPVVR--------KAYWEVVMDSVTL--GKTTLAT--------------------GSHA----VFDSGTSLIAIP-TVHANQIHQQLGAIPFTNG--
LQ-----------------------------------LIPC--SGLPP-----ITFALN-G---------VAFTLRNEDY----------VLPF---G---FGY--------
CVSVFVGLDMNNF---------------WILGDSFLKFYYTIFDVD--NGRIGIAN-
>BATDEDRAFT_28537       -----------------------------------------------------------------------------------SGEPV----TIKY-KGE-
EYSGFSSDAAVTIP-GTRITGIKLPVLSVKKQSPDLVGINPKF-DGVFGFGYSS-LS--KHH--PQIT--------AMD-VL-YS--NDVIPNNEVSLQLCPYDM---------
LQQSFINIGNTGITPK-----------CG-TDGSSVAWVDSPT--------NDQFTVNIESILV--NGEKVEL-------PAEFQKKV-EHGHTLYS----RVETCFLHMRFPKVVVTALVNAIVGSGAITIK--
NK------VLKRKHK-LSPEDIDNIFWKHHLMIRSDFKIKW--EKLPS--LSITMYAKN-PVTDDNRDSVVKIRLGPRDY----------IQRV---D---YTH--------
DLFSVTAGSNDK----------------ATLGIPFITRLEVTFDLQ--NTRIGFG--
>BATDEDRAFT_87177       -----------------------------------------------------------------------------------TEDVL----SDSYFDGS-
FWKGVSFTATVSIP-KTSISTTDAPVIGIYQQSTRSLVLNDQWNHGILGIAYPS-LT--HSKITSGAT--------IID-SF-YT--SGSIKKNEVAFELCSYEL---------
SKQSYIDIGNTGTVSK-----------CG-TDGSPVVWVETPI--------LNFLTVNIKSIWI--NHQKVQL-------PSTFQYNP-SGGITLFS----
FIDTCAAKILLPRILVMELVNEMIKSRAFQPR------------------PNLQELKLFYFQDYTMKLKEFTIDW--KKLPT--ISFLMSTGT-AALQKNPSSIVNITLGPKDY----------LQKA---G---
PKE--------YVFAVGIGSDQN----------------AILGISFMTRLRIVLDRK--NGLVGFG--
>BATDEDRAFT_89345       -----------------------------------------------------------------------------------SGKTV----TINY-KGD-
EYNGISSTATVTIP-GTRITGIDLPVILVEKQSAYLVGIDNSLKQGILGFGYSS-LS--DYY--TSVT--------AMD-VL-YK--NDVIPRNEIGIQLCPYEM---------
LQESFINIGNTDITSK-----------CG-TDGNSIAWVESPS--------DGYFSVNIKDILI--DDKPVNL-------PAGFQKKM-EDGHTLYS----AVETCSEYMHFPGIVVAALIDAIVCSNAITLD--
SF------N--FKHR-LNNQQIKSLFWKRHLIAESYFNIKW--DRLPT--ITITMFTET-PVTDDNRNSVVEIKLGPRDY----------LQRY---N---SEN--------
LVFAVEVGPNYK----------------ATLGISFMTRLGLVFDRK--HARIGFG--
>BATDEDRAFT_26411       -----------------------------------------------------------------------------------SGEPV----TIGY-KGD-
EYNGFSSTADVTIP-GTGITDINLPVLSVERQSADIVGINPNL-DGIFGFGYTS-LS--NHH--SQTT--------AMD-VL-YT--SDVIPNNEIGLQLCPYEM---------
TSNSFINIGNTDITPK-----------CG-TDGT-----------------------------I--NDEQVEL-------PEEFQKTIMENGRILYS----SVETCFLHMRFPEVVVTALINAILDSGAITVN--
IN------I--FSDK-LGKVMVKKIFSQNYAMLESGFDIDW--GKLPT--LSITMFAEN-PVTDANRNSVVTIKLGPKDY----------IQRA---G---
SKNCKYLTIVCIRFAVGVDSNDN----------------AILGIPFMAQLGLTFDLQ--NKRIGFG--
>BATDEDRAFT_87892       ----------------------------------------------------------------------------------SSEKPV----NINY-NDD-
EYNGVTSTATVTIP-GTGIAGVNLPVLAIQKQSASLVGIGGKLGQGIFGFGHSL-FS--KHY--SQFT--------AMD-IL-YN--DGVIPNNEIGLQLCPYGA---------
ALKSSINIGNTDVTTK-----------CG-TDGKSVAWVQSPK--------NNQFSVNIKSILV--NDKSVEL-------PEEFQQVV-EDGRTLYS----SVETCYMYMCFPEMVVYVLINSILDSDAITVK--
TM---------FKNDHFSKRKIKKKLQKNHLMSKSKYSVDW--DKMPT--LSIVMFSQA-PVTDDNRDSVVTITLGPKDY----------MQII---D---SED--------
FMFTVTACPNDY----------------ANLGTSFMTRLRLTFDLQ--NQRIGFG--
>BATDEDRAFT_26134       ----------------------------------------------------------------------------------LNEEGI----MIEY-KGE-
EYNAFSSTADVTIP-GTKIIDMDLPVIAIQKQSPDLIGIGGRIDQGLFGFAYSS-LS--KRH--PQST--------AMD-VL-YN--SGIIPKNEIGLQICPYGM---------
LSESFINIGNTGVTPK-----------CG-TDGTSVAWVQSPS--------ADHHTVNIKSILI--NGKPVDL-------PERFQQVV-ENGRTLYS----YLQTCFLYMRFPKVVVDVLINTILDSDAITVK--
TM---------FRNDHLGKRKIQRKLRKNHLMSESKYNIDW--GMMPT--ISIVMYSET-PVTDDNRNSVVTITLSPRDY----------MWRY---D---SKH--------
VRFAVAVGSNDY----------------ATLGTSFMVRLGLTFDRT--HKRIGFG--
>BATDEDRAFT_23765       ------------------------------------------------------------------------------------GRLV----TIEY-KGK-
EYRGVSFTATVTIP-GTSITDIGLPVVAVEKQSPDIVGISPNF-DGVFGIGYPS-LS--KRR--PQTT--------VLD-VL-YN--DGVISNNEIGLQLCPYGM---------
RSKSFINIGNTDVTAK-----------CG-TNGRSVAWVDSPS--------HGRHSVNIKSILV--NDELVEL-------PEEFQKNQGEDGRILYS----YLHTCLLYMRFPEVVVTALISAIVDSDAITVK--
NN------MLERKRK-LNPEDIDDIFWKHHLMVRSNFHIDW--IKLPS--LTITMFAQT-PVTDANRNSLVTITLGPKDY----------IQRV---D---SKH--------
VRFTVTAGSNEN----------------AALGISFMARLILTFDLQ--NARIGFG--
>BATDEDRAFT_25259       -----------------------------------------------------------------------------------SGKTV----TINY-KGE-
EYSGFSSTAAVTIP-GTEITGINLPAILVEKKSPDLIGIGGKLKQGVFGFAYPL-LS--NHH--SQTT--------AMD-AL-YS--NNVIPSNEVSIQLCPYEM---------
LQESFINIGNTDITPK-----------CR-TDGKSVAWVQSPT--------TDEFTVDIKSILV--NDKQVDL-------PAGFQKKV-EYGRTLYS----VIHTCFTYICLPEIVVTTLIDAILDSDAITVK--
NT------M--FSNNHLGKRRIKKRLRKNYLMFEPNYNIDW--NKMPS--LSITMFAQT-PVTDDNRDSVVTIKLGPKDY----------MQRI---D---SKY--------
VRFAVKICSSDY----------------ATLGIQFMIRLGLTFDLQ--NTRIGFG--
>BATDEDRAFT_27966       -----------------------------------------------------------------------------------SGEPV----TIKY-RGK-
EYNGVTSTAAVTIP-GTSITDINLPILAVEKLSPYLVGIGGTLKQGVFGFGHSL-LS--DRH--SQTT--------AMD-AL-YT--NDVIPNNEVGIQLCPYDM---------
LQESFINIGNTDITAK-----------CG-TDGNSVAWVQSPS--------NDQFSVNIKSMLV--NGKKVDL-------PEEFQQVV-ENGRALYS----YLRTCSTYMYLPKVVVKALIDAILDSNAITFK--
SI------L--FKNK-LGKKIIKRILQRNSPIPKFDFNIDW--GKLPT--ITIVMFSQN-PVTDANCNSVVTIKLGPKDY----------IQRV---D---SKN--------
YLFTVAAGPNDN----------------VVLGTSFMTRLILTFDRV--HKRIGFG--
>BATDEDRAFT_87185       -----------------------------------------------------------------------------------SGQPV----TINY-KGK-
EYRGISSTADVTIP-GTSITDINLPVLSVEKQSADLVGISPNL-DGVFGFAYSS-LS--KHH--PPIT--------AMD-VL-YS--NGVIPSNEIGLQLCPYDM---------
TSDSFINIGNTDVTAK-----------CG-TDGKSIAWVKSPS--------ADYFTVNIKSILV--NGKRVNL-------PEEFQKRV-EHGRTLYS----IVETCFRYMYFPKVVVAALVDAIVGSGAITIK--
RI------K--LKRK-LYPEEIDDVFWKDGLMAESSFSIDW--GKLPT--VSITIFAEN-PVTVENRNSVVAIKLGPKDY----------IQRY---D---SKN--------
FEFAVKAGSNDK----------------AVLGISFMTRLTMTFDRA--HKRIGFG--
>BATDEDRAFT_92020       -----------------------------------------------------------------------------------SGEPV----TIKY-RGK-
KYNGVSSTAAVTIP-GTRITDINLPVIAVEKQSADIVGISPGF-DGVFGFGYPS-LS--KHH--SRVP--------AID-AL-YN--DGAIPNNEIGLQLCPYGM---------
```

TSESFINIGNTDVTAK-----------CG-TDGTSVAWVQSPS--------NDEFTVNIKSILV--NGKKVDL-------PDEFQKKV-KHGHTLYS----VIHTCCTFMHFPRVVVKALVDAIVDSGAITVR--
KT------KSEHKRK-LNPEEIDGIFLKHYLVPTSNYNIKW--EKLPS--FSITMFSEN-PVTDDNRNSVVEIKLGPRDY----------MQRY---D---SEK--------
FVFAIRVGSNDR----------------AVLAISFMTRLILTFDRV--HKRIGFG--
>BATDEDRAFT_12559   QYYGRIALGTPPQSFLVQF--DTGSANLWLPSTRCSDPACVKHSQFNR--------------------------LLSSTWTSLTQTF----SIQYGTGS--
LSGVMSSDTFYMA-GLTVTNQS---FAESVSQPGTTFINTKY-DGVLGLGMRE-IS---IN--NVAT--------PME-NM-HA--QGLIPAGVFGLYLTKNSA----------
PGSVLTIGGYDPSHV----------------DGSITWLPLSK--------RQFWQVGLTSVTF--NGTTLIQ--------------------NAQA----VFDSGTSLIAIP-
TVSATLIHQQLGAIPYQNG-------------------------------------LQLIPC--TGLPS-----VTFMLN----------NVSFTLRNEDY----------VIPF---G---FGY--------
CVSAFVGLDMHGF---------------WILGDSFMKLYYTIFDSD--NNRIGIAN-
>BATDEDRAFT_87859   ---SYVDRS-
YWFGYAFNGRISIP-GTTIWASQAPITGITIQSTRPLFMSPNI-NGILGISYPE-NA--RYH-GIHGS--------VID-AF-FN--ENTIDKNEVAIQMCSYSL---------
THDSYIDIGNKDIEPK-----------CG-TDGTPLIWIKSPS--------RKFITVTVLRITV--DNQLVKL-------PKGFQRAK-LFTLNQWS----LFDVCSTHTQVPDTVFEALKKAIKTSKAILSH--
IS------------------------------------DLGW--
EGF--FCGKLNMP-------NTIKFD--
>BATDEDRAFT_28725   --SGEPV----AINY-KGK-
EYKGVTSTADVTIP-GTRITGINLPVIAVEKQSPDLVGIDPNF-DGIFGFAYSS-LS--KQH--SQIT--------AMD-SL-YN--DDVIPNNEVSLQLCPYDI---------
LHESFINIGNTDITPK-----------CG-TNGKSIAWVQSPS--------NSYFSVNIKNILV--NDEPVNL-------PAEFQKKKRKNGRTLYS----VVETCFLYMRFPKVVVETLVDAILDSNAITIN--
NN------M--LERK-LNDKEIEDIFWKHYLMSEKYFRTGW--DKLPT--LSITMFAEN-PVTDDNRDSVVTIRLGPRDY----------MQRV---G---FEK--------
FAFAVKAGSNDH----------------AVLGVSFMTRLKLTFDRK--HKRTGFG--
>BATDEDRAFT_25666   --SGEPV----TIYY-KGK-
QYRGISSDAAVTIP-GTRITGIDLPVIAVEKQSPDLVGINPKF-DGVFGIGYTS-LS--DHH--PQIT--------AMD-AL-HN--DGVIPRNEVGLQLCPYDM---------
TSESFINIGNTDVTPK-----------CG-TDGRSVAWVQSPT--------NDEFTVNIKNILV--NGKQVDL-------PVEFQKKV-EHGRTLYS----VMETCFFYMQFPKVVVTALVAAIVDSNGITVK--
NSKSKHKRKSKHKRK-LTPEEIEKIFWEHRLMSKFDFDINW--SKLPP--FSIVMFAEN-PVTDDNSNSVVTIKLGPRDY----------LQRV---N---SKE--------
FVFALKAGPNGN----------------AALGTSFMTRLLLTFDRA--HKRTGFG--
>BATDEDRAFT_28684   --SGEPV----TIEY-KGE-
EYKGISSDTDVTIP-GTSITGINLPVISVEKQSPDLVGINPEF-DGVFGFAYSS-LS--KHH--PPIT--------VLD-SL-YK--DGVIPNNEIGLQLCPSDI---------
LHESFINIGNTDITAK-----------CG-TDGTSLIWVESPT--------TDRHTVNIKNILI--DDEPVEL-------PERFQQVV-ENGRTLYS----YIHTCFIHMRFPEAVVSTLVNAILDSGAITVK--
NN------MLERRHK-LTPEDIDNIFWKLHLMIRSNFRIDW--DKLPS--LTIVMYSET-PVTDDNRNSVVAIKLGPKDY----------IQII---D---SKH--------
VMFSVEVGPSDN----------------AILGTSFMNRLGLTFDRA--HTRIGFG--
>BATDEDRAFT_26132   --SGEPI----TINY-RGN-
DYNAFSSTAAVTIS-GTEITGIDLPVLAVQKKSPDLFGINPNF-DGVFGFGHPS-LS--EHH--SPIT--------AMD-VL-YN--GGVISNNEVSLQLCSHDI---------
FQQSFVNIGNTDITPK-----------CG-TDGTSVAWVQSPT--------NDCHNVNIKSILI--NGEQANL-------PAEFQKKV-EDDHTLYS----YIQTCSSYMIFPETVVTALVGAILDSGAITIK--
NN------L--FTSK-LSKREIKKIFWRSYAMPESTYNIDW--NKLPS--LSITMHAET-PVTLENYNSVVTIKLSPKDY----------IQRV---N---SER--------
FLFAVAVGSNDR----------------AALGIPFMTRLTVTFDRA--HKRIGFG--
>BATDEDRAFT_27290   --SGEPV----TIDY-KGD-
EYKGVTSTAVVTIP-GTRITDINLPVLAVQKQSPDSVGINPEF-DGVFGFAYPS-LS--KHH--PPIT--------AMD-AL-YT--NDVIPNNEIGLQLCPYEM---------
TSNSFINIGNTDVTAK-----------CG-TDGTSIAWVQSPS--------DDQFTVNIKSILV--NDEPVDL-------PEEFQKTIMENGRILYS----SVETCFLHMRFPEVVVDVLINAILDSGAITVN--
IN------I--FSDK-LGKVMVKKIFSQNYAMLES---
VVFAIKAGPNDN----------------AILGIPFMAQLGLTFDRA--HKRIGFG--
>BATDEDRAFT_27296   ---SSGKPV----TIKY-KGN-
EYRGISSTAVVTIP-STRITGINLPVLSVEKQSAGLVGIGGNPDQGVFGFGYSS-LS--NHH--SRVT--------AID-AL-YN--GGVIPKNEVSIQLCPYKM---------
LSKSSINIGSTDVTPK-----------CG-TDGKSVAWVQSPS--------DDQFTVNIKNILV--NGKQVKL-------PEEFQKNQGKNGRILYS----YIHTCFVYMYFPRVVVAALVDAIVGSNAITVK--
KT------KSEHKHK-LSETEIEKVFWENRLILKSKLNINW--GRLPS--LTITMFAEN-PVTDDNRNSVVTIKLGPRDY----------LQRV---N---SKE--------
SRFTVTAGSNDK----------------AVLGIPFMNRLLLTFDRT--HKRIGFG--
>BATDEDRAFT_87250   --SGEPV----TIKY-KGD-
EYNGITSTADVTIP-GTRITDINLPVIAVEKQSADLVGIDPNL-DGIFGFGYPL-LS--KHH--SRIT--------AMD-IL-YS--NNVIPNNEISIQLCPYDM---------
TSDSFINIGNTEITAK-----------CG-TDGKSVAWIQSPT--------TDHHTVNIKNILV--NDKQVDL-------PEEFQQVV-ENGRTLYS----YLQTCSTYMIFPKVVVATLVDAIVGSNAIIFK--
SM---------FSKK-LSKREIENIFWENHPMIESTYNIDW--EKLST--ITITVFSQT-PVTDDNRNSVVTIKLGPRDY----------IQRY---D---SED--------
CKFAIKAGSNDK----------------AILGIPFMTRLELSFDRA--HKRIGFG--
>BATDEDRAFT_22611   --SGEPV----TIDY-KGD-
EYKGVTSDTAVTIP-GTSITDINLPVVAVEKQSPDLVGIDPEF-DGIFGFGNSL-LS--KHH--PQTT--------AMD-VL-YT--SDVIPNNEIGLQLCPYEM---------
LSDSFINIGNTDVTAK-----------CG-TDGTSIAWVQSPL--------NDEFTVNIKSILI--NDEPVNL-------PEEFQKTIMENGRILYS----SVETCFLHMRFPEVVVTALVDAILDSGAITVN--
IN------I--FSDK-LGKVMVKKIFSQNYAMLESGFDIDW--GKLPT--LSITMFAEN-PVTDANLNSVVTIKLGPKDY----------IQRV---D---SEN--------
CKYLTIAGPNDN----------------AILGIPFMAQLGLTFDRT--HKRIGFG--
>BATDEDRAFT_28256   --TLNEEGI----MIEY-KGE-
EYKGVTSTAAVTIP-GTKIIGMDLPVIAIQKQSPDLIGIGGRIDQGIFGFAYSS-LS--DQH--PQST--------AMD-VL-YN--SGIIPKNEIGLQICPYGM---------
LSESFINIGNTGVTPK-----------CG-TDGTSVAWVQSPS--------ADHHTVNIKSILI--NGKPVEL-------PAGFQKKV-ENGRTLYS----YLQTCFLYMRFPKVVVDVLINTILDSDAITVK--
TM---------FRNDHLGKRKIQRKLRKNHLMSESKYNINW--DMMPT--ISIVMYSET-PVTDDNRNSVVTITLSPRDY----------MWRY---D---SKH--------
VRFAVAVGSNDY----------------ATLGTSFMVRLGLTFDLQ--NTRIGFG--
>BATDEDRAFT_24293   --SGEPV----SIKY-KGK-
QYNGVTSTAVVTIP-GTGITGVNLPVLAVEKQSADSVGINPKF-DGIFGLAYSS-LS--NQH--SRVT--------AMD-AL-YN--DGAIPKNEVSLQLCPYGM---------
TSESFINIGNTDVTAK-----------CE-TDGTSVAWVESPS--------NNQFSVNIKSVLV--NGRPAEL-------PAEFQKKV-KDGRTLYS----SVETCFLNMRFPETVVTALVDAILNSNAITVK--
DN------M--LRSDYLDKKKIKMRLWKNHLMPRYKYTIDW--SRMPR--FSITMFAQT-PVTDENRDSVVTIELGPRDY----------MQIISIEN---SEK--------
FLFAVTVGSNDY----------------AVLGMSFMTRLKLTFDLQ--NARIGFG--
>BATDEDRAFT_21660   ---------ISVNGVNLKMMVDSGSFDTIVPFSTLNNYN-----------------------------------GPSLTYTMHETSI----FGSYDGVT-
AWEGHGFLATVRIQ-KTSIIVKNAPVIGIFKQSTDSPLIDGNIEQGILGLAYPS-LA--EYK-ATPPT--------VMD-AL-YD--SKAISNNEVGLHLCPYEL---------
SKKSYIDIGNTNLAPK-----------CG-TNGNPITWIYSPT--------QDYFTVDIKSILI--NGNPVTL-------PNEFQVKTPGDKMHPFS----
IIDTCTTTMDLPHEVVAILIDEIVGSDAFEPK------------------LDDHTALIFFWERYIIKKSHYTINW--KKLPV--LSFVMSTGV--SNSESDSSIVKITLGPKDY----------IQKI---S---
STK--------YLFSVSAGSNKN----------------AVLGTSFMARLKFVLDRE--HVRIGLG--
>BATDEDRAFT_4465   --TTRISLGTPGQSFDVSL--DTGSDILWARSSICTGNGCASGRKYN---------------------------PALSSTFINGHTA--WSIINYADGT-
AVNYTLGNDALTIG-TVSQPNVTFAIPTSVTMPPSVPGT-----DGIIGLAPPS-FN--NQG--TDTQ--------FMQ-WL-----SYSFAVRQVSVWHDVMVK--
EDQNDRSDNGGTITYGGIEPGRS----------------SGTPHWLPIQL--TNIPGSSNYWVVNMDSITF--GGGAIPM-------PLDASMA--------------
LVDTGTAQLVLGQQLFDVVNQQMGGIDNGAGQ--------------------------------------YTLDC--TKAKA--FPNLVLTLG--------GKAFTLTSSQQYY----------------TD---
GKI--------CVSILGRAQDGEQR--------------TILGVAFLRQYYVTFDYD--RWAVGI---
>BATDEDRAFT_23205   --SGEPV----TIDY-KGE-
EYKGISSDTDVTIP-GTSITDINLPVLSVEKQSADLVGINPEF-DGVFGFAYSS-LS--NHH--PPAT--------AMD-AL-YN--HDVIPNNEIGLQLCPYEM---------
TSNSFINIGNTDITAK-----------CG-TDGTSIAWVESPS--------DDQFTVNIKSILV--NDEQVDL-------PEEFQKTIMENGRTLYS----SVETCFLYMRFPEVVVAALVDAILDSGAITVN--
IN------I--FSDK-LGKVMVKKIFSQNYAMLESGFDIDW--GKLPT--LSITMFAQT-PVTDANCNSVVTIKLGPKDY----------IQRA---G---SKN--------
FAFAIKAGPNDN----------------AILGIPFMTRLGLTFDRA--HTRIGFG--
>BATDEDRAFT_22593   --SGEPV----NIKY-KDK-
EYKGVTSTAAVTVP-GTAITGVNLPVIAVEKQSARLAGIDPDF-DGVFGIGHFS-LS--KHH--SPIT--------AMD-SL-YS--NNVIPNNEIGIQLCPSDL---------
FEQSFINIGNTEITAK-----------CG-TDGRSVAWVNSPT--------NDHHTVNIKSILV--NDEQVDL-------PEEFQKTV-EDDHILYS----HVETCSKYMHFPQTVVIALVKAIVDSDAITIW--
DA---------KSRRKLNPEEIDDIFWKHRLMSESNFNIDW--RKLPT--FTIVMYAQT-PVTDDNRDSVVKIRLGPKDY----------IQKV---NLGKFKR--------
YLFAVTVGSNDN----------------AILGIPFMTQLELTFDLL--NTRIGFG--
>BATDEDRAFT_25680   --SGEPV----TIKY-KGE-
EYNGVTSTATVTIP-GTSITDINLPVLAVEKQSADSVGINPNL-DGIFGFAYPL-LS--NQH--PQVT--------AMD-AL-YN--GGAIPSNEVSLQLCQYDM---------
LHESFINIGNTEITPK-----------CG-TDGKSVAWVQSPT--------NNQFSINIK--ILPEKLVATLINAILDSNAITSK--
NI---------LLRRKLDKEKVKKKLQANSPMFKYNYNIKW--EKLPT--VSIVMFAQT-PVTYDNSNSVVTIKLGPRDY----------MWKY---D---SEK--------
FRFSIKAGSNDK----------------AALDIPFMSQLAVTFDRT--HKRIGFG--
>BATDEDRAFT_23274   ---SSGKPV----TIKY-KGN-
EYRGISSTAVVTIP-STRITGINLPVLSVEKQSAGLVGIGGNPDQGVFGFGYTS-LS--NHH--SRVT--------AID-AL-YN--GGVIPKNEVSIQLCPYEM---------
LSKSSINIGSTDVTPK-----------CG-TNGKSVAWIQSPS--------DDQFTVNIKSILV--NGKQVEL-------PEEFQKNQGKNGRILYS----YIHTCFVYMRFPETVVAALVDAIVGSNAITVK--
KT------KSEHKRK-LSETEIGKIFWENRLIPKSKLNINW--GRLPS--LTITMFAEN-PVTDDNRDSVVTIKLGPKDY----------LHKY---D---SKH--------
ARFTVKSGPNDK----------------AVLGIPFMTRLRLIFDQT--HKRIGFG--
>BATDEDRAFT_89380   ---YPEHADAV----TLKYDGGA-
SAKGHGFLTTVSIP-ETTISIKSAPVVGISEQSENPVIIDGNPNQGVFSFAYPS-IA--MYH-AKPPT--------IMD-AI-YE--SGAVPNNEVAMELCPYGL---------
SEKSYIDIGNTETDPK-----------CD-TDGQPVIWVQSPS--------RDRFTVNIKEIRI--DNKARRL-------PSEFQVKESEDIWHPYS----
FISTCSKYISLPRKIVDKLIRAIGKSGAFKPK------------------LTKDDIKKFFEKKQTIKVPDHIIDW--KKLPK--LSFVMYTET-SKTGKASRSSVEITLGGRDY----------IQQV---G---

```
AEE--------YQFFVDVGRNKR----------------VVLGTPFMLRMRVVFDRT--NQRIGFG--
>BATDEDRAFT_21133       ---------------------------------------------------------------------------------SNGQPI----TIKY-KYS-
DYYGVIPTVAVTIP-GTRITDTSLPILAVKKLSADSVGIGGTLKQGIFGFGYTS-LS--NHH--SKTT--------AMD-IL-YN--NDVIPNNEVSLQLCPYDM---------
TSESFINIGNTDVAAK-----------CG-TNGKSIAWVESPS--------DDRHTVNIKSVLV--NDKQVDL-------PEEFQKRM-ENGRILYS----TVETCFLYMSFPETIVKALVDAIADSDAITVK--
YN--------VYRIDFLNRQDFNDIFWRHHLMPEAIMFIKW--NKLPT--LTITMFAET-PVTDENRDSIVTIKLGPRDY----------IQKV---D---FKN--------
LVFAVKADSNDH----------------AVLGASFMSRLGLTFDRQ--NARIGFG--
>BATDEDRAFT_25355       ----------------------------------------------------------------------------------SGEPV----AINY-KGE-
EYRGFSSTAVVTIP-GTRITGINLPVILVEKQSPDLVGINPKF-DGVFGFGYLS-LS--SHY--TSVT--------AMD-AL-YS--YGVIPNNEISIQLCSYDM---------
LQDSFINIGNTDVTAK-----------CG-TDGKSVAWVNSPI--------ADQFTVDIKSILI--NGKNVNL-------PAEFQQVV-ENG-----------------------LVTMLIDAILDSGAITVK--
NT------M--LSNDYLDKRRIKKRLKENRLMSESTYNINW--DKMPT--ISITMHAET-PVTYDNSNSVVTIELGPRDY----------MQRI---D---SKY--------
VVFAVKVGPNYK----------------ATLGISFMTRLGLVFDRK--HARIGFG--
>BATDEDRAFT_27069       ----------------------------------------------------------------------------------SGDPV----TIKY-KGD-
EYKGISSKAVVTIP-GTRITDSKLPVIAVKKQSPDLIGISPNL-DGVFGIGYSS-LS--KHH--PPAT--------AMD-IL-YN--GNTIPNNEVGLQLCPYDM---------
ISNSFINIGNTDITAK-----------CG-TNGRSVAWVDSPS--------DGYFSVNIKSILI--NGEQVDL-------PAEFQKRILKNGYALYS----RVETCFTFMQFPERVVATLIDVIVGSNAIIFK--
KK------K--LSDK-LNKEMVKRKLRKNRPIFKSNYNIDW--SKLPT--VSIVMFSQN-PVTDENRNSVVTIKLGPKDY----------IQII---D---SKH--------
VRFTVKVGSSDH----------------AILGISFMTRLLLTFDRA--HKRIGFAP-
>BATDEDRAFT_27767       ----------------------------------------------------------------------------------SGEPV----AINY-KGK-
EYKGVTSTADVTIP-GTRITGINLPVIAVEKQSPDLVGIDPNF-DGIFGFAYSS-LS--KQH--SQIT--------AMD-SL-YN--DDVIPNNEVSLQLCPYDI---------
LHESFINIGNTDITPK-----------CG-TDGKSVAWVQSPS--------NSYFSVNIKNILV--NDEPVNL-------PAEFQKKKRKNGRTLYS----VVETCFLYMRFPKVVVETLVDAILDSNAITIN--
NN------M--LERK-LNDKEIEDIFWKHYLMSEKYFRTGW--DKLPT--LSIVMFAEN-PVTDDNRDSVVEIKLGPRDY----------MQRV---G---FEK--------
FAFAVKAGSNDH----------------AVLGVSFMTRLKLTFDRK--HKRTGFG--
>BATDEDRAFT_27286       ----------------------------------------------------------------------------------SGEPV----TIDY-KGE-
EYNGVTSTADVTIP-GTRITDIDLPVIAV--------GIDSML-NGVFGFGNSL-LS--NHH--PQTT--------AMD-AL-YN--GGIIPNNEVSLQLCPYEM---------
TSNSFINIGNTDVTAK-----------CG-TDGTSVAWVKSPS--------DDQFTVNIKSILV--NDEPVEL-------
PEEFQKTIMENGRTLYS-------------------------------------------------------------------------------------------------------------------------
--------------------------------------------------------------------------------------
>BATDEDRAFT_26748       ----------------------------------------------------------------------------------SGEPV----TIDY-KGD-
EYNGFSSTATVTIP-GTRIAGINLPVLAVEKQSPDSVGISSKF-DGIFGLGYPS-LS--NHH--PPAT--------AMD-AL-YT--NDVIPNNEIGLQLCPYEM---------
TSNSFINIGNTDVTPK-----------CG-TDGTSVAWVNSPS--------DNQFSVNIKSILV--NDEPVDL-------PEEFQKKV-ENGRTLYS----SVETCFLHMRFPEAVVEALVDAILDSGAITVN--
IN------I--FSDK-LGKVMVKKIFSQNYAMLESGFDIDW--GKLPT--LSITMFAQN-PVTDANCNSVVTIKLGPKDY----------IQRA---G---SKN--------
CKFAIKAGSNDN----------------AVLGIPFMAQLGLTFDLQ--NKRIGFG--
>BATDEDRAFT_90861       ---GPLQIGTPPQTIQVQF--DTGSSLTWIRSSSCKTGSGCGGPKYD---------------------------PSRSLTWRPGTGF--TQSMTYADGG-
SISCEEQLDVVHLA-GTQLVSES---ICSANMITNAVNQTDML-GGLVGMSPPL------GK--DNTN--------LML-TL-----EQSFGLSMVSFWYNQMAV--
EGDHGLISGAGEITFGGYNPARF----------------SGDIEWLPVIP-------NTGYWSVKLDGMTF--GDMIVPV----------------RKELSSQT----IFDTGTTLIALPSDMF-
TVMNTIMYAELDPVG--GM----------------------------------YKLDC--KHATT--LPP-----------------VTLFFGGKPY----------MMTWSEQILTDNVD--------
CWSVFTPTLNMG----------------IIFGVSFLRNYHVTFDYTSTLMQVGLA--
>BATDEDRAFT_24767       ----------------------------------------------------------------------------------NGEPV----TIDY-RGK-
QYKGVTSDTVVTIP-GTRITDIKLPVLAVRKQSPDLVGIDPEF-DGIFGFGNSL-LS--NHH--SRIT--------AMD-VL-YN--DGVIPNNEVSLQLCPYEM---------
TSNSFINIGNTEIAAK-----------CG-TNGRSVAWVDSPP--------DGRHTVNIKSILI--NDKPVNL-------PEEFQKRV-KDGRTLYS----VIQTCFRYIRFPEVVVKALIDAILDSGAITVK--
KA------M--YSNK-LNSEEIKRIFWEHYAMLESKLNIDW--GKLPT--LSITMFAQN-PVTDSNRNSVVTIKLGPKDY----------IQRY---D---SER--------
FLFIVAAGSNDY----------------AALGIPFMTRLGLTFDRA--HKRIGFG--
>BATDEDRAFT_28181       ----------------------------------------------------------------------------------SGEPV----SIKY-KGK-
QYNGVTSTAVVTIP-GTSITDIKLPVVAVERQSADIVGIGGTLKQGVFGIGYSS-LS--DHH--PPIT--------AMD-VL-YK--DGIIPNNEIGLQLCPYDI---------
LHESFINIGNTDITPK-----------CG-TDGKSVAWVDSPT--------NNQFSVNIK--------------------------------------------------ILPEKVVTTLINAILDSNAITVK--
GS------M--LRRK-LGKEEIKKKLQANSLMFKSNYNIKW--
DRM---------------------------------------------------------------------------------------------------------------------------
>BATDEDRAFT_24760       ----------------------------------------------------------------------------------NGEPV----TIKY-RGK-
EYSGFSSDAVVTIP-GTGITGVNLPVIAVEKQSPDLVGISPNL-DGVFGFAYSS-LS--KHH--PPAT--------AMD-TL-YN--DGVIPNNEVSLQLCPYDM---------
TSDSFINIGNTEIAAK-----------CE-TDGRSIAWVESPT--------TDRHTVNIKSILI--NGEPVEL-------PAEFQKKV-EHGHTLYS----VIQTCFIHMYFPRVVVKALVDAILDSDAITVK--
KT------V--FSNK-LNKKEVKRIFWEYHAMSESGFNIDW--GKLPS--FTIVMHAQT-PVTDDNSNSVVKITLGPRDY----------IQKI---D---YTR--------
LVFAVTTGSNDK----------------AVLGIPFMTHLAVTFDLQ--NKRIGLG--
>BATDEDRAFT_12639       QYYGRIALGTPPQSFLVQF--DTGSANLWLPSTRCSDPACVKHSQFNR--------------------------LLSSTWTSLTQTF----SIQYGTGS--
LSGVMSSDTFYMA-GLTVTNQS---FAESVSQPGTTFINTKY-DGVLGLGMRE-IS---IN--NVAT--------PME-NM-HA--QGLIPAGVFGLYLTKNSA----------
PGSVLTIGGYDPSHV----------------DGSITWLPLSK--------RQFWQVGLTSVTF--NGTTLIQ--------------------NAQA----VFDSGTSLIAIP-
TVSATLIHQQLGAIPYQNG-------------------------------------LQLIPC--TGLPS-----VTFMLN----------NVSFTLRNEDY----------VIPF---G---FGY--------
CVSAFVGLDMHG-F--------------WILGDSFMKLYYTIFDSD--NNRIGIAN-
>BATDEDRAFT_89821       -----------------------------------------------------------------------------------EKPV----SINY-KGN-
DCKGVSSEAAVTIP-DTRITGVNLPVIAVEKQSADLIGINERF-NGIFGFGHSL-LS--NHH--TSTT--------AID-VL-YS--KNVIPNNEVGLQLCPYEM---------
SQESFINIGNTDITPK-----------CG-TDGRSVAWVESPS--------DDHFAINIKDILV--NDKPVNL-------PAEFQKTIMENGHTLYS----VVETCSAYMQFPKVVVAALVSAILDSGAITAK--
NT------M--FRSK-LDKDEIQGIFWGNYLMPELNFNINW--DMMPT--ISVAMFAEN-PVTVDNRNSIVMIELGPKDY----------MQIV---D---SKY--------
VVFAIKVGSNDN----------------ANLGIPFMTRLVLTFDRK--HKRIGLG--
>BATDEDRAFT_27037       ---STIQVGTPPKDFVVHF--DTGSAVFWLRSNACV------GGNNCGQPTMATFTP-----------------SSSSSFSLVNSTY---KPFTYADGT-
AVTCNVGQDILSIG-GLSIPNQQ---LCLANSITS---NTPLH-DGIIGLAPPS------VM--EPTS--------VFS-ML-DT--NKAFAASQVSFWYNSSSL-----
VDGHVQSGVVTVGGVDPSLF----------------KGDIRWFPAML------ANGRHWSIPLTNITLRDGSKIVPT------------------
QKDLFSPPLIIIDSGSTYNFLPRFMFDPLNAIHFQAKDIDGT--GT----------------------------------YAFDC--NAVPN--LSPIILTLA----------GVDIKIMPEEQ----------
YFKF-------RQY--------CIILFTTSDRPNST--------------PLLGVGFMRFRYTVFDHK--KFKIGFST-
>BATDEDRAFT_26151       -----------------------------------------------------------------------------------GRHV----TIDY-KGK-
EYRGISFTATVTIP-GTEITGIGLPVVAVEKQSPDIVGISPNF-DGVFGIGYPS-LS--KRR--PQIT--------VLD-SL-YN--GGTIPNNEIGIQLCPYGM---------
TSNSFINIGNTDVTAK-----------CG-TDGTSVAWVNSPT--------TDRHTVNIRIILV--DDEQVEL-------PERFQQVV-ENGRTLYS----YIHTCFVYMRFPEVVVTALVDAILDSGAITAK--
NS---------MLKRKLSPEDIDNIFWKHYPMPKSSFNIEW--NKLPS--LTIVMYSET-PVTDDNRNSVVAIKLGPKDY----------LKRI---D---SKN--------
YLFSVEVGPSDN----------------AILGIPFMNRLIVTFDLQ--NARIGFG--
>BATDEDRAFT_90978       ----------------------------------------------------------------------------------SGEPV----TIKY-KGE-
EYSGFSSDAVVTIP-DTGITGIKLPVLAVKKQSPDIVGINPKF-DGVFGFGYSS-LS--KHH--PPAS--------AMD-VL-YS--NDVIPNNEVSLQLCPYDM---------
LQQSFINIGNTGITPK-----------CG-TDGSSVAWVDSPT--------NDQFTVNIKSILV--NGEKVEL-------PAEFQKKV-EHGHTLYS----RVETCFLYMRFPKVVVTALVNAIVGSGAITIK--
NK------VLKRKHK-LSPEDIDNIFWKHHLMIRSDFKIKW--EKLPS--LSITMHAKN-PVTDDNRDSVVTIKLGPKDY----------IQII---D---SKH--------
DLFSVTAGSNDK----------------ATLGIPFITRLEVTFDLQ--NTRIGFG--
>BATDEDRAFT_25784       ----------------------------------------------------------------------------------NGQPF----TIKY-KNE-
EYRGISSDAAVTIP-GTGITGINLPVLSAEKQSPDLVGIHPKF-DGVFGFSYSS-LS--KHH--PPAT--------AID-AL-YK--DGVIPNNEIGIQLCPYEM---------
LQESFINIGNTDVAAK-----------CG-TNGKSVAWVQSPS--------DGYFSVNIKSILV--NGKQVDL-------PEEFQQVV-ENGRTLYS----YLHTCFTYIRFPKTVVDVLINAILDNNAITTK--
SI---------LFRNKLGKIMIKSSLKNNYLMFQSDYNIDL--DKLPT--LSIVMFAQT-PVTDDNSNSVVTIKLGPRDY----------MQRY---D---SKN--------
FVFTVEAGPNDN----------------AVLGASFMTRLAITFDLL--NIRIGFG--
>BATDEDRAFT_20117       QYFGVVQLGTPPQSFKVLF--DTGSANLWVPSTRCTDAPCMNHARYDF--------------------------TRSSTFTPNNSPF----AIQYGLGS--
VTGVISSDTLQFA-GINIPNQQ---FAEAVTEPGNTFLTSSF-DGVLGLGFTA-IS--INN--IPTP---------MD-NM-IA--RGLIPAGIFGFFLTRGGV----------
SGSSLTLGGIDTTHV---------------NG-AITWIPVVR--------KAYWEVVMDSVTL--GKTTLAT--------------------GSHA----VFDSGTSLIAIP-TVHANQIHQQLGAIPF-----
TN--------------------------------GLQLIPC--SGLPP-----ITFALN---------G-VAFTLRNEDY----------VLPF---G---FGY--------CVSVFVGLDMNN-
F--------------WILGDSFLKFYYTIFDVD--NGRVGIAN-
>BATDEDRAFT_28541       ----------------------------------------------------------------------------------NGEPV----IIDY-RGK-
EYRGISSDAVVTIL-GTGITDINLPVIAVEKQSARLAGIDPNF-DGVFGFGYPS-LS--KHH--PPTT--------VLD-TL-YS--NNVIPNNEIGIQLCPYGM---------
RSKSFINIGNTEITAK-----------CG-TDGRSVAWVNSPT--------NDHHTVNIKSILV--NDEPVDL-------PEEFQQVV-KDDHTLYS----VVETCSTYMYFPKVVVTALVKAIVDSDAITIK--
KN------IFKDERK-LTPEEIDDIFWKHYLMVRSNFHIDW--IKLPT--FTITMYAQT-PVTDDNHDSVVKIKLGPKDY----------IQRV---D---YTH--------
NMFAVKVGSNDK----------------AILGIPFMTQLELTFDLQ--NIRIGFG--
>BATDEDRAFT_28166       ----------------------------------------------------------------------------------SGEPV----TIDY-KGK-
KYSGISSTADVTIP-GTEITDINLPVIAV--------GIDPMF-NGVFGFGYTL-LS--NHH--SRIT--------AMD-AL-YN--GGIIPNNEVSLQLCPYEM---------
LSDSFINIGNTEITAK-----------CG-TDGSSVAWVNSPS--------DDQFTVNIKSILV--NDKRVNL-------PAGFQKRV-KDGRTLYS----VVETCCTCMHFPKVVVKALINAIVGSGAITIK--
NN------M--FRRK-FDQRDINNLFWKHHLMVEPKLNIDW--SKLPT--LSIVMYAQN-PVTVDNYNSIVEITLGPRDY----------MQKI---D---SER--------
YLFAVTVGSNYN----------------AALCIPFMTRLTMIFDRA--HKRVGF---
>BATDEDRAFT_89831       ----------------------------------------------------------------------------------NGEPV----TIDY-KGD-
```

EYSGVSSDAVVTIP-GTRITDIKLPVLAVRKQSPDLVGIDPEF-DGIFGFGNSL-LS--NHH--SRIT--------AMD-AL-YN--GGIIPNNEVSLQLCPYEM---------
TSNNFINIGNTDVTAK-----------CG-TDGRSVAWVDSPP--------DGRHTVNIKSILI--NDKPVNL-------PEEFQKRV-KDGRTLYS----VIQTCFRYIRFPEVVVKALIDAILDSGAITVK--
KA------M--YSNK-LNSEEIKRIFWEHYAMLESKLNIDW--GKLPT--LSITMFAQN-PVTDANCNSVVTIKLGPKDY----------IQRY---D---SKH--------
VLFAVKAGPNDN----------------VILGIPFMTRLGLTFDRA--HKRIGFG--
>BATDEDRAFT_90267   --GQPV----TINY-KGD-
EYNGISSTTAVTIP-GTRITGIDLPVLAVQKKSASLIGINPKF-DGVFGFGHPS-LS--KHH--SPIT--------AIN-VL-YN--DGVISKNEVGLQLCPYEM---------
LSDSFINIGNTDITEK-----------CG-TNGKSIAWVQSPP--------NSYSTVNIKSILV--NGEKVDL-------PAGFQKKV-EHGRTLYS----SLETCSTYMHFPETVVAALISAILDNGAITVK--
SN------L--FDDQ-LSKEEIEKIFLKHYLMPTSNYNIKW--DRMPT--LSITMHAET-PVTVENRNSVVTIKLGPRSY----------MQRY---D---SKR--------
FEFAIEVGSDYY----------------AALGIPFMNQLGLTFDRR--HARIGFG--
>BATDEDRAFT_28328   ---SGEPV----TIKY-KGK-
EYNGVTSTATVTIP-GTSITDINLPVVAAERQSADSVGIGGKLGQGMFGFAYSS-LS--NHH--SQTT--------AMD-TL-YS--NNVIPRNEISIQLCPYEM---------
LSDSFINIGSTDITPK-----------CG-TDGSSVAWVSSPS--------NDRHTVNIKNILV--NGKKVKL-------PEEFQKRV-KDGRTLYS----SVETCFLHMRFPKAVVKALIDAIINSDGITIK--
YN--------GFRNNYLDKRMIKNRLKENRLMARYKYDIDW--SRMPS--FTIIMFAEN-PVTDDNRDSVVEIRLGPRDY----------MQRV---SIRNSEK--------
FWFTVTAGSNDY----------------AVLGMSFMTRLKLTFDRT--HKRIGFG--
>BATDEDRAFT_90524   ---SGEPV----SIKY-KGD-
EYNGVTSTAAVTIP-GTSITDINLPVVAVERQSPDLVGIGGTLKQGVFGFGYTL-LS--NHH--SRIT--------AMD-AL-YN--DGAIPKNEVSLQLCQYDM---------
LHESFINIGNTDITPK-----------CG-TDGKSVAWVDSPT--------NDEFTVNIKSILV--NGKQVEL-------PAEFQKKQGKDGRTLYS----YLQTCFLCIVLPEKVVTTLINAILDSNAITVK--
GS---------MLRRKLGKEKVKKKLQANSLMFKSNYNINW--DRMPT--ISVVMYAQT-PVTYDNSNSVVTIKLGPKDY----------MWKY---D---SEK--------
FRFTVKSGSNDK----------------ATLGMSFMSQLAVTFDRQ--NKRIGFG--
>BATDEDRAFT_28176   ---SGKPV----TIRY-KSR-
KYKGFSFDTDVTIP-GTRITGINLPVIAVKKQSASLVGIGGNPDQGVFGIGYSS-LS--DHH--SRVT--------AID-AL-YN--GGVITNNEVGLELCPYKM---------
LSKSSINIGNMDVTPK-----------CG-TDGRSVAWVDSPT--------NDQFTVNIKSILV--NGEQVDL-------PKEFQKHILKNGHTLYS----YLQTCFVYMYFPRVVVETLVAAIVGSDAITVK--
KT------KSKHKHK-LSKTEIKKIFWENRLILKSKLSINW--DRMPT--ISITMFAEN-PVTDDNRNSVVTITLGPRDY----------LQRY---D---SKY--------
VRFIVEAGPNEN----------------AALGIPFMNRLRLVFDRQ--NKRIGLG--
>BATDEDRAFT_23192   --GQPV----TIKY-KGK-
EYNGITSTADVTIM-STEITGVNLPVIEIEKESTDLVGIVEDF-DGVLGIGYLP-LS--DYY--PPVT--------AMD-VL-YN--GGFISNNEVGLQLCPYEM---------
LQESFINIGNTEITPK-----------CG-TDGKSLIWVNSPS--------DEHHAVNIKSILV--NGKQVDL-------PEEFQQVV-ENGRILYS----TIHTCYMYIRFPERVVNVLINSILDNSAITIK--
KT------M--LRDDRLDRKKIKKKLQKNRLMPKSNYNIDW--DKMPS--LSIVMFSQN-PVTDENRNSLVTIRLGPRDY----------MQTY---N---FKN--------
FRFTVTAGSNDH----------------ATLGIPFMTRLLLTFDRQ--NKRTGFG--
>BATDEDRAFT_90888   --FMTINVGT---------
PPVTL--NVQIDTGSSTFWIVSTLCNAGNGCTPAVTANAF----------------NPTK-------------------RSVINIPRVSFYYDQSVN-SLEATQPIASSGEITFGTPNPARY----------------
TGNFTWLPTIP-------NMSQWAVGIDYIIA--GGQTVVT-------------------TQMF-----
LFDTGTTLIYLDQQLFPFINTAMGGISKQGGI--------------------------------------YELDC--SLVHT--LQPITFSLG--------GIGARLTLSWN------------MQYFTLDN----
TR--------CISIFTTSPSQS----------------NIFGGIFLQHFYTSFDYN--ESRIGLAA-
>BATDEDRAFT_28984   --------------TTFSVMIDSGSTDLTVPLTGLNNYVG---------------------------------PTINTPRPPGSGD---LIGTYGDQS-
GWTGYGFQGTARVT-GTNITASNAPVIGMFRQTTNPIYTTGDVTQGLLGIAYPS-LA--SYR-VTPST--------VVD-AW-VA--SGTMPKNEIAFHACPYSM---------
TNQSYVDFGNDDPSYK-----------CS-SNGVPVVWAYSPA--------RTYFTIDVRAISI--DSVPVTL-------PQTFQNGI---GYNKWS----
LVDSCTSVMILPGSVITTLVNAIQSSGGLPSD------------------LASSPHLSNFLSGSIGIRPVQPFQW--SKLPS--ISFDITSDQ--IRPGGQNATFKITLGARQY----------IQPN---A---
NGY--------FAMIARSGGERY----------------ANLGIPLFSNLNVVLDRA--NARVGFSQ-
>BATDEDRAFT_28688   ---SGEPV----TIDY-KGD-
EYSGVSSNAVVTIP-GTSITDINLPVVAVQKQSPDLVGINPNL-DGVFGFAYSS-LS--KHH--PQTT--------AMD-VL-YN--GGVIPNNEIGLQLCPYEM---------
TSNSFINIGNTDVTAK-----------CG-TDGTSVAWVQSPS--------DDQFTVNIKSILV--NDEPVDL-------PEEFQKTIMENGRILYS----SVETCFLHMRFPEVVVDVLINAILDSGAITVN--
IN------I--FSDK-LGKVMVKKIFSQNYAMLESGFDIDW--GKLPT--LSITMFAQN-PVTDANRNSVVTIKLGPKDY----------IQRV---D---
SKNCKYLTIVCIRFAIKAGSNDK----------------AVLGIPFMTQLGLTFDLQ--NKRIGFGA-
>BATDEDRAFT_92488   --KRGEPV----SGSYGSGA-
TWKGYGFLATVNIP-DTGIIATNSPTVGIYQQSIEPTVISGEPRQGVFGFAYPS-LS--YYH-TSPES--------VMD-AW-YK--SKTILKNQVATQLCPYGL---------
SEKSYIDIGNTDSTKK-----------CG-TNGESVAWVYSPS--------LDFFTVSIRNILV--NDRPVIL-------PVAFKENEF--QQNSFS----
YIDTCSRSIRLPHLVLDGLIGAITASRAFQPP------------------LTTMETLAFFWQRDVIDTLKYTIDW--KKLPT--LSFVLDTGV-PKKKKGSYSTVKITLGPKDY----------LQQI---G---
PNQ--------YLFSVGFGSETS----------------ITLGIPFMTRLKVVFDRE--GGRIGFG--
>BATDEDRAFT_35816   --VGTVQIGTPPISVTVQF--DTGSASFWITSSLCKAGNACPPTSPVYNPD----------------------TSTTAKTINNTLL----NRDYGDGT-
SVKCLVISDTLVIA-GVAIPNQS---ICSATAINYPSMSSE---DGLIGLAPPH-----YSN--DPAD--------VFS-NL-----
KNVFPQNKVNFYYNRNVNSLESNTGLVPNAGEITFGAPDPNRF----------------TGNFKWMPIVT-------TESHWAVAFDSVTI--NGRTTSA------------------SKISA----
LFDTGTSLIYLNQPIFNAVNSAMGGVKIRNSD--------------------------------------IYQVDC--TKVKS--FPPVTFTFN----------GNSFTLNWDQQ----------VIVL---Q---
GQT--------CISLFSASGDLP----------------TIFGASFLRQFYTSFDYT---GLVGL---
>BATDEDRAFT_92765   ---SGEPV----TIDY-KGD-
EYNGFSSNAAVTIP-GTSITDINLPVISVQKQSPDLVGINPNL-DGIFGFAYTS-LS--NHH--PQTT--------AMD-VL-YT--SGVIPNNEIGLQLCPYEM---------
TSNSFINIGNTDVTAK-----------CG-TDGTSIAWVQSPS--------DNHFAINIKSILV--NDEKVNL-------PEEFQKKV-ENGRTLYS----SVETCFLHMRFPEVVVATLINAILDSGAITVN--
IN------I--FSDK-LGKVMVKKIFSQNYAMLESGFDIDW--GKLPT--LSITMFAQN-PVTDANLNSVVTIKLGPKDY----------IQRV---D---SKN--------
FRFAIKAGPNDN----------------AILGIPFMAQLGLTFDRA--HKRIGFG--
>BATDEDRAFT_90625   ---SGEPV----TIKY-KGD-
DYNGVSSDAVVTIP-GTRITDINLPVVAVEKQSPDIVGISSKF-DGVFGFAYTL-LS--KHH--PPAT--------AMD-VL-YN--HGVIPNNEIGLQLCPYDI---------
LQESFINIGNTDVTEK-----------CG-TDGTSVAWVNSPT--------NDHHTVNIKSILV--NGKRVEL-------PDEFQKKV-EHERTLYS----VVETCCTYMHFPRVVVETLIDAIVGSGAITIK--
NN------M--LRRE-FNQEDVDDIFWKHRLMIRSNLDINW--GRMPS--LSITMFAQT-PVTVENRNSVVTIKLGPKNY----------IQRV---D---SKH--------
VVFAVQAGSNEK----------------AILGMSFMTRLGLTFDRT--HKRIGFG--
>BATDEDRAFT_23759   ---SGKPV----TIKY-RGK-
EYNGISSTAVVKIS-GTRITGINLPAILVEKKSPGLVGIGGNPDQGVFGIGYSS-LS--KHH--TSVP--------AID-AL-YN--GGVITNNEVGLELCPYKM---------
LSKSSINIGNMEVTAK-----------CG-TDGTSVAWVDSPS--------NEQHSVNIKDILV--NGEKVDL-------PKEFQKTIIEHGRIFYS----YLQTCFVYMYFPRVVVETLVAAILDSGAILVK--
KT------KSEHKHK-LSETEIKKIFWENCLMFKSNYNIKW--NKLPS--LTIVMFAEN-PVTDANRNSVVTIKLGPKDY----------IQRV---D---
SKHGKYLTIVCIRFAVKAGSNDN----------------AALGIPFMNRLRLVFDRQ--NKRIGLG--
>BATDEDRAFT_22623   ---NGQPF----TIKY-KNE-
EYNGFSSYADVTIP-GTSITDINLPVLAVQKQSPDLVGIGGRIDQGVFGFAYPS-LS--KHH--PPAT--------AMD-VL-YN--HDVIPRNEIGLQICPYGM---------
LQESFINIGNTDVAAK-----------CG-TDGSSVAWVESPP--------DGYFTVNIKSILV--NGKQVDL-------PEEFQQVV-ENGRTLYS----YLHTCFTYIRFPKTVVDVLINAILDNNAITTK--
GN---------MLRRKLSKIMVKSTLKNNYLMSQSDFNIDW--SKLPT--LSIVMFAQT-PVTDDNSNSVVTITLGPRDY----------IQRY---D---SKK--------
FLFTVEVGSNDN----------------VVLGASFMTRLGLTFDRQ--NVRIGFG--
>BATDEDRAFT_88273   ---NGQPV----AIEY-KGE-
EYRGVSSNADVTIP-GTRITGINLPVIAVEKQSADIVGIHPRF-DGIFGFGYTS-LS--KHH--SPAT--------AMD-IL-YT--NDVIPNNEIGLQLCPYGM---------
LSDSFINIGNTDVTAK-----------CG-TDGRSVAWVDSPS--------DDQFSINIKSILV--NDEPVEL-------PAEFQKKV-ENGRTLYS----SVETCSAYIHLPETVVATLINAILDSGAITSK--
SI------L--FKNK-LGKIMVKINLQGNFLISKFDFKIDW--DKMPS--LTITMHAET-PVTDDNRNSVVAIKLGPKDY----------IQRY---D---SEDCKYLT---
IMFIVTAGSNDY----------------AILGRSFMTRLGLTFDRA--HKRIGFG--
>BATDEDRAFT_28777   --YTPSGQPV----TINY-KGK-
EYRGISSDAAVTIP-GTGITDIDLPVLAIQKQSPDLVGIGGKLKQGVFGFAYFS-LS--KHH--PPTT--------AMD-IL-YN--YGVIPNNEIGIQLCPYEM---------
TSKSFINIGNTDVAEK-----------CG-TDGRSIAWVNSPT--------NDYFTVNIKSVLI--NDKQVDL-------PEEFQQVV-ENGRALYS----YLHTCFMYMRFPQAVVDVLINDILNSGAITIK--
NT---------MISSKLGKIIIKKKLQNNHLMTKSKYNIDW--VKLPT--ITITVFAQT-PVTDDNHDSVVTIKLGPKDY----------LRSY---N---SKD--------
YLFAVQAGPNDN----------------AVLGTSFMARLGLTFDRT--NKRIGFG--
>BATDEDRAFT_26088   ---SGEPV----TIGY-KGK-
QYNGVTSTTTVTIP-GTSITDINLPVISVEKQSADSVGINPNF-DGVFGFAYSS-LS--KHH--PQTT--------AMD-IL-YN--HDVIPNNEIGLQLCPYEM---------
TSNSFINIGNTDITAK-----------CG-TDGTSIAWVDSPS--------DDQFTVNIKSILV--NDEPVDL-------PEEFQKTIMENGRTLYS----SVETCFLHMRFPEVVVSTLVNAILDSGAITVN--
IN------I--FSDK-LGKVMVKKIFSQNYAMLESGFDIDW--GKLPT--LSITMHAQT-PVTDANRNSVVTIKLGSKDY----------IQRV---D---SER--------
FRFAIKAGSNDN----------------AILGIPFMAQLGLTFDLQ--NKRIGFAP-
>BATDEDRAFT_86720   ---SGEPF----IIKY-RDK-
QYKGVTSTAAVTIP-GTGITDIKLPVIAVEKQSADSVGIDPEF-DGIFGFAYSS-LS--KYH--TSVP--------AMD-VL-YN--GNTIPNNEIGIQLCPYDM---------
ISNSFINIGNTDITAK-----------CG-TDGRSVAWVNSPS--------DDRHTVNIKNILV--NGKQVDL-------PVEFQKKE-ENGRTLYS----VIQTCCTFMHFPRTVVTALVGAIVGSNGITVK--
NT---KFKSKSKHKRKLTPEEIEKIFWEHHLMSRSNFDINW--SKLPS--LTIVMYAQT-PVTDDNRNSVIEITLGPKDY----------IQKI---N---SEK--------
FVFAVTAGPNDY----------------AVLGISFMTRLAVTFDLQ--NKRIGFAP-
>BATDEDRAFT_92592   ---GIVQIGTPSQSFLFQFLSDTGSSLFWVRSSECTNAITCSGLP----------------------------QFNSSASSSGSQT---QQIEYGDGS-
KVSCQMYSDTVILA-GIQISNQK---FCAAT-SIYLPGNLPTI-DGIVGMAPAS------FN--EPSN--------IFQ-HL-----
QAQLPNSQVGFWFNRSVTTLAAGSAVSRGAGIITFGGLNSDLY----------------SGSIAWIPTIP-------DLTRWIVPFKGIFI---GDNMRI-------MDSFLKVQ-----NLSA----

```
IFDTGTTFVILPQVIFDPLNSIMGGIDQNNT---GT----------------------------------YILDC--RSVSN--LPTLTISFI---------EGVTHTLTWDKQ----------ILIV---Q----
NQ--------CISIFTRNNNGAVDTGELNRTVTVPP--AIIGASFLRHFYTCFDYN--AKRIGLAT-
>BATDEDRAFT_22618       ----------------------------------------------------------------------------------NGEPV----TIDY-KGN-
EYRGISSDAVVTIP-GTRITDIKLPVLAVRKQSPDLVGIDPEF-DGIFGFGNSL-LS--NHH--SRIT--------AMD-AL-YN--GGIIPNNEVSLQLCPYEM---------
TSNSFINIGNTDVTAK-----------CG-TDGRSVAWVDSPP--------DGRHTVNIKSILI--NDKPVNL-------PEEFQKRV-KDGRTLYS----VIQTCFRYIRFPEVVVKALIDAILDSGAITVK--
KA------M--YSNK-LNSEEIKRIFWEHYAMLESKLNIDW--GKLPT--LSITMFAQN-PVTDSNRNSVVTIKLGPRDY----------IQRY---D---SER--------
FVFIVAAGSNDN----------------VILGIPFMTRLGLTFDRA--HKRIGFG--
>BATDEDRAFT_28513       ----------------------------------------------------------------------------------SGEPV----TINY-RGK-
QYRGVTSTAVVTIP-GTRITDIKLPVLAVQKQSPDLVGIDPEF-DGIFGFGYTS-LS--KHH--PQIT--------AMN-TL-YN--DGVISNNEIGIQLCPYDI---------
LHESFINIGNTDVTAK-----------CG-TDGSSVAWVNSPT--------NDRHTVNIKNILI--NGEEVEL-------PAEFQKKV-ENGRTLYS----VIQTCSTFMYFPRVVVKALIDAILDSNAITVK--
KT------V--FSNK-LNKKEVKRIFWEHYAMLESGFNIDW--NKLPS--FTIVMFAQN-PVTDDNRNSVVKITLGPKDY----------IQRY---D---SEK--------
FWFTVTAGSNDY----------------AALGIPLMTHLAVTFDLQ--NKRTGFG--
>BATDEDRAFT_25617       -FYIDLLIGTPPVNVNVLI--DTGSSTFWIDDSACFDSRQCKTHKYNP--------------------------IDSTTQRPVVHPI---VHREYGDGT-
SVTCTIISDVVSIA-GISIPNQT---ICAASQVKSIESISL---DGILGLGPPN------SV--DPAD--------LFS-NL-----ENSLSERKVSFYYDRTVT-
PINPQLQIRDAGEVTFGVPNPARY----------------TGNFSWSPIVK-------TDSHWALTLSSVVI--DGKTFSS-------------------TPIVA----
LMDTGTTIIILDKGTFNAINSAMGGVSVQGVL--------------------------------------YELDC--TMVSK--MPSIQFNFQ----------GVTLTMTWDQQ----------YFVI---E---
NQY--------CVSVFGYSANLPPNM-------------AIFGAWFLRSFYTSFDYT--GRQIGFAT-
>BATDEDRAFT_27277       -------------------------------------------------------------------------------------LV----TINY-RDK-
EYSGFSSDTDVTIP-GTSITGINLPVIAVERQSADSVGISSNL-DGVFGFGYSS-FS--KHH--PPIT--------AMD-IL-YK--DGIIPNNEIGLQLCPYDI---------
LHESFINIGNTDITAK-----------CG-TDGSSVAWVQSPT--------NDQFTINIKSILI--NDEQVEL-------PAEFQKRV-EDGHTLYS----FIQTCSAYMYLPKTVVATLINDILDSGAIRVK--
GI------L--FRNK-LGKIMLKSILQGNYIMPKSEFKIKW--EKLPS--FTIVMFAQN-PVTVNNYNSVVTIKLGPRDY----------IQKT---D---SDE--------
FLFTVTAGPNNN----------------AALGIPFMTRLEVTFDRQ--NQRIGFG--
>BATDEDRAFT_26741       ----------------------------------------------------------------------------------SGNPV----SIKY-KGK-
EYRGVTSTAVVTIP-GTRITDISLPVIASERQSADSVGIDPEF-DGMFGFAYPS-LS--KHH--SRIT--------AMD-AL-YS--NDAIPNNEVSIQLCPYEM---------
LSDSFINIGNTDITPK-----------CG-TNGRSVAWVDSPP--------DGRHTVNIKSILI--NGKQVDL-------PAEFQKNQGKDGRTLYS----VIQTCFTFIHFPKVVVKALVDAIVGSNAITVK--
KT------M--FSNK-INSEDIDEIFWKHYAMIESKYDIDW--SRLPS--LTIKMFAQT-PVTDDNYNSVVTIKLGSKDY----------MQNI---N---SKH--------
VWFTVRVGSDDY----------------SILGMSFMNRLAVTFDLQ--NKRIGFG--
>BATDEDRAFT_24300       --------------------------------------------------------------------------------TSNGQPV----TIYY-KGE-
EYNGVTSTATVTIP-GTSITGANLPVLAVEKQSEDLVGIHPEF-DGVFGFAYSS-FS--KRR--SPAT--------AMD-AL-YK--DGNIPKNEVGLQLCPYGM---------
LSDSFINIGNTKVTAK-----------CG-TDGRSIAWVRSPS--------NDQFSINIKSILV--NEKPVEL-------PAEFQKRV-KDGRTLYS----VIQTCLTYMYFPRVVVDTLVDAIVDSGAITVK--
KI------M--LSNK-LNSREIEKIFLKNYAMLESGFSIDW--NMMPT--LTITMFAEK-PVTVENSNSVVTIELGPKDY----------MQRY---D---SKN--------
LVFAVKAGSNDY----------------AVLGIPFMNRLGLTFDLQ--NARIGFG--
>BATDEDRAFT_25148       ----------------------------------------------------------------------------------SGNPV----TIKY-KLK-
EYNGFSSTATVTIP-GTRITGVNLPVIEIEKQSASLVGIDGTLEQGVFGFGYSL-LS--KHH--TSVP--------AID-AL-YN--DGVIPNNEIGIQLCPYDM---------
LQESFINIGNTDITAK-----------CG-TNGKSVAWVQSQS--------DGYFSVNIKSILV--NGKRVDL-------PAEFQKKV-ENGHALYS----YLHTCLTYMHFPKTVVTALVDAIVGSGAITIK--
RI------K--LKRK-LYPEEIDDVFLENSLMSQSDYNIDW--SKLPS--FTIVMFAET-PVTDDNRNSVVTITLGPRDY----------IQKI---N---SED--------
FVFAVGVGPNDY----------------AVLGASFMTRLGLTFDRA--HKRIGFG--
>BATDEDRAFT_25223       --------------GTFNVMVDTGSSDTVIPVKNLNN-------------------------------------YNGPTLDYMGQPNAKMISGSYASKA-
GWTGYGLSLFVGLS-GTDTTADNAPIVNAIRQTANPIFIDGQDTQGLLGLAYSA-AA--VYK-TNPST--------VMD-AW-VA--AGQFAKNEVAFRACPWEK---------
ESQSSIDFGNTQPVNN-----------CG-KNGSPNVWVKSPE--------QGLFSVDIKGISV--HGTPVRL-------PSAFQ----RNGR--LS----IIDSCTTDLKLPGNVINAIRDIMIRLGGFPPL--
NT-------------------------ARTPAPPTR-PINL--ALLPS--ITFHI-ATG-GAMRSAPGSTISVTIGPQQY----------LIYR---P---G----------
YWYQMAAFRSDDDS--------------VILGGPFFTSLNIVHDRT--NHQIGLS--
>BATDEDRAFT_27292       ---------------------------------------------------------------------------------SSGKPV----TIRY-KSR-
KYKGFSFDTDVTIP-GTRITGINLPVIAVKKQSASLVGIGGNPDQGVFGIGYSS-LS--DHH--SRVT--------AID-AL-YN--GGVIPNNEIGLQLCPYKM---------
LSKSSINIGNMDVTPK-----------CG-TDGKSVAWVDSPT--------NDQFTVNIKSILV--NGEKVDL-------PKEFQKHILKNGHTLYS----YLQTCFVYMYFPRVVVETLVAAIVGSDAITVK--
KT------KSKHKHK-LSKTEIKKIFWENRLILKSKLSINW--DRMPT--ISITMFAEN-PVTDDNRNSVVTIKLGPRDY----------LQRV---N---SKE--------
FRFIVEAGPNEN----------------AALGIPFMNRLRLVFDRQ--NKRIGLG--
>BATDEDRAFT_23275       ---------------------------------------------------------------------------------SSGKPV----TINY-KSS-
DYNGITSTVSVTIP-GTRITGINLPVLSVKKQSADSVGIHPKF-DGVFGLGYTS-LS--KHH--TSVP--------AID-AL-YN--GGVIPNNEVSIQLCPYGM---------
LSDSFINIGNTDVTAK-----------CG-TDGRSVAWVRSPS--------DDHFFINIKSILV--DDEEVEL-------PEEFQKKERKNGHTLYS----IIETCFTYMRFPETVVDTLADAILDSDAITVK--
KT------KSEHKRK-LSETEIDEIFLENSLMPKSTYNIDW--GRLPS--LTITMFAEN-PVTDDNRNSVVTIKLGPRDY----------IQRY---D---SKN--------
FMFTVQAGSDDN----------------AILGIPFMTQLGLTFDRT--HKRVGFG--
>BATDEDRAFT_26425       ------------------------------------------------------------------------------ESTPSGEPV----TINY-RRK-
QYKGLTSTAAVTIP-GTRITDINLPVLAVEKQSPDIVGISSKF-DGVFGFGHPY-LL--NRH--SRIT--------AMD-VL-YT--NNVIPNNEVSLQLCPYDI---------
LHESSINIGNTDITAK-----------CG-TDGSSVAWVESPT--------NNQFTVNIKSILV--NGEQVDL-------PDEFQKRV-ENGHTLYS----VIQTCFRYMVFPETVVAALIDAIVGSNAITIK--
NI------L--FRNR-LGKIAVKNILQKNHPMSKFEFKIKW--EKLPS--LSITMFAKN-PITDENHDSVVTIKLGPRDY----------LQRV---D---SDE--------
FEFIVAAGPNDN----------------VILGIPFMNRLAVTFDRT--HKRIGFG--
>BATDEDRAFT_89959       ----------------------------------------------------------------------------------SGEPV----TIGY-KGK-
EYKGVTSTAVVTIP-GTRITDISLPILAVRKQSADLVGISSNF-DGVFGFAYSS-LS--KHH--PPAT--------AMD-VL-YT--SDVIPSNEIGIQLCPYEM---------
ISDSFINIGNTDVTAK-----------CG-TNGRSVAWVQSPS--------DDYFTVNIKSILV--NGKRVEL-------PAGFQKKV-EHGRTLYS----VMETCFTYMQFPKVVVAALVDAIVDSNAIAID--
IF------N--FKYR-LNNLQIENLYWKNHLMPEKYFSINW--GKLPS--LSIVMHAET-PVTDDNYNSVVTIRLGPRDY----------IQRV---D---SKN--------
FVFAIKAGLNDK----------------ATLGMSFMSRLGLTFDRA--HTRIGFG--
>BATDEDRAFT_90577       ----------------------------------------------------------------------------------SGQPV----TINY-KGK-
EYRGISSDAAVTIP-GTGITDIDLPVLAIQKQSPDLVGIGGKLKQGVFGFAYFS-LS--KHH--PPTT--------AMD-IL-YN--YGVIPNNEIGIQLCPYEM---------
TSKSFINIGNTDVAEK-----------CG-TDGRSIAWVNSPT--------NDYFTVNIKSVLV--NGKQVDL-------PEEFQQVV-ENGRALYS----YLHTCFMYMRFPQAVVDVLINDILNSGAITIK--
NT---------MISSKLGKIIIKKKLQNNHLMTKSKYNIDW--VKLPT--ITITVFAQT-PVTDDNHDSVVTIKLGPKDY----------LRSY---N---SKD--------
FLFAVQAGPNDN----------------AVLGTSFMARLIVTFDRT--HKRIGFG--
>BATDEDRAFT_24380       ----------------------------------------------------------------------------------SGEPF----IISY-KGK-
QYKGVTSTAAVTIP-GTGITDINLPVISVEKQSPDSVGISPNL-DGVFGFAYSS-LS--KHH--TSVP--------AMD-VL-YN--KDVIPNTEIGLQLCQYDM---------
LHESSINIGNTDITAK-----------CG-TDGRSVAWVQSPS--------YDHHTVNIKSILV--NGEQVEL-------PAGFQKKV-EHGRTLYS----YLHTCCTFMHFPRVVVEALVNAIVASNGITVK--
NT------KSKSKRK-LNKEEIKRIFWEHHAMPRSNFHIDW--SKLPS--FTIVMFAQT-PVTDDNSNSVVKITLGSKDY----------MQKI---D---SEK--------
FVFAVGVGSNDY----------------AVLGIPFMTHLAVTFDLQ--NKRTGFG--
>BATDEDRAFT_27300       ----------------------------------------------------------------------------------SGRHV----TINY-KGK-
EYRGIDFTATVTIP-GTSITGIGLPVVAVKKQSPDSVGISPNF-DGVFGIGYPS-LS--KRR--PQTT--------VLD-VL-YN--DGVISNNEIGLQLCPYGM---------
RSKSFINIGNTDVTPK-----------CG-TDGKSVAWVQSPS--------DDRHTVNIKSILI--DGKQVNL-------PEEFQKRV-KDGRILYS----YIHTCFVYMYFPEVVVAALVDAIVGSNAITVK--
KT------KSEHKHK-LSETEIEKVFWENRLILKSKLNIDW--SKLPS--LSITMFAQN-PVTDENSNSVVTIKLGPKDY----------IQRV---N---SKE--------
FRFTVTAGSNEN----------------AALGISFMTRLAVTFDLQ--NARIGFG--
>BATDEDRAFT_26758       ----------------------------------------------------------------------------------SGEPV----SIKY-KGD-
EYNGVTSTAVVTIP-GTGITGINLPVVAVERQSADIVGIGGTLKQGVFGIGYHS-FP--NQH--SRVT--------AMD-AL-YN--GGAIPNNEVSLQLCQYDM---------
LHESFINIGNTDITAK-----------CE-TDGTSVAWVESPS--------NNQFSVNIKSVLV--NGRPAEL-------PDEFQKKQGKDGRTLYS----RVETCFLYALFPETVVAALVDAILNSNAITSK--
NI---------LLRRKLDKEEIKKKLQANSPMFKSNYNINW--GKLPT--LTITMFAQT-PVTDENRNSVVTIKLGSKDY----------MWRY---D---SEK--------
FRFAVKAGSKDH----------------AVLGISFMTQLAVTFDRA--HKRIGFG--
>SPPG_01941T0   QYYGEITLGTPPQTFSVVL--DTGSSNLWVPSTHCSSIACWLHRRFDS--------------------------SKSSTFKPNGTEF----AIQYGSGS--LEGIISNDVLGVA-
DLLVKDID---FGESVKEPGITFAVGRF-DGIFGLAYDN-IA---VK--RVVP--------PFY-QM-IQ--QGLLDEPVFGAWFGDQSN--------GGEGGEITFGGIDKDHY----------------
KGEITWAPVIR--------KGYWEVELQNATL--GGQDIGV-------------------RTKRA----
AIDTGTSLCALPVEEADAINTRIGAKKNFNGQ--------------------------------------YVVDC--DKIST--LPALTLKFG----------DKEFTLEGKDY----------
VLQVSGGPIGGGNQ--------CVSGFMGLDIPAPAGPL-----------WIVGDVFLRKYYTVYDLG--KNRVGFADA
>SPPG_00681T0   -YQGAVEIGTPPQPMSVLF--DTGSSQLWVQTTTTTGDINRNGRRFDP--------------------------AKSSTFQATSQQA---DPIEYVDGT-SVQGIFVKDTVAIN-
TLSVPNLQ---FEAATSIKSPNQNTSDM-DGIMGMSFSLPTT-------AGGT--------ASPTWW-----ERVVSANQVTSPVFGYYI------DSTNQNGGLTLGGVDAARF---------------TGGLT-
WLPVAGSGPNGKEPFVFWQSLMTAVSFPGSSRTISL-------PSNFA-----------T----VFDTGTSLAVLP-VQTADQINSMLNLQRVSNS-----------------------------PPFLYA---
TACPG--GKIPS-GFPDIKFEFT---------GGHFVTISPQDY----------LFMQPT-DIAGVTA--------CVSGFAGQDIHGGSTTSRPGQAQPLLPSAIFGNVFLRAFYTVFDSA--NKQIGVAAA
>SPPG_07923T0   --ITTIGVGTPAQVMAVQI--DTGSSLFWLRSSKCRGTSCSGQPVYDNTKSTSYL-------------------PPAPATALTGPQR---RSIVYGDNT-KVECQLGADTVMLG-
TLVIPNQT---LCEADVIQTTTGSS----DGLIGLGPPG------AD--TATD--------VFR-NM-IA--SKTISKPVASFWYNQSRQ------FSDTNAGQITFGGMDSSKF----------------
TGSFTWFPIPI------TGRNHWSTTFEGITL--DSAPLTQ------------------NRTGMA----IFDTGTTLAIIPSDTLSAIVLAA-NAKQLPNQ--
SL----------------------------------YEIPC--DKVSR--IPPVTFHIG-Q---------RRITLFAEQQ----------VFVV---D----GT--------
CFLIYAASTSNLNVD-------------VILGALFLRHFYTVFDFG--ASRTGLA--
>SPPG_08789T0   -YYGPVTIGSQTFQVDL----DTGSSDFWLRGANCQSSDGSCGNGQPA--------------------------VSTREFKDTGSQF----QDSYGSGS--ADGEIYQGTYTLG-
```

```
GVTAKNAYFGVSTNEQGFTDPA-------DGLLGLGFAA-IS---NI--ASAT-------------------NGRAPIDELGLKSFGFYL----SNANDGDQGLVTLQGADSSKY----------------
TGGFNYVPLNS--------RTYWQYSL-------SGGSVQV----------------
GNTKTAFSIRNAIADTGTTLIIIGDKEASAIARAVGADSN------------------------------------------GNISC--SVART--GPKVVFNIN----------GVAYAIPPSVY----------
VFED---Q----GQ--------CILGIQGGAAQAGV-------------AIFGDVFIRQYYTLFDVA--NSRVGFAL-
>SPPG_08444T0   ----RIDVGTPPRPYGVAL--DSSSADLWLASPDCKTCVGYEPYVA----------------------------SKSSTGKVSDKKF----VVAYNEGSLRVDVTGVTDDLIFG-
GFPIKQQP---FAQADKIPK--GFQDVPFDGVLGLAFSA-HS--EQK--APP---------LLQ-SL-VD--QKVIKSASFALHIPAGKK-----------QATMTIGGIDPTKF----------------
SGEIDFVPVDN-------AAGQWGLNIDQIVA--QGKAVDV-------------------GTKKG----ILDTGTSVLLGPMEDVRKVFAAIPGSAEMANG--
T-----------------------------------FTVPC-----------------------NQDNSFALKLGGKDYGLNGVDFLDEIFSVAGPN---NAN--------
CVAALSGDQKIKS---------------WVIGTPFLQKFYTVWDLS--VPRFGLAK-
>SPPG_04035T0   -----IDVGTPPQSFKAIV--DTGSYDAFLKAKECLDTGCVKDTTYDGS-------------------------KSSTFTTTTGPSV----TLAYADGT-AVTGVASRDTTTVG-
GVTMSNFTFLRVLSYKQASTNVITY----TAMLGMGMPS------SN--ATAGTPGRLSGSSLGRSV-----TGQLSDAGIKPIFGLYPT-------LDGKPGLLTLGGIDTTKF----------------
SGDLTWVDVPT---------FANSSAIDPVMAAGTAGMWKAVMGAVKYPDGTENQV----AGLPI----ILDTGSSLSLFPQSFVTKLGQSIGGTLLAQAG------------------
ASGLQI--------------YKVSC--DKIKT--LQDLTLTLG----------GKTVSIPPSIY----------IQNSFIRG---ETG--------CVLGFVSLGTEGALGGA-----------
ALMGMNILRAFYTAWNWG--DRQVGFAR-
>SPPG_02558T0   AYFGQVKLGTPAQTFTAMF--DTGSYQFWVRSKLCTVQACQGHPKYDG--------------------------DLSSSYVSTNTKA---DLITYADGT-KVQGVYAKEKVSVG-
SVAVQGLTFEEVTDTNESLGAAY------DSIMGMCWPP-VD-------KPKS--------WFQ-TL-VQ--NGGVTSPVMGWVIDSTNT-----------DGSITLGGVDSARF----------------
TGTLQWVPSFG--FASNGGGGGPFIYFQGLTV---AGSVTM-------GNTTTPLSWSQGHFLS-----
VFDTGTSMSVVPTKVAADIHAQFPAFSIDPSRPGGK----------------------------------YYTGW--CDLNT--LGNVTLSFV------GEEGLVSLTLEPHEW----------
VLYIPKNNQPNY----------CLSIFVGNDAVAQKVTVNGPLIA-----GILGNSLLRKFYTVFDWG--KNRTGFAL-
>SPPG_00268T0   -YYTSILVGTPGKWFSVVI-----DTGLWPPSPPVHK-------------------------------------EEGTTQRNQGQLT----SLAF-RSL-PILGSASGSVVT---
DYVRIGNLIATSQVFIQADQMNNVQPANVDGLLGLSFSS-LS--WAN--SVVPDNLVGKSSIIE-NL-YY--GRKLQQPAFGVWLDKYVS--WSAAPGSTVGGELALGSTTGNPA--------------
RYTGPITWLSVPN-------TANWWHVQLNGIA---GPDGVNL---------------VPSGRAIRG----IVDTGTTLIVVDYAVAARL-NGLLGAYGAGVR--
GL----------------------------------WAVSC--NKAKNSGVKITFTLQG-----------NKFTLDAADL----------PTRVWPDD---PNT--------
CYAPFQARQNQDVTDK------------WILGEVFLRKYYQIYDYN--VQS------
>SPPG_05983T0   -FIGQVEIGTPPQKLTVRF--DTATQLSWVKKAAYKEKPKKYDDYNSYDKYDEYKKDNDYYEPERELVYFGFDPDKSSTWQDGGREG----DLEY-YGQEKVIVKEGRDVVNVG-
GIEAT------VTFGLRTQNEVSANETV-DGHFGLSLRN----------PDLD---------FDPWL-YQAVKDGVCDPIFALDPPPVEPPKAYAPQPTPSYGKRDQSYDYYEPPKPKPTTAQLSICG-
YEGQMPTRFNHVT---QRGLQYGYWQLNLKKIKV--GQSLVPLVKESREDPYGYQSYE-PKQKEIDA----LIDTGSPFIMLPKIAVDK-IHKLIGATCPGQE--
YGYSEYKDDYKDDYKKDYKDEKSTHSRRATSMNANSTSGFW--RSQTG--IKSHDLCTV-PCASVPKLANITLTFDQGDY-----------------PILPAEY------------
IQKDPDDY-------------------GKQQCYSVFTTFK-------------
>Hp_gm_asm116-0.104      QYYGEITLGTPPQTFSVVF--DTGSSNLWVPSTRCSSIACWLHRRYDA--------------------------SKSSSYVANGTEF----AIQYGTGS--
LEGVISEDTLSLSDSLTIVGQN---FGESVVEPGITFAVGRF-DGIFGLGYDT-IA--VQRVVPPFY------------QM-VN--QKLLDEPLFGVWLGSDAS-----
GSEGSEGGEISFGAVNHDHF----------------KGPITWAPVVR--------KAYWEVALQSVTL--GGKPLAI-------------------KSSSA----
AIDTGSSLFALPTAEADAINKAIGGKKGWNGQ--------------------------------------YTIDC--AKLDS--LPDLGLQFG----------NKTFTLTGNDY----------
VLKVSGGPIGGGEQ--------CVSGFVGLEIPAPIGPI-----------WIVGDVFLRKFYTIYDLA--RNRVAI---
>Hp_gm_asm242-0.207      --------------------------------------------------------------------------TAVNTNVVN---PTISYGDGT-
TVDCTVYTDSLTFG-DLSVKTQK---VCVATRFFATSASRTMN-AGILGLGNTL--N--DPA--NVAD--------GVS-SI-----LSSFTDKIVSFYYDRNVV-
VNLLGTETKNAGEVTFGTPNSARF----------------KGSLSWAPINK-------IADFWTLPITGASV--GSYSYTY------------------TRTSYV----
VMDTGSSLITIDQPVADGINLALGFTYAGSGL--------------------------------------YTIDC--TKLKS-SLPTITITLG----------GLTFNFGAAA-----------IAVVDPTT----
NT--------CISGVTAPTSANSP--------------RIFGVAFLRNFLVAFDYSS-GGRIGFAP-
>Hp_gm_asm117-0.23       -----------------------------------------------------------------------------
TLSPE----------------------------------------------------------DGLIGLGPPQ-SV---SS--EPAD--------LFA-NL-----
RSSFNQSKISFFYNRAVNSIDGSSEVVPNAGEITFGDPNPARF----------------TGSFTWIPINP-------SDPHWAVTIDSITV--NGQSMSF------------------GQTA------
LMDTGTTLIYITNPMFATINAQM---NGIATN--GM----------------------------------YQVDC--TKVRS--FPPITFSMG-G---------ASFTLTWDKQ----------VVVL---Q---
NRF--------CVSVFSEALDGLP---------------SILGVSFLRHFYTSFDFSG-QGRIGIAA-
>Hp_gm_ctg07303-0.1      ----------------------------------------------------------------------------------------------------------
------------------------VNSTE-DGIIGLGPPG-GV-------STAD--------VFK-NL-----KTAFTESKISFYHDRKVE--DAGGVFAAGGGEITFGVPNPNRY----------------
TGSFTWFPIDP------TNQHSWTAMLSSVSI--GGASVDV------------------GSPI------LIDTGAATNNLDANTV-AFINKQLGATQVAGQ--
RF----------------------------------PQVPC--DKVSS--LPPVVFKFG-P----TPNTAVSFSASWSSF-----------IVYNPAD----KN--------
CYSTLNNAPGPASNK-------------TVIGAVFMRHFYTSYDYD--NRMIGFA--
>Hp_gm_ctg01303-0.7      ---ATVQLGSQQTSLDVLL--DTSSTLFWHSPLTLN------TRLFPF------ILPPWMLSLDTNNSPQISRLPLSSNGKITGFRN---QSITYPDGS-
VVACSVYTDSLTVG-SVQISSQG---ICVADSYTSLGGNAPYD--GVMGIGNNL--A--DPT--NHAN--------APA-SI-----LNGFVDKKVSFFYNRQST-
SSISNTETSNAGEITFGTPNAARY----------------TGSINWIPIAQ------TGTPAWGLSITTVTA--GAFSATA----------------KAGAAI------
VVDTGYPYFALDTTVAAQI-----------------------------------------------------------------------------------------------------------
-------------------------------------------------------NKALGFS--
>Hp_gm_ctg03169-0.2      -----------------------------------------------------------------------STTAVPLNSQHPI-----VTYSDGT-
RIDCSLYKDSVTLG------DVTLPAATICVTNSYNQTTNPQR-QSVLGITPNI-----TAHPADPAD--------LTA-QI-----LAGYTSKKVSFFNIRSRM-
TVLDTNIVDGAGEVTFGAPNPARY---------------TGTGLSSVILPS-------STVSWQLQFNSITA--GNTSFQF-----------------NGTVKPI----
SFQPSTTFIYLDTNVADSINAALGFTRASNGD--------------------------------------NVVDC--AKLKTTTFPKLYMSVG----------TVLYTIGILNV----------VVNF---Q----
GN--------CISGIYGTTGEN-----------------VLGSVFTRSFYTTFDYD--TNSITTAQ-
>Hp_gm_ctg07229-0.0      ---------------NVLF--DTGSNVFWVSSSSCA------GCTKTA--------Y-----------------NTTASASIKSTSI---LSFGYGDGT-
QVNCSVFTDTLTLS-GVTLPGQN---VCVASSLIDPV-NSP-E-DGIIGLGPPQ--P---NP--SQAD--------VFA-NL-----KTSFAESKISFYHNRQVE--
DTSGDPAPNSGEITFGVPNATRY---------------VGNFF-
W---------------------------------------------------------------------------------------------------------------------------------
-----------------------------------------------------------------------------------------------------------------------------
>Hp_gm_ctg13909-0.3      ---------------TLRVMIDTGSTDLAIPLNGLNAYSG----------------------------------PTVNATRPAGAAN---MTATYGGGN-
QWIGFGFSGTVQLA-NTTIVATNAPTIGAIQQNGPPIMTGPPDSEGLFGVAYPS-LSMYGYN-KNPGT--------VVD-AW-YQ--SGTMAKNQFLFHGCPYDK---------
QSQSFVDFGYTQPVTT-----------CGNTAGTPYVWAKSPQ--------LAWYTLDIRAILV--NGVAATL-------TSAFQN---PTGSMRWS----
IIDSCTSVLMLPPAAFNALTTAI---------------------------------------------------------------------------------------------------------
KN----------------------------------------------------------------
>Hp_gm_asm117-0.25       --LGTIQVGTPPVPLNVLF--DTGSDIFWINSVQCSGGNGCPLDNSTAYDL-----------------------TKSSSAVPLSKSI---PPVVYGDNT-
TIQCSLFTEKVTVG-GITLVSQQ---VCVATQVFNPLNTNE---DGIIGLGVPS----------SGVT---------LA-NL-FASLKLRLPANMASFWYNRNLS--
SSTTDILEHGGEIALGRPDSSRY----------------IGDFAWLPVLP-------KQAHWTVAMNMISV--DDHAMMT-----------------TGSTA------
IIDTGTSITILDGTLASEINTRLGGWPASTTS-------------------------DVF----------YLFDC--ATVQS--LRSVTFTLA----------NFNFTLTWDKL----------FYTTTLNG---
KTF--------CISTLWKSPMPGF---------------NILGCTFLKHFYTAFDFQ--NLRVGFAT-
>Hp_gm_ctg09870-0.5      ---APIQLGSQKSHIDVIL--DTGSNVFWVSAVGCN------GCISPQ------YDT-----------------KKSSTAKTSGNHN---AVLKYGDNT-
VVECAFWNDTLALG-DITVSTEG---VCVATKYTPGSEVAHDF-TGILGIGSSL--E--KST--NLAN--------APA-SI-----LAGFSEKKISFYYDRSVL-
VNLARNQTGKAGEVTFGTPDSTRF----------------TGDIHWSKITK-------ANSFWEVPITSVEV--GTFKHTY----------------DSAQTI------
ILDSGTSLIYLDQDVADAVNTALGFASAGDGF--------------------------------------YTIDC--SKLAS-NNSRTSFSIG----------SVKATLSTIAL----------TVVV---E----
GS--------CFSAIVANPPNSL---------------RVLGAGFLRNFYTSFDYD--TASVGFA--
>Hp_gm_ctg01127-0.11     ---GTLSIGTPAQNLNVQF--DTGSSYLWVSGPQCTDTGCAQSKKFNP--------------------------SASSSFGPAPDPAVASHTYYYGDST-
QVACTVGIDTINLA-GVPFANAT---VCVANDVSGSAGV-----DGMIGLGPIV------GQ--ADTS--------IVA-WI-----KSSFAQPYMSFWYNRAGP------
ANGPNAGEITFGGINPARV--------------NTTQKPAWLPTLP----MTQDTSAWTVSLSAAAV--GSQTVAT-------------------QGTQA----IIDSGSTLIILPRAVFNGL-NAVVQATLDPNL--
GV----------------------------------YTFDC--GKVAT--LPPISFTFA-GSPHP-----YVLSAGQQIY----------YASG--------ST--------
CAWGLTPTDDTL----------------AIFGVVFMRQFYVTFDFTG-PMQIGLS--
>Hp_gm_ctg01127-0.10     ---GYVSIGTPPQEFQVQF--DTGSTVSWVRSTACRNSEGCLPGDISFN-------------------------PTASSTYKAGNST---QSIIYGDGT-
RVNCRIVHDVIAVA-GVQLKSNN---ICIATEITIPTGSSSL--DGLFGLAPPK---------------------------------------QEATLQSDQEED---------
AIAGEITFGGRNPSRF---------------VNDTTIWLPTTV-------NATFWNVAFNSMQY--GNVTLQI----------------TDAMRHSA----
ILDTGTTLLNLPAAIFDIVNKMMGGKLNQYGL--------------------------------------YELDC--RKVTS--LPNVTFSLA----------GHDFQLTYQDL----------VLTD-------
SET--------CVSIFGRTPDTF----------------TVFGSVFLKKYYITFDLA---GSVGFAQ-
>Hp_gm_ctg03150-0.14     ---TSISIGTPPQSLYVVP--DTGSWMLWIKSSQCT---------QCA---PPVYNG-----------------QKSSTYRSQGQIA---SPIQYIDGT-
SILGFYCSDDVTIG-SLSMPNFV---FTEASYV---NRTRSTL-HGVLGLSFP-------SS--QPID--------HFFTRLNQT--SDPASAPVFGYGIEQNGQ-----------
YGWLTVGAIDIGRY----------------SGDLTWYPVVS--------NQQWLLQTTAFFV--GDTQLTS---------------IPNMRM-------
MFDTGTSLTMFPLNVAKALNEAMLLTPILPATDPNN--------------LPQ-----------------YGMPCFDGVIPK-NLPNITFSFQ--------GASQGITITPREY----------MYIG---
SISGNLY--------CFSTIIGNFGDGL---------------AIFGNALLRRFYTVFDVQ--NAKIGVAV-
>Hp_gm_ctg01303-0.6      ---GTLLVGSQKAALDVVL--DTGSTVLWISSAICT------GCLVTT------YDP-----------------RASSTAKQTTLPP---KTINYADKT-
AVTCSYYTDSIGLG-DLSVPSSG---VCVATMFSATSDTRRQN-PGIMGVGWDL--A--DPN--NPAN--------GPA-AL-----LAGFSDKKVSFYYDRSVV-
VTLSGTETPNAGEVTWGAPNPSRY----------------NGSISYTPIIK--TAGSIFSNYWSLPITSLSC--GSYVASL------------------SSSAAV----VMDTGTTAMAI-DSTVAANINTAFG---
```

```
FTLS--SN--------------------------------GDYVIDC--SKLTS-----GTFPQL-KVTLGG----ITFTLGIGNI----------VIVY---N----GV--------
CTSGIVGGPVDT----------------PLFGVVFLRNFHVTFDSA--GGQIGFA--
>Hp_gm_asm375-0.190     ---GSVQLGSQKAALDVVL--DTGSTVFWVSSGICD------GCTFTQ------YNP-----------------NQSATAHSTGIQN---PTMVYGDGT-
TVDCTFFTDSVVFG-DITLPSEG---ICVATKFFAQAGLKNLG--GIMGIGKIL--N--DDN--NRAN--------APP-AL-----LASASEKKVSFYYDRSIE-
IALSGDVRDNVGEVTWGAPNPARY----------------TGQINWTPIMP------TNNYFWTLPLKSITI--GSFSRTF-----------------NDQPI------
IMDSGTSLLLLTDSALVDSINSALGYTVGSGQ-------------------------------------FATIPC--SRI----WDGTVFPKM------------QITLGNVNF----------
QLSIANTMIVLGNT--------CASGIIKGGD------------------NIFGVTFLRNFMTSFDYT--AGAVGFAQ-
>Hp_gm_ctg01383-0.6     ------------------------------------------------------------------------------------------TAFQY--
GTDTLEGTIVSDTVGLADQQSFTSQNIGQCVAS-------FMNSDF-DGIFGLGLPG-SS---LN--GLST--------PLQ-DL-AN--QGLLPANIFGLFLATGDS----------
TASALTLGGTDPSFT----------------SADINWVPLTP-------GSQRWQVTLDSLAV--LPPPSSV--FSQLLNTSSQPTVIASG--MQA----VFDTGTSLIAMP-VQYADAIHALLAPSVLFNN--
RL----------------------------------RLIPC--MNMPS--ISIKLNG-------------IEFILTNADY----------IIPF---G---FGY--------
CVSAFVGLNIENV---------------IVLGDTFLRKYYSVFDVD--GARVGLV--
```
