# Supplementary material for: Genomic Transition to Pathogenicity in Chytrid Fungi
Source: PLoS Pathog. 2011 Nov 3;7(11):e1002338. doi: 10.1371/journal.ppat.1002338 (PMC3207900; doi:10.1371/journal.ppat.1002338)
Supplement: Figure S2 — Maximum likelihood phylogenies of gene families containing (A) M36, (B) S41, and (C) Asp Pfam domains. (PDF) [file ppat.1002338.s002.pdf]

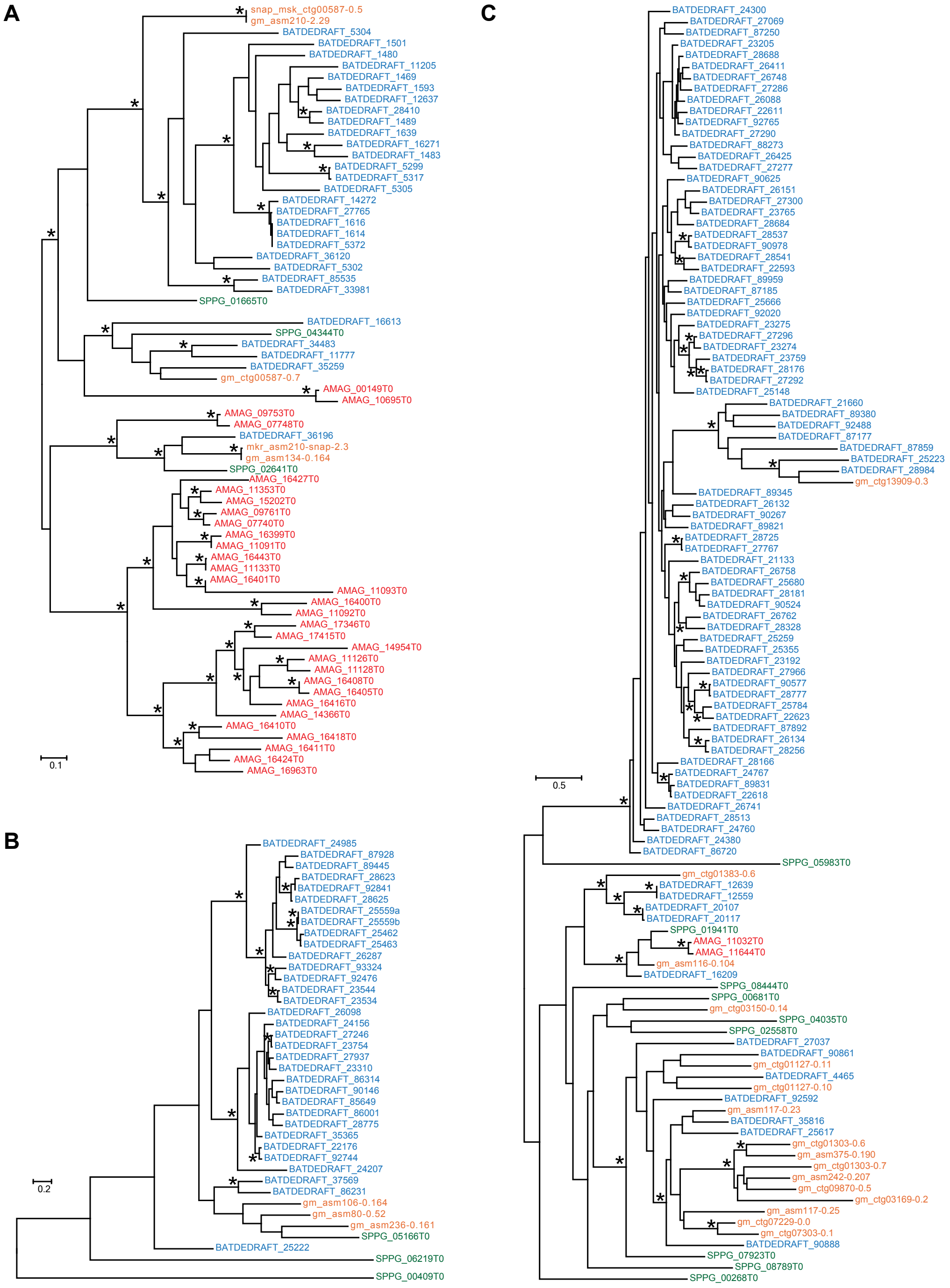

Figure S2. Maximum likelihood phylogenies of gene families containing (A) M36, (B) S41, and (C) Asp Pfam domains. Each tip represents a single gene copy and each source species is denoted by color (*Bd*: blue, *Hp*: orange, *S. punctatus*: green, *A. macrogynus*: red). Bootstrap values over 80% indicated with asterisks at internal nodes.
