# Supplementary material for: Genomic Transition to Pathogenicity in Chytrid Fungi
Source: PLoS Pathog. 2011 Nov 3;7(11):e1002338. doi: 10.1371/journal.ppat.1002338 (PMC3207900; doi:10.1371/journal.ppat.1002338)
Supplement: Table S1 — Proteomes used in this study. (DOC) [file ppat.1002338.s003.doc]

Table S1: Proteomes used in this study.

| Taxon and strain number | GenBank Genome Accession Number | Protein Database Location | Citation |
| --- | --- | --- | --- |
| ASCOMYCOTA |  |  |  |
| *Arthroderma benhamiae* CBS 112371 | ABSU00000000 | www.broadinstitute.org/annotation/genome/dermatophyte_comparative/ | [1] |
| *Aspergillus nidulans* FGSC A4 | AACD00000000 | www.broadinstitute.org/annotation/fungi/fgi/ | [2] |
| *Blastomyces dermatitidis* SLH14081 | ACBU00000000 | www.broadinstitute.org/annotation/genome/dimorphic_fungi/ | *Blastomyces dermatitidis* Sequencing Project, Broad Institute of Harvard and MIT |
| *Botrytis cinerea* (B05.10) | AAID00000000 | www.broadinstitute.org/annotation/genome/botrytis_cinerea.2/ | *Botrytis cinerea* Sequencing Project. Broad Institute of Harvard and MIT |
| *Coccidioides immitis* RS | AAEC00000000 | www.broadinstitute.org/annotation/genome/dimorphic_fungi/ | [3] |
| *Fusarium graminearum* | AACM00000000 | www.broadinstitute.org/annotation/genome/fusarium_graminearum/ | [4] |
| *Microsporum canis* CBS 113480 | ABVF00000000 | www.broadinstitute.org/annotation/genome/dermatophyte_comparative/ | Dermatophyte Comparative Sequencing Project, Broad Institute of Harvard and MIT |
| *Neurospora crassa* OR74A | AABX00000000 | www.broadinstitute.org/annotation/genome/neurospora/MultiDownloads.html | [5] |
| *Pyrenophora tritici-repentis* Pt-1C-BFP | AAXI00000000 | www.broadinstitute.org/annotation/genome/pyrenophora_tritici_repentis.3/ | Joint Genome Institute |
| *Trichophyton rubrum* CBS 118892 | ACPH00000000 | www.broadinstitute.org/annotation/genome/dermatophyte_comparative | Dermatophyte Comparative Sequencing Project, Broad Institute of Harvard and MIT |
| *Uncinocarpus reesii* UAMH 1704 | AAIW00000000 | www.broadinstitute.org/annotation/genome/uncinocarpus_reesii.3/ | [3] |
|  |  |  |  |
| BASIDIOMYCOTA |  |  |  |
| *Coprinopsis cinerea* A7 #130 | AACS00000000 | www.broadinstitute.org/annotation/genome/coprinus_cinereus/ | [6] |
| *Cryptococcus neoformans* *var. grubii* H99 | AACO00000000 | www.broadinstitute.org/annotation/genome/cryptococcus_neoformans/ | *Cryptococcus neoformans* var. grubii H99 Sequencing Project, Broad Institute of Harvard and MIT |
| *Puccinia graminis* f.sp. tritici CRL 75-36-700-3 | AAWC00000000 | www.broadinstitute.org/annotation/genome/puccinia_group/ | [7] |
| *Ustilago maydis* | AACP00000000 | www.broadinstitute.org/annotation/genome/ustilago_maydis.2/ | [8] |
|  |  |  |  |
| ZYGOMYCOTA |  |  |  |
| *Phycomyces blakesleeanus* NRRL1555 | Not Available | genome.jgi-psf.org/Phybl2/Phybl2.download.ftp.html | Joint Genome Institute |
|  |  |  |  |
| BLASTOCLADIOMYCOTA |  |  |  |
| *Allomyces macrogynus* ATCC 38327 | ACDU00000000 | www.broadinstitute.org/annotation/genome/multicellularity_project/ | Origins of Multicellularity Sequencing Project, Broad Institute of Harvard and MIT |
|  |  |  |  |
| CHYTRIDIOMYCOTA |  |  |  |
| *Batrachochytrium dendrobatidis* |  |  |  |
| JAM81 | ADAR00000000 | genome.jgi-psf.org/Batde5/Batde5.download.ftp.html | Joint Genome Institute |
| JEL423 | AATT00000000 | www.broadinstitute.org/annotation/genome/batrachochytrium_dendrobatidis/ | *Batrachochytrium dendrobatidis* Sequencing Project, Broad Institute of Harvard and MIT |
| *Homolaphlyctis polyrhiza* | AFSM00000000 |  |  |
| *Spizellomyces punctatus* DAOM BR117 | ACOE00000000 | www.broadinstitute.org/annotation/genome/multicellularity_project/ | Origins of Multicellularity Sequencing Project, Broad Institute of Harvard and MIT |

REFERENCES

1. Burmester A, Shelest E, Glöckner G, Heddergott C, Schindler S, et al. (2011) Comparative and functional genomics provide insights into the pathogenicity of dermatophytic fungi. Genome Biology 12:R7: 16.

2. Galagan JE, Calvo SE, Cuomo C, Ma LJ, Wortman JR, et al. (2005) Sequencing of *Aspergillus nidulans* and comparative analysis with *A. fumigatus* and *A. oryzae*. Nature 438: 1105-1115.

3. Sharpton TJ, Stajich JE, Rounsley SD, Gardner MJ, Wortman JR, et al. (2009) Comparative genomic analyses of the human fungal pathogens *Coccidioides* and their relatives. Genome Res 19: 1722-1731.

4. Cuomo CA, Gueldener U, Xu JR, Trail F, Turgeon BG, et al. (2007) The *Fusarium graminearum* genome reveals a link between localized polymorphism and pathogen specialization. Science 317: 1400-1402.

5. Galagan JE, Calvo SE, Borkovich KA, Selker EU, Read ND, et al. (2003) The genome sequence of the filamentous fungus *Neurospora crassa*. Nature 422: 859-868.

6. Stajich JE, Wilke SK, Ahrén D, Au CH, Birren BW, et al. (2010) Insights into evolution of multicellular fungi from the assembled chromosomes of the mushroom *Coprinopsis cinerea* (*Coprinus cinereus*). Proc Natl Acad Sci U S A 107: 11889-11894.

7. Duplessis S, Cuomo CA, Lin Y, Aerts A, Tisserant E, et al. (2011) Obligate biotrophy features unraveled by the genomic analysis of rust fungi. Proc Natl Acad Sci U S A 108: 9166–9171.

8. Kämper J, Kahmann R, Bölker M, Ma LJ, Brefort T, et al. (2006) Insights from the genome of the biotrophic fungal plant pathogen *Ustilago maydis*. Nature 444: 97-101.
