# Supplementary material for: Genomic Transition to Pathogenicity in Chytrid Fungi
Source: PLoS Pathog. 2011 Nov 3;7(11):e1002338. doi: 10.1371/journal.ppat.1002338 (PMC3207900; doi:10.1371/journal.ppat.1002338)
Supplement: Table S5 — Gene family members of A) M36, B) S41, C) Asp protease and D) CRN-like gene family members for: A. macrogynus, B. dendrobatidis, H. polyrhiza, and S. punctatus. (DOC) [file ppat.1002338.s007.doc]

Table S5: Gene family members of A) metalloprotease, B) serine-type protease, C) aspartyl protease and D) CRN-like gene family members for: *Allomyces macrogynus, Batrachochytrium dendrobatidis*, *Homolaphlyctis polyrhiza,* and *Spizellomyces punctatus*.

5A. Metalloprotease gene family members.

*Allomyces macrogynus*

AMAG_00149T0

AMAG_07740T0

AMAG_07748T0

AMAG_09753T0

AMAG_09761T0

AMAG_10695T0

AMAG_11091T0

AMAG_11092T0

AMAG_11093T0

AMAG_11126T0

AMAG_11128T0

AMAG_11133T0

AMAG_11353T0

AMAG_14366T0

AMAG_14954T0

AMAG_15202T0

AMAG_16399T0

AMAG_16400T0

AMAG_16401T0

AMAG_16405T0

AMAG_16408T0

AMAG_16410T0

AMAG_16411T0

AMAG_16416T0

AMAG_16418T0

AMAG_16424T0

AMAG_16427T0

AMAG_16443T0

AMAG_16963T0

AMAG_17346T0

AMAG_17415T0

*Batrachochytrium dendrobatidis –* JAM81

BATDEDRAFT_1469 gw1.9.61.1

BATDEDRAFT_1480 gw1.5.118.1

BATDEDRAFT_1483 gw1.6.81.1

BATDEDRAFT_1489 gw1.6.82.1

BATDEDRAFT_1501 gw1.3.138.1

BATDEDRAFT_1502 gw1.5.120.1

BATDEDRAFT_1593 gw1.9.71.1

BATDEDRAFT_1614 gw1.15.31.1

BATDEDRAFT_1616 gw1.15.32.1

BATDEDRAFT_1639 gw1.12.38.1

BATDEDRAFT_5299 gw1.2.397.1

BATDEDRAFT_5302 gw1.15.141.1

BATDEDRAFT_5304 gw1.12.163.1

BATDEDRAFT_5305 gw1.2.398.1

BATDEDRAFT_5317 gw1.2.399.1

BATDEDRAFT_5343 gw1.17.83.1

BATDEDRAFT_5365 gw1.16.153.1

BATDEDRAFT_5372 gw1.15.144.1

BATDEDRAFT_5399 gw1.9.265.1

BATDEDRAFT_11084 e_gw1.5.127.1

BATDEDRAFT_11205 e_gw1.5.117.1

BATDEDRAFT_11777 e_gw1.7.89.1

BATDEDRAFT_12637 e_gw1.9.62.1

BATDEDRAFT_14272 e_gw1.15.138.1

BATDEDRAFT_16271 estExt_Genewise1.C_40190

BATDEDRAFT_16613 estExt_Genewise1.C_60139

BATDEDRAFT_23127 fgenesh1_pg.C_scaffold_2000514

BATDEDRAFT_27765 fgenesh1_pg.C_scaffold_14000219

BATDEDRAFT_28410 fgenesh1_pg.C_scaffold_18000063

BATDEDRAFT_33981 estExt_fgenesh1_pg.C_10347

BATDEDRAFT_34483 estExt_fgenesh1_pg.C_20452

BATDEDRAFT_35259 estExt_fgenesh1_pg.C_70223

BATDEDRAFT_36120 estExt_fgenesh1_pg.C_150175

BATDEDRAFT_36196 estExt_fgenesh1_pg.C_170044

BATDEDRAFT_85535 GP3.010940

BATDEDRAFT_87065 GP3.026240

BATDEDRAFT_90246 GP3.058050

BATDEDRAFT_92063 GP3.076220

*Homolaphlyctis polyrhiza* (Hp_Maker gene names start with either snap or mkr, and Hp_GeneMark genes start with gm)

gm_ctg00587-0.7

gm_asm134-0.164

gm_asm210-2.29

snap_msk_ctg00587-0.5

mkr_asm210-snap-2.3

*Spizellomyces punctatus*

SPPG_01665T0

SPPG_02641T0

SPPG_04344T0

5B. Serine-type protease gene family members.

*Allomyces macrogynus*

NA

*Batrachochytrium dendrobatidis –* JAM81

BATDEDRAFT_22176 fgenesh1_pg.C_scaffold_1001049

BATDEDRAFT_23310 fgenesh1_pg.C_scaffold_3000106

BATDEDRAFT_23534 fgenesh1_pg.C_scaffold_3000330

BATDEDRAFT_23544 fgenesh1_pg.C_scaffold_3000340

BATDEDRAFT_23754 fgenesh1_pg.C_scaffold_3000550

BATDEDRAFT_24156 fgenesh1_pg.C_scaffold_4000395

BATDEDRAFT_24207 fgenesh1_pg.C_scaffold_4000446

BATDEDRAFT_24985 fgenesh1_pg.C_scaffold_6000214

BATDEDRAFT_25222 fgenesh1_pg.C_scaffold_6000451

BATDEDRAFT_25462 fgenesh1_pg.C_scaffold_7000214

BATDEDRAFT_25463 fgenesh1_pg.C_scaffold_7000215

BATDEDRAFT_25559 fgenesh1_pg.C_scaffold_7000311

BATDEDRAFT_26098 fgenesh1_pg.C_scaffold_9000010

BATDEDRAFT_26287 fgenesh1_pg.C_scaffold_9000199

BATDEDRAFT_27246 fgenesh1_pg.C_scaffold_12000195

BATDEDRAFT_27937 fgenesh1_pg.C_scaffold_15000161

BATDEDRAFT_28623 fgenesh1_pg.C_scaffold_20000019

BATDEDRAFT_28625 fgenesh1_pg.C_scaffold_20000021

BATDEDRAFT_28775 fgenesh1_pg.C_scaffold_35000008

BATDEDRAFT_35365 estExt_fgenesh1_pg.C_80073

BATDEDRAFT_37569 estExt_fgenesh1_kg.C_200009

BATDEDRAFT_85649 GP3.012080

BATDEDRAFT_86001 GP3.015600

BATDEDRAFT_86231 GP3.017900

BATDEDRAFT_86314 GP3.018730

BATDEDRAFT_87928 GP3.034870

BATDEDRAFT_89445 GP3.050040

BATDEDRAFT_90146 GP3.057050

BATDEDRAFT_92476 GP3.080350

BATDEDRAFT_92744 GP3.083030

BATDEDRAFT_92841 GP3.084000

BATDEDRAFT_93324 GP3.088830

*Homolaphlyctis polyrhiza* (Hp_Maker genes only)

gm_asm106-0.164_1

gm_asm236-0.161_1

gm_asm80-0.52_1

*Spizellomyces punctatus*

SPPG_00409T0

SPPG_05166T0

SPPG_06219T0

5C. Aspartyl protease gene family members.

*Allomyces macrogynus*

AMAG_11032T0

AMAG_11644T0

AMAG_12075T0

AMAG_08963T0

AMAG_08962T0

AMAG_12076T0

*Batrachochytrium dendrobatidis –* JAM81

BATDEDRAFT_4465

BATDEDRAFT_12559

BATDEDRAFT_12639

BATDEDRAFT_16209

BATDEDRAFT_20107

BATDEDRAFT_20117

BATDEDRAFT_21133

BATDEDRAFT_21660

BATDEDRAFT_22586

BATDEDRAFT_22593

BATDEDRAFT_22611

BATDEDRAFT_22618

BATDEDRAFT_22623

BATDEDRAFT_23192

BATDEDRAFT_23205

BATDEDRAFT_23213

BATDEDRAFT_23274

BATDEDRAFT_23275

BATDEDRAFT_23759

BATDEDRAFT_23765

BATDEDRAFT_24293

BATDEDRAFT_24300

BATDEDRAFT_24380

BATDEDRAFT_24760

BATDEDRAFT_24767

BATDEDRAFT_25148

BATDEDRAFT_25223

BATDEDRAFT_25251

BATDEDRAFT_25259

BATDEDRAFT_25355

BATDEDRAFT_25617

BATDEDRAFT_25666

BATDEDRAFT_25669

BATDEDRAFT_25680

BATDEDRAFT_25784

BATDEDRAFT_26088

BATDEDRAFT_26132

BATDEDRAFT_26134

BATDEDRAFT_26151

BATDEDRAFT_26411

BATDEDRAFT_26425

BATDEDRAFT_26741

BATDEDRAFT_26748

BATDEDRAFT_26758

BATDEDRAFT_26762

BATDEDRAFT_27037

BATDEDRAFT_27052

BATDEDRAFT_27069

BATDEDRAFT_27277

BATDEDRAFT_27286

BATDEDRAFT_27290

BATDEDRAFT_27292

BATDEDRAFT_27296

BATDEDRAFT_27300

BATDEDRAFT_27767

BATDEDRAFT_27966

BATDEDRAFT_28166

BATDEDRAFT_28176

BATDEDRAFT_28181

BATDEDRAFT_28256

BATDEDRAFT_28328

BATDEDRAFT_28513

BATDEDRAFT_28537

BATDEDRAFT_28541

BATDEDRAFT_28684

BATDEDRAFT_28688

BATDEDRAFT_28725

BATDEDRAFT_28777

BATDEDRAFT_28842

BATDEDRAFT_28984

BATDEDRAFT_35725

BATDEDRAFT_35728

BATDEDRAFT_35816

BATDEDRAFT_86720

BATDEDRAFT_87177

BATDEDRAFT_87185

BATDEDRAFT_87250

BATDEDRAFT_87859

BATDEDRAFT_87892

BATDEDRAFT_88273

BATDEDRAFT_89345

BATDEDRAFT_89380

BATDEDRAFT_89821

BATDEDRAFT_89831

BATDEDRAFT_89959

BATDEDRAFT_90236

BATDEDRAFT_90267

BATDEDRAFT_90524

BATDEDRAFT_90577

BATDEDRAFT_90625

BATDEDRAFT_90861

BATDEDRAFT_90888

BATDEDRAFT_90978

BATDEDRAFT_91075

BATDEDRAFT_92020

BATDEDRAFT_92488

BATDEDRAFT_92592

BATDEDRAFT_92738

BATDEDRAFT_92765

*Homolaphlyctis polyrhiza* (Hp_GeneMark genes only)

gm_asm116-0.104

gm_asm117-0.23

gm_asm117-0.25

gm_asm242-0.207

gm_asm268-0.190

gm_asm336-1.205

gm_asm375-0.190

gm_ctg01127-0.10

gm_ctg01127-0.11

gm_ctg01303-0.6

gm_ctg01303-0.7

gm_ctg01383-0.6

gm_ctg02263-0.0

gm_ctg02264-0.0

gm_ctg03150-0.14

gm_ctg03169-0.2

gm_ctg07229-0.0

gm_ctg07303-0.1

gm_ctg08666-0.8

gm_ctg09870-0.5

gm_ctg10038-0.4

gm_ctg13909-0.3

*Spizellomyces punctatus*

SPPG_01941T0

SPPG_00681T0

SPPG_07923T0

SPPG_08789T0

SPPG_08444T0

SPPG_04035T0

SPPG_02558T0

SPPG_00268T0

SPPG_05983T0

SPPG_06901T0

5D. CRN-like gene family members.

*Allomyces macrogynus*

NA

*Batrachochytrium dendrobatidis –* JAM81

BATDEDRAFT_22610 fgenesh1_pg.C_scaffold_1001483

BATDEDRAFT_23071 fgenesh1_pg.C_scaffold_2000458

BATDEDRAFT_23077 fgenesh1_pg.C_scaffold_2000464

BATDEDRAFT_23206 fgenesh1_pg.C_scaffold_3000002

BATDEDRAFT_23217 fgenesh1_pg.C_scaffold_3000013

BATDEDRAFT_24333 fgenesh1_pg.C_scaffold_5000049

BATDEDRAFT_24378 fgenesh1_pg.C_scaffold_5000094

BATDEDRAFT_24764 fgenesh1_pg.C_scaffold_5000480

BATDEDRAFT_24811 fgenesh1_pg.C_scaffold_6000040

BATDEDRAFT_25057 fgenesh1_pg.C_scaffold_6000286

BATDEDRAFT_25352 fgenesh1_pg.C_scaffold_7000104

BATDEDRAFT_25664 fgenesh1_pg.C_scaffold_7000416

BATDEDRAFT_26085 fgenesh1_pg.C_scaffold_8000417

BATDEDRAFT_26137 fgenesh1_pg.C_scaffold_9000049

BATDEDRAFT_26152 fgenesh1_pg.C_scaffold_9000064

BATDEDRAFT_26156 fgenesh1_pg.C_scaffold_9000068

BATDEDRAFT_26410 fgenesh1_pg.C_scaffold_9000322

BATDEDRAFT_26749 fgenesh1_pg.C_scaffold_11000004

BATDEDRAFT_26962 fgenesh1_pg.C_scaffold_11000217

BATDEDRAFT_26980 fgenesh1_pg.C_scaffold_11000235

BATDEDRAFT_27205 fgenesh1_pg.C_scaffold_12000154

BATDEDRAFT_27291 fgenesh1_pg.C_scaffold_13000002

BATDEDRAFT_28183 fgenesh1_pg.C_scaffold_17000010

BATDEDRAFT_28329 fgenesh1_pg.C_scaffold_17000156

BATDEDRAFT_28349 fgenesh1_pg.C_scaffold_18000002

BATDEDRAFT_28463 fgenesh1_pg.C_scaffold_18000116

BATDEDRAFT_28466 fgenesh1_pg.C_scaffold_18000119

BATDEDRAFT_28523 fgenesh1_pg.C_scaffold_19000005

BATDEDRAFT_28683 fgenesh1_pg.C_scaffold_20000079

BATDEDRAFT_28687 fgenesh1_pg.C_scaffold_20000083

BATDEDRAFT_31422 fgenesh1_kg.C_scaffold_3000001

BATDEDRAFT_34275 estExt_fgenesh1_pg.C_11252

BATDEDRAFT_34850 estExt_fgenesh1_pg.C_40342

BATDEDRAFT_35207 estExt_fgenesh1_pg.C_70108

BATDEDRAFT_35892 estExt_fgenesh1_pg.C_120230

BATDEDRAFT_36061 estExt_fgenesh1_pg.C_140198

BATDEDRAFT_36778 estExt_fgenesh1_kg.C_40005

BATDEDRAFT_36889 estExt_fgenesh1_kg.C_50007

BATDEDRAFT_37012 estExt_fgenesh1_kg.C_70030

BATDEDRAFT_37407 estExt_fgenesh1_kg.C_130053

BATDEDRAFT_84882 GP3.004410

BATDEDRAFT_84908 GP3.004670

BATDEDRAFT_85109 GP3.006680

BATDEDRAFT_86464 GP3.020230

BATDEDRAFT_86517 GP3.020760

BATDEDRAFT_86546 GP3.021050

BATDEDRAFT_87128 GP3.026870

BATDEDRAFT_87221 GP3.027800

BATDEDRAFT_87524 GP3.030830

BATDEDRAFT_87953 GP3.035120

BATDEDRAFT_87954 GP3.035130

BATDEDRAFT_88318 GP3.038770

BATDEDRAFT_89826 GP3.053850

BATDEDRAFT_89833 GP3.053920

BATDEDRAFT_90343 GP3.059020

BATDEDRAFT_90726 GP3.062850

BATDEDRAFT_90966 GP3.065250

BATDEDRAFT_91239 GP3.067980

BATDEDRAFT_91252 GP3.068110

BATDEDRAFT_91751 GP3.073100

BATDEDRAFT_92602 GP3.081610

BATDEDRAFT_92692 GP3.082510

*Homolaphlyctis polyrhiza* Hp_GeneMark

NA

*Spizellomyces punctatus*

NA
